# Long-range data transmission in a fault-tolerant quantum bus architecture

**DOI:** 10.1038/s41534-024-00928-4

**Published:** 2024-12-26

**Authors:** Shin Ho Choe, Robert König

**Affiliations:** 1 Department of Mathematics, School of Computation, Information and Technology, Technical University of Munich, Garching, Germany; 2 Munich Center for Quantum Science and Technology (MCQST), Munich, Germany

**Keywords:** Quantum information, Information theory and computation

## Abstract

We propose a fault-tolerant scheme for generating long-range entanglement at the ends of a rectangular array of qubits of length *R* with a square cross-section of $m=O({\log }^{2}R)$ qubits. It is realized by a constant-depth circuit producing a constant-fidelity Bell-pair (independent of *R*) for local stochastic noise of strength below an experimentally realistic threshold. The scheme can be viewed as a quantum bus in a quantum computing architecture where qubits are arranged on a rectangular 3D grid, and all operations are between neighboring qubits. Alternatively, it can be seen as a quantum repeater protocol along a line, with neighboring repeaters placed at a short distance to allow constant-fidelity nearest-neighbor operations. To show our protocol uses a number of qubits close to optimal, we show that any noise-resilient distance-*R* entanglement generation scheme realized by a constant-depth circuit needs at least $m=\Omega (\log R)$ qubits per repeater.

## Introduction

The ability of operating on spatially separated qubits has been recognized early on as a key requirement for a working quantum computer^1,2^. Long-range quantum communication is also a fundamental primitive at the core of quantum information-theoretic^3–5^ and cryptographic^6,7^ protocols in quantum networks, as well as e.g., distributed quantum computation^8–10^ or distributed sensing^11,12^. With increasingly powerful capabilities of present-day devices that can act as local nodes in such a network, finding appropriate protocols for entanglement sharing and quantum communication over faulty networks remains an active and central theme of present-day research.

Here we focus on long-range entanglement generation schemes which can be used in quantum computing devices where two-qubit operations are restricted to be geometrically local. This kind of restriction typically arises from technological constraints and is determined by the geometric arrangement of qubits in space as well as limits on the ability to couple distant qubits. Quantum algorithmic and fault-tolerance-related constructions, on the other hand, typically do not refer to notions of geometric locality. For example, while recent quantum low-density parity check (LDPC) codes are seen as excellent candidates for resource-efficient realizations of fault-tolerant quantum computing, their application involves long-range interactions when qubits are embedded in Euclidean space: A recent result implies that the number of such long-range gates needed scales at least polynomially with the size of the code^13^. This motivates the need to design protocols for realizing long-range interactions. Using teleportation, this goal can be rephrased as that of generating a long-range Bell pair in a short time with local operations. Any corresponding construction, such as ours, can be used as a quantum (data) bus in scalable quantum devices^14^.

Our proposal addresses the specific constraints on near-term devices by optimizing the following aspects:

(i) qubit efficiency versus distance: Our protocol fault-tolerantly generates a Bell pair on two qubits separated by distance *R*, using a grid of qubits of size *d* × *d* × *R* with $d=O(\log R)$. That is, it uses only $O(R{\log }^{2}R)$ qubits in total.
(ii) simplicity (geometric locality) of operations: Our scheme only involves one- and two-qubit gates between nearest-neighbor qubits in the architecture. The architecture could be realized, e.g., by stacking planar chip-like devices, and connecting neighboring devices through the third dimension.
(iii) resilience to general errors: we provide a rigorous proof of the robustness of our scheme in the form of a fault-tolerance threshold theorem allowing for fully general (circuit-level) local stochastic noise^15^. In this model, a random (multiqubit) Pauli error *E*_*t*_ acts on the system at each time step *t* of the quantum circuit. The probability that the Pauli error *E*_*t*_ at each time step *t* acts non-trivially on any subset of *k* qubits is assumed to be at most *p*^*k*^ for some *p* ∈ [0, 1]. The parameter *p* is called the strength of the local stochastic noise, and we write ${E}_{t} \sim {\mathcal{N}}(p)$. This notion only depends on the marginal distribution of *E*_*t*_. In particular, our analysis captures situations where errors are correlated both in space and time, and we assume all involved operations (state preparation, gates and measurements) to be non-ideal, i.e., affected by noise. We note that – because of the simplicity of the scheme — we can explicitly specify and analyze all involved operations without resorting to fault-tolerant gadgets (e.g., for encoding and recovery) that are often treated in a black-box manner.
(iv) short coherence times and minimal latency: The required coherence time of each qubit is independent of the overall communication distance *R*: It only needs to be of the order of the local operation time *t*_local_. This is the maximal time needed to perform a single- or two-qubit gate, a single-qubit measurement, or an entangling gate between two neighboring qubits.
  Our protocol minimizes the amount of time *T*_ent_ it takes to establish long-range entanglement from a product state of all qubits. The long-range entangled state is available after a time of order *T*_ent_ = *O*(*t*_local_).

**Fig. 1:**
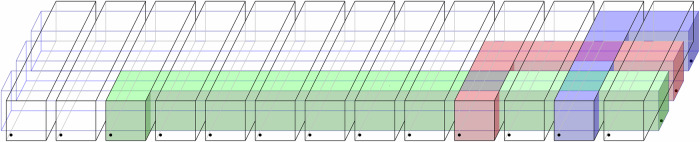
A quasi-2D network of quantum buses. This architecture allows to perform a joint measurement on any subset of *ℓ* qubits (illustrated here for *ℓ* = 3) with constant fidelity by “busing” the corresponding qubits to the same location before the measurement, and subsequently transferring the qubits back. The transfer is achieved by teleporting the qubits using the long-range entanglement established with our protocol on each bus.

In the area of quantum computing architectures, our protocol could be especially beneficial in settings where gates or measurements need to be performed between spatially distant qubits. Such a need arises for example when using a quantum low-density parity check (LDPC) code whose stabilizer generators are not spatially local. With our scheme, we can design a quasi-2D architecture that allows for the application of joint measurements on any subset of size *ℓ* = *O*(1) of a total of *n* qubits, see Fig. 1. Each such *ℓ*-qubit measurement is executed with constant fidelity in a constant amount of time (up to Pauli correction whose classical postprocessing can typically be deferred to a later stage). The architecture requires $O(n\cdot {\mathsf{polylog}}(n))$ qubits in total. When combined with the recently discovered good quantum LDPC codes that have parameters close to optimal^16^, this logarithmic overhead only leads to an encoding rate that decays as an inverse polylogarithmic function.

Our scheme provides a candidate architecture for transferring quantum information within a quantum device, but can alternatively also be used to transfer information between devices. In this setting, one typically considers a line of *R* equidistantly placed repeaters, where each repeater controls *m* qubits, neighboring repeaters are connected by (imperfect) quantum communication as well as classical communication links, and the goal is to establish entanglement between the first and the last repeater (separated by a distance proportional to *R* − 1). Assuming that every repeater has full control over its qubits, i.e., can apply any quantum circuit, this setup allows (by teleportation between repeaters) to imperfectly realize an adaptive quantum circuit on *mR* qubits which only uses gates between pairs of qubits associated with neighboring repeaters, and gates acting only on its own qubits. Any repeater protocol for distance-*R* entanglement generation amounts to a circuit of this form, and, conversely, circuits with these restrictions can be seen as repeater protocols.

In our architecture, qubits are located on the sites of a slab-like 3D rectangular lattice of dimensions *d* × *d* × *R*. We can define, for each *j* ∈ {1, …, *R*}, a repeater ${{\mathcal{R}}}_{j}$ which is composed of the qubits in the “slice” {1, …, *d*} × {1, …, *d*} × {*j*}. Then our circuit construction immediately translates into a repeater protocol with the following desirable features for each repeater:

- long communication distance: As a function of the total communication distance *R*, every repeater only needs to manipulate $m=O({\log }^{2}R)$ number of qubits. This is in line with the best currently known proposals^17,18^ which have poly-logarithmic scaling for long-range quantum communication. We also provide a converse bound $\Omega (\log R)$ on the number of qubits per repeater for low-latency protocols as considered in this work.
- simplicity of involved operations: Assuming the repeaters are connected appropriately (by *m* links along the direction of communication), all operations at each repeater ${{\mathcal{R}}}_{j}$, *j* ∈ {1…, *R*} are geometrically local when the $m=O({\log }^{2}R)$ qubits are arranged on a 2D array.
  In contrast, in existing works on repeater-based quantum communication, repeaters are often assumed to provide universal quantum computing power locally, and no geometric locality considerations are taken into account. For example, in ref. ^19^, the authors discuss entanglement distribution protocols which use quantum error-correcting codes^17,18,20,21^. This family of protocols proceeds by communicating encoded quantum information: Repeaters apply a recovery operation to each codeblock before retransmitting the entire block to the next repeater. This operation is arguably more involved that the operations required in our scheme.
  Purely error-correction based schemes have a loss-tolerance limited by 50% because of the no-cloning theorem. To sidestep this limitation, the more recent work^22^ combines error correction with hashing-based entanglement purification. In this scheme, intermediate error correction operations are realized based on stabilizer (resource) states prepared at each repeater. The proposed scheme achieves a constant rate of qubit transmission. Here error correction is based on measurement-based gate teleportation using special (graph) states encoding the recovery map at each repeater location. While such schemes are ultimately desirable, the generation of these resource states is arguably more challenging to realize with near-term devices than the protocol discussed here. In particular, protocols preparing these resource states require long-range gates and non-constant circuit depth in each repeater. These operations are more involved that those in our scheme, and, in particular, do not give a low-latency scheme because of the extensive circuit depth.
- resilience to full circuit-level local stochastic noise: It is sufficient if each repeater realizes circuits up to local stochastic noise. In particular, no fault-tolerant quantum computer is needed at any one of the repeaters.
  In contrast to our circuit-level analysis, existing proposals often resort to the black-box use of fault-tolerant gadgets (e.g., for encoding and recovery), essentially amounting to the assumption that each repeater has full quantum fault-tolerance capabilities. Furthermore, existing works on quantum repeaters argue that faulty operations can be modeled by erroneous communication links (or corresponding Bell states) by commuting errors forward respectively backward in time. Errors arising in this way are generally not going to identically and independently distributed. Nevertheless, the provided analysis in existing works typically assumes this i.i.d. form of the noise. Our work overcomes this mismatch between the actually occuring noise and the error analysis by avoiding such simplistic assumptions altogether.
- constant coherence times and minimal latency: Every repeater only needs to maintain coherence for a time of order *t*_local_. Furthermore, the long-range entangled state is available after a time of order *T*_ent_ = *O*(*t*_local_). In particular, no quantum memory with a scalable qubit lifetime is required.
  In contrast, the time *T*_ent_ to establish long-range entanglement scales at least linearly with the total communication distance *R* in several existing protocols based, e.g., on transmitting encoded information successively between neighboring repeaters^18,21^. We note that these protocols reach the scaling *O*(*t*_local_) per generated Bell pair asymptotically when operated continuously, i.e., this is an achievable rate of communication. In contrast, the quantity *T*_ent_ measures the latency when starting to run the scheme, and may be particularly relevant in near-term applications in scenarios where continuous operation is challenging.
  Entanglement distribution protocols satisfying a low-latency property can be considered as those with repeaters that cannot store quantum information fault-tolerantly for a long time. In ref. ^23^, entanglement distribution protocols are classified into the following two groups: The first group involves quantum memories of limited lifetime^24–26^, whereas the second group relies on quantum memories with sufficiently long lifetimes^23,27–35^. The protocols belonging to the first group are closer to our criteria. We emphasize, however, that our protocol does not assume the availability of any quantum memory: all qubits, including idling ones, are assumed to be affected by noise.

While these properties are desirable features of quantum repeater protocols, we emphasize that our construction applies only to a certain regime where the inter-repeater links are relatively noise-free. This is a fundamental difference between the noise model considered here, and that typically studied in the long-distance quantum communication setting. It is only because of our assumptions on the noise model that we can achieve the low-latency property (d).

In more detail, most existing works discussing long-distance entanglement distribution consider error models motivated by quantum communication applications, e.g., transmission of qubits over an optical fiber. To highlight the main difference to our setup, consider first the error strength. In the optical setup, it is typically assumed that the fidelity of entangling gates between neighboring repeaters is very low. This assumption is meaningful when repeaters are separated by a certain distance, and entanglement is established by distributing a (physical) Bell state through the optical fiber. The loss (or error) rate then depends on the distance between repeaters, and can be any (small) constant for a fixed inter-repeater distance. This issue is typically addressed by generating higher-fidelity Bell pairs between repeaters by means of extra processes such as entanglement distillation. The complexity of these distillation steps precludes the possibility of a generation scheme having the low-latency property (d) in the setting of high error rates. (We refer to ref. ^19^ for a comparative study of different approaches to long-distance entanglement distribution. This includes a classification of protocols into three generations and a discussion of what kind of noise is handled by each generation of protocols).

In contrast, we assume constant-strength local stochastic noise – which, in the setting of repeaters, roughly means that entangling gates between neighboring repeaters can be realized with a fidelity above some threshold value. Such a regime of low error rates corresponds to a setting where the spacing between repeaters is limited, i.e., they cannot be arbitrarily far apart. In this case, low-latency long-distance entanglement is possible.

Our setup (as described by (c)) differs in another important aspect from commonly made assumptions in quantum communications: Our analysis also applies to correlated errors. This makes our protocol particularly attractive for using it as a quantum bus (rather than for point-to-point communication), as quantum computations can propagate errors leading to correlations between different timesteps. Analysing only independent errors will generally not be sufficient to ensure the protocol is suitable for use in noisy quantum computational devices.

**Fig. 2:**
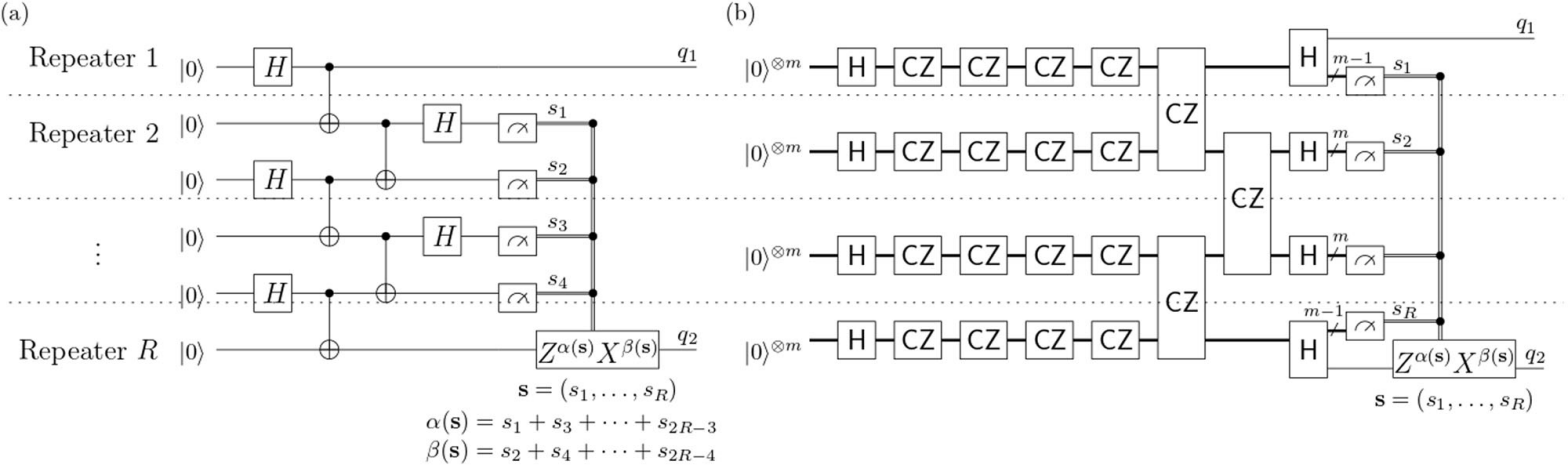
Long-distance entanglement generation protocols with low latency expressed as quantum circuits. Every gate acts on qubits within a single repeater or on a pair of qubits associated with two neighboring repeaters. Each construction has a constant circuit depth independent of the entanglement distance *R*. (**a**) Entanglement swapping circuit. It generates a Bell state of two qubits indexed by *q*_1_ and *q*_2_ with certainty, but it is not fault-tolerant under noise, such as i.i.d. measurement error. (**b**) Our protocol. Each repeater has *m* qubits. The gates ${\mathsf{H}}$ and ${\mathsf{CZ}}$ are tensor products of Hadamard gates and controlled-Z gates, respectively, on certain subsets of qubits clarified in Result.

To motivate the problem of fault-tolerant long-distance entanglement generation with low latency, let us recall entanglement swapping. We consider *R* sites (or “repeaters”) lying on a line, where each pair of neighboring sites shares a Bell state $\left\vert \Phi \right\rangle :=(\left\vert 00\right\rangle +\left\vert 11\right\rangle )/\sqrt{2}$, see Fig. 2(a). Suppose we measure two qubits at each site in the Bell basis. Then, the resulting two-qubit state on two opposite sites on the line is one of the Bell basis states, which is determined by the measurement results. By applying an appropriate Pauli correction operator, we obtain the Bell state $\left\vert \Phi \right\rangle$ with certainty if all operations are perfect.

Here, we may replace the preshared Bell pairs in this protocol with the computational basis state followed by a Hadamard gate and a CNOT gate, and we can also replace Bell measurements with entangling gates followed by the computational basis measurements. Then the protocol can be written as an adaptive quantum circuit of constant depth, where two-qubit gates act on neighboring sites only. Since the depth is constant, the latency of the protocol without the correction remains constant even if *R* increases, thus giving us property (d).

However, entanglement swapping is not fault-tolerantly scalable in the presence of noise. Suppose we have i.i.d. noise on the measurement results, i.e., there is a non-zero constant *p* such that each measurement result is flipped with probability *p*, independently. Then, the probability that the resulting state is *Φ* exponentially converges to 1/4 as the communication distance increases. This motivates us to introduce more qubits per site to make protocols fault-tolerant; see the initial state of the protocol in Fig. 2(b).

The protocol we propose here uses quantum error correction, and is a close relative of the protocols discussed in ref. ^19^. Indeed, one interpretation of the corresponding architecture is that it encodes logical information into a surface code at each repeater. We emphasize, however, that this only motivates the scheme in a similar way, e.g., cluster-state measurement-based quantum computation is related to braiding surface code anyons. For example, the scheme does not involve intermediate encoding/recovery operations associated with these surface codes.

## Results

Our work builds on the fault-tolerance properties of the 3D cluster state. In seminal work ref. ^36^, Raussendorf, Bravyi and Harrington proved that for temperatures below some threshold, the thermal state of the cluster state Hamiltonian on a simple 3D cubic lattice has localizable entanglement: applying single-qubit Pauli measurements to each “bulk” qubit results – up to Pauli corrections determined by the measurement outcomes – in an encoded Bell state of 2D surface codes lying on two opposing faces of the cube. Since the initial thermal state is the cluster state with independent and identically distributed Pauli-*Z*-errors on each qubit, and the cluster state can be created in constant depth with local operations, this provides a scheme for long-range (encoded) entanglement generation by a constant-depth circuit with local gates. While the assumed (physically motivated) noise model is somewhat restricted, the authors of ref. ^36^ also point out that more general errors on the initial cluster state such as independent single-qubit depolarizing noise can be tolerated when using ideal (non-faulty) operations at the two surfaces.

The scheme of ref. ^36^ produces a surface-code encoded Bell pair, i.e., the resulting entanglement lives in a logical 2-qubit subspace of a many-qubit system. In applications such as the one considered in ref. ^37^, this is a desirable feature: the encoded information can be operated on without decoding, thus preserving fault-tolerance properties. In a communication scenario, however, it may be more desirable to establish entanglement between two physical qubits instead. A scheme for achieving this kind of localization of the entanglement was proposed in ref. ^38^ by modifying the approach of ref. ^36^: Here a different single-qubit measurement pattern is used at the two (“surface code”) boundaries. This results in entanglement between two distinguished qubits in each surface. For the problem of establishing a constant-fidelity Bell pair in a cube of size *R* × *R* × *R*, the work ref. ^38^ provides analytical and numerical threshold estimates in the case of i.i.d. Pauli-*X* and Pauli-*Z* errors in the cluster state. (It was argued in ref. ^38^ that this error model captures a situation where initially, independent noisy Bell states are shared along each edge of the cubic lattice, and all subsequent (single-qubit) operations are ideal, i.e., noise-free.) In particular, it is shown that there is a threshold error rate such that constant-fidelity two-qubit entanglement (i.e., independent of *R*) is established for any error rate below that threshold.

To achieve a longer communication distance, one can use cluster states on lattices of the form *d* × *d* × *R* with *R* ≫ *d*, i.e., lattices that are elongated in the direction where quantum information is to be transferred. This idea has been explored numerically in the case of i.i.d. Pauli-*Z* noise in ref. ^36^. In a similar vein, the results of ref. ^38^ are stated for cubes (i.e., lattices of the form *R* × *R* × *R*), but the author suggests using lattices of the form *d* × *d* × *R* with $d=\Theta (\log R)$.

The protocol based on the 3D cluster state satisfies the low-latency requirement (iv) and uses only geometrically local operations (cf. (ii)) by its construction. However, the achievable communication distance as a function of the number of qubits (see (i)) and the resilience to local stochastic noise (see (iii)) have not been addressed by the prior work^36,38^. Here we extend the result of refs. ^36,38^ and prove that the protocol based on the cluster state of the lattice of the form *d* × *d* × *R* is fault-tolerant under circuit-level local stochastic noise of strength below a constant threshold, even with *d* ≫ *R*. Our result shows that the protocol based on the 3D cluster state additionally satisfies properties (i) and (iii).

In the remainder of this section, we present our construction and the associated analysis. First, we describe the cluster-state-based protocol^38^ in detail, highlighting its low latency and the fact that it only uses geometrically local operations in 3D. Next, we establish rigorous bounds on the fault-tolerance threshold for the scenario where the protocol is run with *R* ≫ *d* under general circuit-level noise. We will show that a large distance *R* is achievable (cf. (a) and prove a fault-tolerance threshold result (cf. (c)). Subsequently, we discuss a fundamental limit to entanglement distribution protocols on a line under the low latency constraint: It takes the form of a lower bound on the number of qubits that each repeater must control. Next, we discuss an upper bound on the communication distance achievable with our 3D cluster state-based protocol, and compare it with general entanglement distribution protocols with low latency. Finally, we provide a modular way of understanding the protocol by introducing the protocol which fault-tolerantly transfers surface-code encoded information onto a single physical qubit.

### Low-latency long-range entanglement generation using a 3D cluster state

**Fig. 3:**
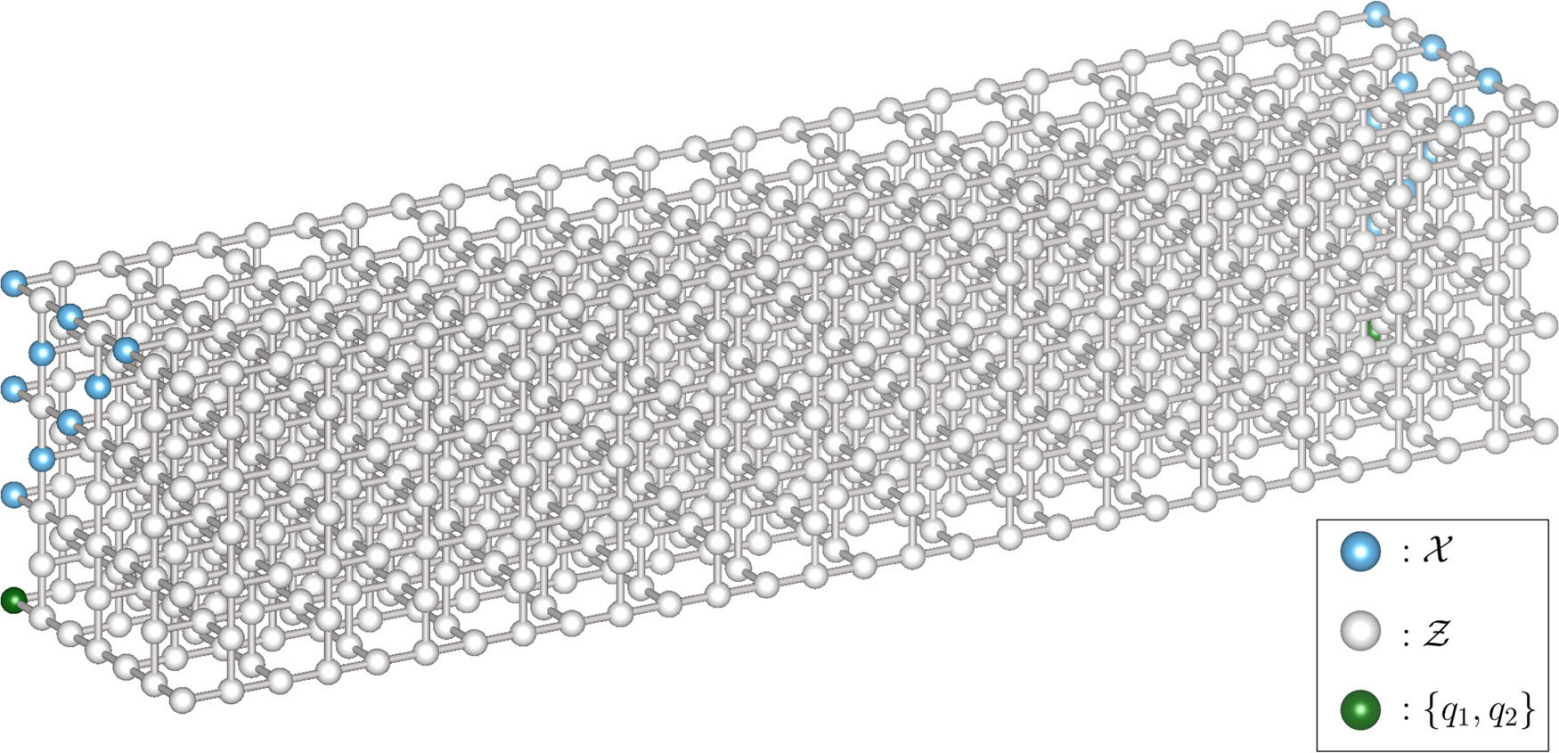
A cluster state on the lattice ${\mathcal{C}}[d\times d\times R]$ with *R*( = 29) ≫ *d*( = 4). Each sphere represents a qubit. Also indicated is the measurement pattern used in our protocol, consisting of single-qubit Pauli-*X* and Pauli-*Z* measurements. Entanglement is established between the qubits *q*_1_ and *q*_2_. The set of qubits is partitioned into three subsets, namely ${\mathcal{X}}$, ${\mathcal{Z}}$ and {*q*_1_, *q*_2_}. In the course of the protocol, the qubits in the subsets ${\mathcal{X}}$ and ${\mathcal{Z}}$ are measured in the Hadamard- and computational basis, respectively.

We present a protocol for generating a long-range entangled two-qubit Bell state in a fault-tolerant manner in the presence of local stochastic noise. We note that it is essentially identical to the protocol introduced in ref. ^38^, but we work with longer communication distance *R* ≫ *d*. Consider a lattice ${\mathcal{C}}={\mathcal{C}}[d\times d\times R]$ which is a subgraph of the 3D grid graph of size (2*d* − 1) × (2*d* − 1) × *R* (see Fig. 3). Edges of ${\mathcal{C}}$ are collected so that two vertices in ${\mathcal{C}}$ are adjacent if their Manhattan distance is equal to 1. Each vertex of the lattice ${\mathcal{C}}$ is occupied with one qubit which is initialized as $\left\vert 0\right\rangle ={{1}\choose{0}}\in {{\mathbb{C}}}^{2}$.

The goal of the protocol is to prepare a Bell pair $\left\vert \Phi \right\rangle$ on the qubits *q*_1_ and *q*_2_ which are located at two opposite corners of the lattice ${\mathcal{C}}$ (see Fig. 3). Each slice of size (2*d* − 1) × (2*d* − 1) is considered a node or a repeater. With this interpretation, the protocol describes a procedure of entanglement sharing between the two end nodes in a 1D network. Considering the slice of the lattice ${\mathcal{C}}$ containing the qubit *q*_1_ as the first slice (associated with repeater ${{\mathcal{R}}}_{1}$), we call the *j*-th slice the repeater ${{\mathcal{R}}}_{j}$.

Let *n* = *Θ*(*d*^2^*R*) be the number of vertices of the lattice ${\mathcal{C}}$. The protocol starts by preparing the 3D cluster state $W{\left\vert 0\right\rangle }^{\otimes n}$ of the lattice ${\mathcal{C}}$, where

1 $$W={H}^{\otimes n}\left(\prod _{\{u,v\}}C{Z}_{u,v}\right){H}^{\otimes n}$$

is a product of Hadamard gates and controlled-*Z* gates

$$\begin{array}{l}H=\frac{1}{\sqrt{2}}\left(\begin{array}{cc}1&1\\ 1&-1\end{array}\right)\quad \,\text{and}\,\quad C{Z}_{u,v}=\left\vert 0\right\rangle {\left\langle 0\right\vert }_{u}\otimes {\left(\begin{array}{cc}1&0\\ 0&1\end{array}\right)}_{v}\\\qquad+\,\left\vert 1\right\rangle {\left\langle 1\right\vert }_{u}\otimes {\left(\begin{array}{cc}1&0\\ 0&-1\end{array}\right)}_{v},\end{array}$$

and the product of controlled-*Z* gates *CZ*_*u*,*v*_ is taken over all edges {*u*, *v*} of the lattice ${\mathcal{C}}$.

**Fig. 4:**
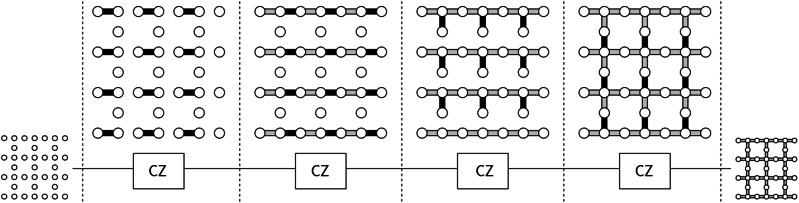
Sequence of quantum operations applied within each individual repeater (slice). Each ${\mathsf{CZ}}$ is a product of *CZ* gates on the black edges connecting the qubits (white balls). The gray edges of the graph at each layer represent the *CZ* gates applied in the previous steps. Each repeater runs this procedure in parallel. In the end, each repeater has a planar graph state.

**Fig. 5:**
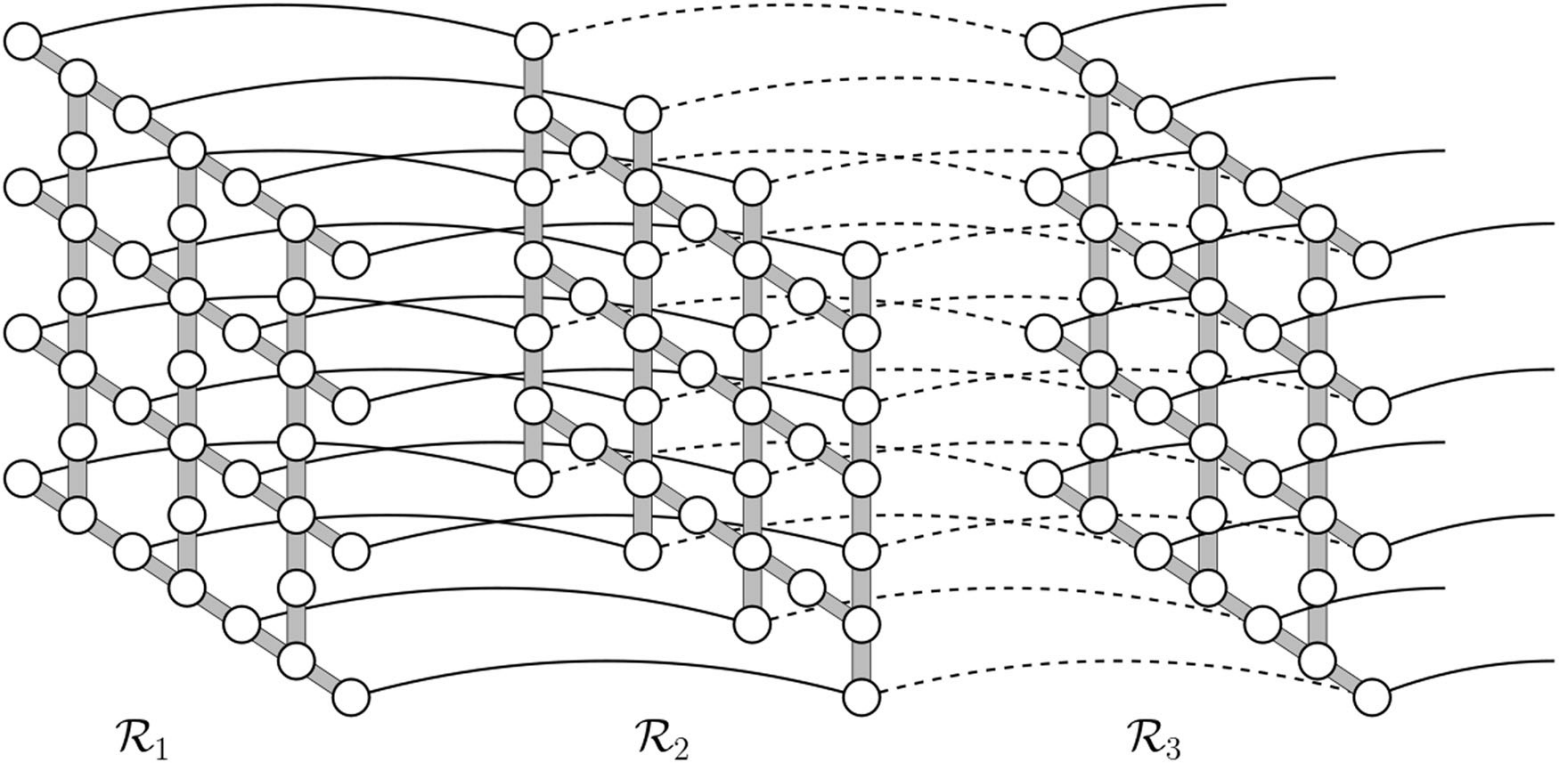
Quantum operations applied to nearest-neighboring repeaters consisting of two steps after the process illustrated in Fig. 4. The first step consists of two-qubit *CZ* operations between repeater ${{\mathcal{R}}}_{2j-1}$ and repeater ${{\mathcal{R}}}_{2j}$ (solid lines) for all *j* = 1, …, ⌊*R*/2⌋. The second step is of two-qubit *CZ* operations between repeater ${{\mathcal{R}}}_{2j}$ and repeater ${{\mathcal{R}}}_{2j+1}$ (dashed lines) for all *j* = 1, …, ⌊*R*/2⌋.

We first observe that the product ∏_{*u*, *v*}_*CZ*_*u*,*v*_ of controlled-*Z* gates in Eq. (1) can be written as a depth-6 quantum circuit as described in Figs. 4 and 5. Here the operations in Fig. 4 are “local” for each repeater, and are applied within each repeater ${{\mathcal{R}}}_{j}$ in parallel for *j* = 1, …, *R*. The operations illustrated in Fig. 5 couple neighboring odd-even pairs $({{\mathcal{R}}}_{2k-1},{{\mathcal{R}}}_{2k})$, *k* = 1, …, ⌊*R*/2⌋ of repeaters, and even-odd pairs $({{\mathcal{R}}}_{2k},{{\mathcal{R}}}_{2k+1})$, $k=1,\ldots ,\lfloor R\rfloor /2\rfloor$ of repeaters in two rounds.

In more detail, the first four layers consist of *CZ* gates on the edges connecting two qubits in the same node. The fifth layer consists of *CZ* gates acting on (2*j* − 1)-th and 2*j*-th repeaters for *j* = 1, …, ⌊*R*/2⌋ (see solid lines in Fig. 5), and the sixth layer consists of *CZ* gates acting on 2*j*-th and (2*j* + 1)-th repeaters for *j* = 1, …, ⌊*R*/2⌋ (see dashed lines in Fig. 5). In summary, we can write *W* as

$$W={H}^{\otimes n}{U}_{6}^{{\prime} }{U}_{5}{U}_{4}{U}_{3}{U}_{2}{U}_{1}^{{\prime} }{H}^{\otimes n}\,,$$

where all ${U}_{j}^{{\prime} }$ for *j* = 2, …, 5 together with ${U}_{1}^{{\prime} }$ and ${U}_{6}^{{\prime} }$ consist of *CZ* gates. Note that this depth-8 circuit is also illustrated in Fig. 2(b).

We can further merge the layers *H*^⊗*n*^ and ${U}_{1}^{{\prime} }$ into one layer ${U}_{1}={U}_{1}^{{\prime} }{H}^{\otimes n}$, which is a product of two-qubit gates *CZ*_*u*,*v*_*H*_*u*_ acting on different qubits. Analogously, we define the unitary ${U}_{6}={H}^{\otimes n}{U}_{6}^{{\prime} }$ as the layer obtained by merging two layers ${U}_{6}^{{\prime} }$ and *H*^⊗*n*^. Finally, we can write *W* as a depth-6 Clifford circuit

$$W={U}_{6}\cdots {U}_{1}\,,$$

where each *U*_*j*_ for *j* = 1, …, 6 consists of *CZ*_*u*,*v*_, *H*_*u*_*CZ*_*u*,*v*_ and *CZ*_*u*,*v*_*H*_*u*_ gates acting on pairwise disjoint qubits {*u*, *v*}.

To describe the remaining steps of the protocol, we consider the partition ${\mathcal{X}}\cup {\mathcal{Z}}\cup \{{q}_{1},{q}_{2}\}$ of these *n* qubits which is represented in Fig. 3. With the cluster state $W{\left\vert 0\right\rangle }^{\otimes n}$, we perform single-qubit measurements on the qubits except for two qubits *q*_1_ and *q*_2_. Here, each qubit in ${\mathcal{X}}$ and ${\mathcal{Z}}$ is measured in the Hadamard- and computational basis, respectively.

We can construct functions $\alpha ,\beta :{\{0,1\}}^{{\mathcal{X}}\cup {\mathcal{Z}}}\to \{0,1\}$ such that given the measurement results $s\in {\{0,1\}}^{{\mathcal{X}}\cup {\mathcal{Z}}}$, the post-measurement state on *q*_1_ and *q*_2_ is ${Z}_{{q}_{2}}^{\alpha (s)}{X}_{{q}_{2}}^{\beta (s)}{\left\vert \Phi \right\rangle }_{{q}_{1},{q}_{2}}$, if all operations are perfect. Therefore, given the two bits *α*(*s*) and *β*(*s*), one can apply the Pauli “correction” operator *Z*^*α*(*s*)^*X*^*β*(*s*)^ to qubit *q*_2_ to obtain the desired state ${\left\vert \Phi \right\rangle }_{{q}_{1},{q}_{2}}$. Note that for many applications which can be written as Clifford circuits, it is sufficient to compute the two bits *α*(*s*) and *β*(*s*) to correct the Pauli operator in “software”, i.e., one can commute the Pauli correction *Z*^*α*(*s*)^*X*^*β*(*s*)^ through a stabilizer circuit without the need for physically applying a correction.

Before we move on to the precise description of the components of the protocol, we observe that the protocol satisfies the low-latency and geometric locality requirements discussed in the Introduction: The whole process can be written in a constant-depth quantum circuit with Pauli gates controlled by classical bits obtained by computing the bits *α*(*s*) and *β*(*s*) (see Fig. 2(b)). Moreover, the involved qubits are arranged in a slab-like 3D lattice, as shown in Fig. 3, and operations are between neighboring qubits only. A precise description of the lattice ${\mathcal{C}}[d\times d\times R]$ as well as the validity of the protocol is deferred to Sections 4 and 5 of the Supplementary Information.

Here we state the definition of *α* and *β* and discuss when the protocol fails to output the correct Bell basis state in the scenario where the protocol runs with the noisy cluster state $EW{\left\vert 0\right\rangle }^{\otimes n}$ corrupted by some *n*-qubit random Pauli *E* and all measurements are perfect. (We also discuss more general circuit-level noise below.)

First, note that since all qubits in ${\mathcal{X}}$ and ${\mathcal{Z}}$ are measured in Hadamard basis and computational basis, respectively, we may assume that the random Pauli *E* is of the form

$$E={E}_{{q}_{1},{q}_{2}}{E}_{{\mathcal{X}}}{E}_{{\mathcal{Z}}}$$

where ${E}_{{q}_{1},{q}_{2}}$ is a random Pauli supported on the qubits *q*_1_ and *q*_2_, ${E}_{{\mathcal{X}}}$ is an *X*-type random Pauli supported on ${\mathcal{X}}$, and ${E}_{{\mathcal{Z}}}$ is a *Z*-type random Pauli supported on ${\mathcal{Z}}$. In particular, from now on, we think of ${E}_{{\mathcal{X}}}$ and ${E}_{{\mathcal{Z}}}$ respectively as a (random) subset of ${\mathcal{X}}$ and ${\mathcal{Z}}$.

**Fig. 6:**
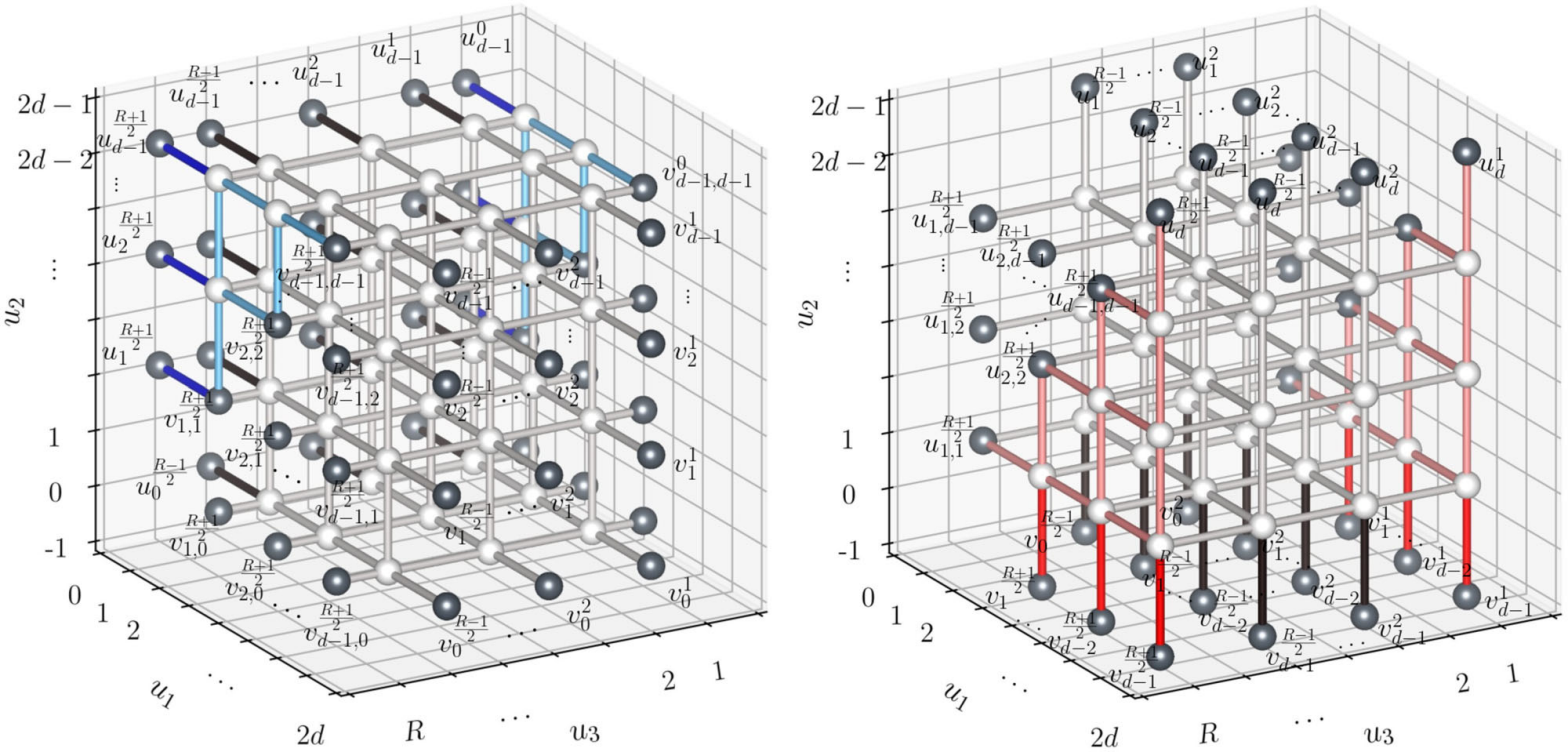
The decoding graph ${{\mathsf{T}}}_{{\rm{cl}},{\rm{dec}}}$ as well as the dual decoding graph ${{\mathsf{T}}}_{{\rm{cl}},{{\rm{dec}}}^{* }}$. External vertices (black spheres) and internal vertices (white spheres) are illustrated. The external vertices incident to the edges in ${{\mathcal{L}}}_{{\rm{cl}},X}$ and ${{\mathcal{L}}}_{{\rm{cl}},Z}^{* }$ form the membranes *M*_1_ and ${M}_{{1}^{* }}$. Labels on the external vertices are used in Supplementary Information for the analysis on the failure probability of the protocol.

The main subroutine contained in the (algorithmic) definition of *α* and *β* is solving minimum matching problems^39^ on the (primal) and dual decoding graphs

$${{\mathsf{T}}}_{{\rm{cl}},{\rm{dec}}}=({{\mathsf{V}}}_{{\rm{cl}},{\rm{dec}}},{{\mathsf{E}}}_{{\rm{cl}},{\rm{dec}}})\quad \,\text{and}\,\quad {{\mathsf{T}}}_{{\rm{cl}},{{\rm{dec}}}^{* }}=({{\mathsf{V}}}_{{\rm{cl}},{{\rm{dec}}}^{* }},{{\mathsf{E}}}_{{\rm{cl}},{{\rm{dec}}}^{* }})$$

illustrated in Fig. 6. The full definitions of the decoding graphs are deferred to Section 4 of the Supplementary Information, but here we state all properties of these graphs to give the definition of *α* and *β*.

1. Each qubit in ${\mathcal{X}}\cup {\mathcal{Z}}$ corresponds to one of the edges of ${{\mathsf{T}}}_{{\rm{cl}},{\rm{dec}}}$ or ${{\mathsf{T}}}_{{\rm{cl}},{{\rm{dec}}}^{* }}$. With this correspondence, the subset ${E}_{{\mathcal{X}}}\cup {E}_{{\mathcal{Z}}}\subseteq {\mathcal{X}}\cup {\mathcal{Z}}$ can be represented as a subset of the edges of the decoding graphs. Let us denote these subsets of the edges by
  $$\begin{array}{rcl}{E}_{{\rm{dec}}}&=&({E}_{{\mathcal{X}}}\cup {E}_{{\mathcal{Z}}})\cap {{\mathsf{E}}}_{{\rm{cl}},{\rm{dec}}}\\ {E}_{{{\rm{dec}}}^{* }}&=&({E}_{{\mathcal{X}}}\cup {E}_{{\mathcal{Z}}})\cap {{\mathsf{E}}}_{{\rm{cl}},{{\rm{dec}}}^{* }}.\end{array}$$
2. Each vertex of the decoding graphs is either *internal* or *external*. The set of external vertices of a decoding graph ${{\mathsf{T}}}_{{\rm{cl}},{\rm{dec}}}$ is partitioned into two “membranes” *M*_1_ and *M*_2_. We collect all edges connecting the vertices in *M*_1_ with internal vertices and let ${{\mathcal{L}}}_{{\rm{cl}},X}$ denote the set of these edges. We analogously define the membranes ${M}_{{1}^{* }}$ and ${M}_{{2}^{* }}$ of the other decoding graph ${{\mathsf{T}}}_{{\rm{cl}},{{\rm{dec}}}^{* }}$ and the set of edges ${{\mathcal{L}}}_{{\rm{cl}},Z}^{* }$.

Given the measurement result $s\in {\{0,1\}}^{{\mathcal{X}}\cup {\mathcal{Z}}}$, the bits *α*(*s*) and *β*(*s*) are computed as follows:

1. Considering *s* as a subset of the set of edges ${{\mathsf{E}}}_{{\rm{cl}},{\rm{dec}}}\cup {{\mathsf{E}}}_{{\rm{cl}},{{\rm{dec}}}^{* }}$, partition *s* into two subsets
  $$s={s}_{{\rm{dec}}}\cup {s}_{{{\rm{dec}}}^{* }}\quad \,\text{where}\,\quad {s}_{{\rm{dec}}}=s\cap {{\mathsf{E}}}_{{\rm{cl}},{\rm{dec}}}\quad \,\text{and}\,\quad {s}_{{{\rm{dec}}}^{* }}=s\cap {{\mathsf{E}}}_{{\rm{cl}},{{\rm{dec}}}^{* }}.$$
2. Compute the boundaries ${\partial }_{{{\mathsf{T}}}_{{\rm{cl}},{\rm{dec}}}}({s}_{{\rm{dec}}})$ and ${\partial }_{{{\mathsf{T}}}_{{\rm{cl}},{{\rm{dec}}}^{* }}}({s}_{{\rm{dec}}})$ of *s*_dec_ and ${s}_{{{\rm{dec}}}^{* }}$ in the decoding graphs ${{\mathsf{T}}}_{{\rm{cl}},{\rm{dec}}}$ and ${{\mathsf{T}}}_{{\rm{cl}},{{\rm{dec}}}^{* }}$. Here, the boundary ${\partial }_{{\mathsf{T}}}({{\mathsf{E}}}^{{\prime} })$ of a subset ${{\mathsf{E}}}^{{\prime} }\subseteq {\mathsf{E}}$ of edges in a decoding graph ${\mathsf{T}}$ is defined as the set of internal vertices of ${{\mathsf{T}}}_{{\rm{cl}},{\rm{dec}}}$ incident to odd number of edges in ${{\mathsf{E}}}^{{\prime} }$.
3. Compute minimal matchings
  $$\begin{array}{rcl}m&=&{{\mathsf{MinMatch}}}_{{{\mathsf{T}}}_{{\rm{cl}},{\rm{dec}}}}({\partial }_{{{\mathsf{T}}}_{{\rm{cl}},{\rm{dec}}}}({s}_{{\rm{dec}}}))\\ {m}^{* }&=&{{\mathsf{MinMatch}}}_{{{\mathsf{T}}}_{{\rm{cl}},{{\rm{dec}}}^{* }}}({\partial }_{{{\mathsf{T}}}_{{\rm{cl}},{{\rm{dec}}}^{* }}}({s}_{{{\rm{dec}}}^{* }}))\end{array}$$
  of the internal vertices ${\partial }_{{{\mathsf{T}}}_{{\rm{cl}},{\rm{dec}}}}({s}_{{\rm{dec}}})$ and ${\partial }_{{{\mathsf{T}}}_{{\rm{cl}},{{\rm{dec}}}^{* }}}({s}_{{\rm{dec}}}^{* })$. Here, a minimal matching ${{\mathsf{MinMatch}}}_{{\mathsf{T}}}({{\mathsf{V}}}^{{\prime} })$ of a subset ${{\mathsf{V}}}^{{\prime} }$ of internal vertices in a decoding graph ${\mathsf{T}}$ is a minimal subset of edges of ${\mathsf{T}}$ whose boundary is ${{\mathsf{V}}}^{{\prime} }$.
4. Finally, compute the bits
  $$\begin{array}{rcl}\alpha (s)&=&\left\langle \left\langle {s}_{{\rm{dec}}}\oplus m,{{\mathcal{L}}}_{{\rm{cl}},X}\right\rangle \right\rangle \\ \beta (s)&=&\left\langle \left\langle {s}_{{{\rm{dec}}}^{* }}\oplus {m}^{* },{{\mathcal{L}}}_{{\rm{cl}},Z}^{* }\right\rangle \right\rangle .\end{array}$$
  Here, for two sets *A* and *B*, we denote *A* ⊕ *B* the symmetric difference of *A* and *B*, or equivalently the bit-wise summation over modulo 2 if all sets are considered as bitstrings. The notation $\left\langle \left\langle A,B\right\rangle \right\rangle$ denotes the parity of the number of elements in the intersection *A* ∩ *B*, or equivalently the usual inner product on the bitstring defined as the parity of the sum of the bits obtained by bit-wise multiplication of *A* and *B* which are considered as bitstrings.

A key fact here is that the two subsets *s*_dec_ and *E*_dec_ have the same boundaries in the decoding graph ${{\mathsf{T}}}_{{\rm{cl}},{\rm{dec}}}$, i.e.,

$${\partial }_{{{\mathsf{T}}}_{{\rm{cl}},{\rm{dec}}}}({s}_{{\rm{dec}}})={\partial }_{{{\mathsf{T}}}_{{\rm{cl}},{\rm{dec}}}}({E}_{{\rm{dec}}})\,.$$

Analogously, we have

$${\partial }_{{{\mathsf{T}}}_{{\rm{cl}},{{\rm{dec}}}^{* }}}({s}_{{{\rm{dec}}}^{* }})={\partial }_{{{\mathsf{T}}}_{{\rm{cl}},{{\rm{dec}}}^{* }}}({E}_{{{\rm{dec}}}^{* }})$$

for the subsets ${s}_{{{\rm{dec}}}^{* }}$ and ${E}_{{{\rm{dec}}}^{* }}$ of the edges of the decoding graph ${{\mathsf{T}}}_{{\rm{cl}},{{\rm{dec}}}^{* }}$ In particular, the boundaries of the errors *E*_dec_ and ${E}_{{{\rm{dec}}}^{* }}$ can be computed from the measurement outcome *s*. The main role of the function *α* is finding the estimate

$${\hat{E}}_{{\rm{dec}}}(s):=m={{\mathsf{MinMatch}}}_{{{\mathsf{T}}}_{{\rm{cl}},{\rm{dec}}}}({\partial }_{{{\mathsf{T}}}_{{\rm{cl}},{\rm{dec}}}}({s}_{{\rm{dec}}}))$$

of *E*_dec_ with the information about its boundary in the decoding graph by solving a minimum matching problem^39^ on the decoding graph ${{\mathsf{T}}}_{{\rm{cl}},{\rm{dec}}}$. Analogously, a subroutine of *β* computes a minimal matching

$${\hat{E}}_{{{\rm{dec}}}^{* }}(s):={m}^{* }={{\mathsf{MinMatch}}}_{{{\mathsf{T}}}_{{\rm{cl}},{{\rm{dec}}}^{* }}}({\partial }_{{{\mathsf{T}}}_{{\rm{cl}},{{\rm{dec}}}^{* }}}({s}_{{{\rm{dec}}}^{* }}))$$

of the boundary of ${E}_{{{\rm{dec}}}^{* }}$ associated with the dual decoding graph ${{\mathsf{T}}}_{{\rm{cl}},{{\rm{dec}}}^{* }}$. With Edmond’s algorithm^39–42^, if the repeater *R*, which contains *q*_2_, receives all measurement results from all other *R* − 1 repeaters, then it can evaluate the estimates ${\hat{E}}_{{\rm{dec}}}(s)$ and ${\hat{E}}_{{{\rm{dec}}}^{* }}(s)$ in a time *O*(*d*^6^ ⋅ *R*^3^). (The “flow” of the classical information, which is the measurement results, is illustrated as double line segments in Fig. 2).

**Fig. 7:**
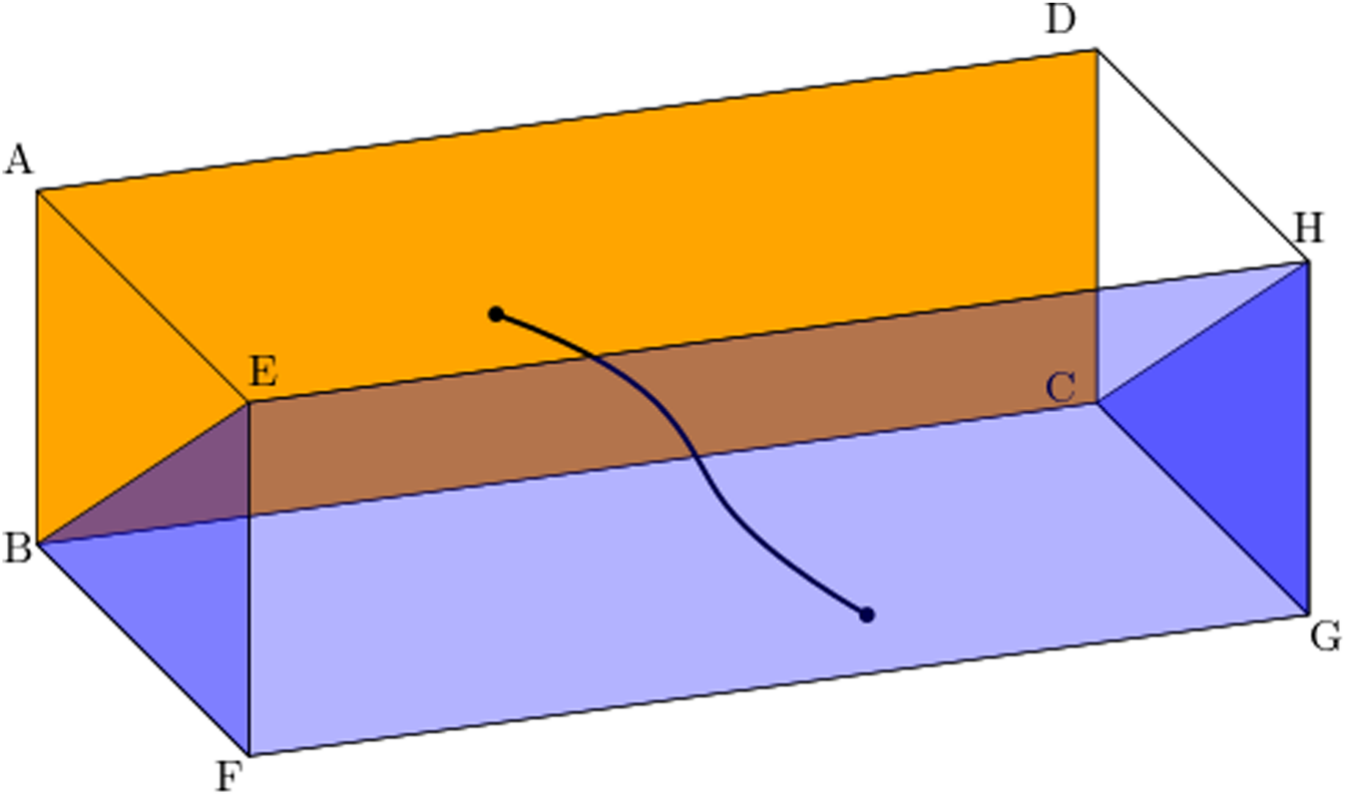
A schematic diagram of the decoding graph ${{\mathsf{T}}}_{{\rm{cl}},{\rm{dec}}}$ illustrating the case where the subset of edges ${E}_{{\rm{dec}}}\oplus {\hat{E}}_{{\rm{dec}}}(s)$ contains a subset of edges forming a path in the graph ${{\mathsf{T}}}_{{\rm{cl}},{\rm{dec}}}$ (a black curve) which connects two membranes *M*_1_ = ABCD and *M*_2_ = BFE ∪ EFGH ∪ CGH. These membranes are represented in orange (*M*_1_) and blue (*M*_2_).

A necessary condition for the failure of the protocol, i.e., the case where the output state is not the desired Bell state $\left\vert \Phi \right\rangle$, is that the Pauli ${E}_{{q}_{1},{q}_{2}}$ on the qubits *q*_1_ and *q*_2_ is non-trivial. Geometrically, this implies that at least one of the subsets ${E}_{{\rm{dec}}}\oplus {\hat{E}}_{{\rm{dec}}}(s)\subseteq {{\mathsf{E}}}_{{\rm{cl}},{\rm{dec}}}$ or ${E}_{{{\rm{dec}}}^{* }}\oplus {\hat{E}}_{{{\rm{dec}}}^{* }}(s)\subseteq {{\mathsf{E}}}_{{\rm{cl}},{{\rm{dec}}}^{* }}$ of the edges connects two membranes *M*_1_ and *M*_2_ or ${M}_{{1}^{* }}$ and ${M}_{{2}^{* }}$ of the decoding graphs at the opposite sides, consisting of external vertices, see Fig. 7. The proof of the necessity of this condition for failure of the protocol is deferred to Section 4 of the Supplementary Information.

### A threshold theorem for low-latency long-range entanglement generation

We prove that the entanglement generation protocol using a 3D cluster state is robust under local stochastic noise of strength below a threshold, even with a communication distance *R* ≫ *d*. The formal definition of local stochastic errors is as follows.

#### Definition 1

(Local stochastic errors). Let [*n*]: = {1, …, *n*} denote the set of *n* qubits. A random *n*-qubit Pauli *E* is local stochastic with strength *p* ∈ [0, 1] if

$$\Pr [S\subseteq {\rm{supp}}(E)]\le {p}^{| S| }\quad \,{\rm{for}}\, {\rm{all}}\,\quad S\subseteq [n],$$

where supp(*E*) is the set of all qubits where *E* acts non-trivially. We write $E \sim {\mathcal{N}}(p)$ if *E* is local stochastic with strength *p*.

We first state our result on fault-tolerance of the protocolin the case where the protocol runs with the noisy cluster state $EW{\left\vert 0\right\rangle }^{\otimes n}$ where $E \sim {\mathcal{N}}(p)$ is a random Pauli which is local stochastic with strength *p*, and there is no other source of error.

#### Theorem 2

(Long-range entanglement generation). Consider a cluster state $W{\left\vert 0\right\rangle }^{\otimes n}$ associated with the lattice ${\mathcal{C}}[d\times d\times R]$ where *d* ≥ 3 is arbitrary. Suppose the measurement pattern in Fig. 3 is applied to a corrupted cluster state $EW{\left\vert 0\right\rangle }^{\otimes n}$, where $E \sim {\mathcal{N}}(p)$ is a local stochastic error. Then there are two efficiently computable functions *α*, *β* taking the collection *s* of measurement outcomes to bits such that the post-measurement state on qubits *q*_1_, *q*_2_ is the state ${Z}_{{q}_{2}}^{\alpha (s)}{X}_{{q}_{2}}^{\beta (s)}\Phi$ with probability at least 1 − 5006*p*, for any

$$0\, < \,p\,\le \,{p}_{0}:=\frac{1}{5006}\approx 2\times 1{0}^{-4}\qquad \,{\rm{and}}\, {\rm{any}}\,\qquad R\le \frac{1}{\sqrt{d}}{\left(\frac{1}{10\sqrt{p}}\right)}^{d-2}.$$

Here, we only give a sketch of the proof of Theorem 2 presented in Section 5 of the Supplementary Information. In the proof of Theorem 2, we show that for a random Pauli $E \sim {\mathcal{N}}(p)$, the subset ${E}_{{\rm{dec}}}\oplus {\hat{E}}_{{\rm{dec}}}(s)$ of the edges contains a path connecting the two membranes of the decoding graph ${{\mathsf{T}}}_{{\rm{cl}},{\rm{dec}}}$ at the opposite sides with probability at most *O*(*p*). We also show a similar result for the subset ${E}_{{{\rm{dec}}}^{* }}\oplus {\hat{E}}_{{{\rm{dec}}}^{* }}(s)$ of ${{\mathsf{E}}}_{{\rm{cl}},{\rm{dec}}}$. The upper bound on the failure probability is obtained by applying the union bound to these events.

We also show that fault-tolerance of the protocol with this simplified noise model (with noise only on the cluster state $W{\left\vert 0\right\rangle }^{\otimes n}$, i.e., Theorem 2 implies that the protocol is robust under circuit-level local stochastic noise of strength below some constant threshold where Pauli error occurs before and after every layer of the state preparation, unitary operation, and single-qubit measurements, see the Methods section.

#### Optimality: A converse to the threshold theorem

Considering each (2*d* − 1) × (2*d* − 1)-slice of the lattice ${\mathcal{C}}[d\times d\times R]$ as a repeater, we have *m* = *O*(*d*^2^) qubits per repeater. Theorem 2 thus implies that below the threshold error strength *p*_0_,

2 $$m=O({\log }^{2}R)$$

qubits per repeater are sufficient to establish constant-fidelity over distance *R*. Thus, the cluster-state-based protocol satisfies the properties discussed in the introduction.

In this subsection, we study to which extent the protocol provided by Theorem 2 is optimal among protocols with low latency. Specifically, we ask if the relationship between the number *m* of qubits at each site and the communication distance *R* can be better than polylogarithmic (cf. (2)). We find that this is not the case, i.e., the scaling of our protocol is essentially optimal.

##### Theorem 3

(Converse for low latency schemes). Let *π* be an entanglement generation of low-latency over communication distance *R* which uses *m* qubits per repeater. If its output state achieves fidelity at least $\frac{1}{2}(1+{e}^{-1})\approx 0.684$ under arbitrary local stochastic noise of strength *p* below a constant threshold *p*_0_ > 0, then we must have $m=\Omega (\log R)$.

The proof of Theorem 3 replaces the analysis of a low-latency protocol *π* by that of an entanglement-assisted protocol ${\pi }^{{\prime} }$, and follows from the fact that for any *p* ∈ (0, 1), the qubit depolarizing channel ${{\mathcal{E}}}_{p}(\rho )=(1-p)\rho +p{\rm{tr}}(\rho )\frac{I}{2}$ is a convex combination of an entanglement-breaking channel^43^ and the identity channel (see Supplementary Information Section 6 for details). It implies that local stochastic noise with any constant noise strength prevents entanglement generation over distances greater than exponential in the number of qubits at each site. Comparing the lower bound from the converse theorem with the number of qubits per site from our protocol (2), we conclude that the polylogarithmic scaling on the number *m* of Theorem 2 can at best be improved to a logarithmic scaling in *R*.

Closing the log-factor gap between the converse Theorem 3 and achievability Theorem 2 appears to require a new approach, however: We can show that the analysis of Theorem 2 leading to the scaling (2) is tight. In fact, the scaling is necessary for any procedure which is based on the cluster state and uses the same syndrome information using functions *α* and *β* as in our protocol: We show the lower bound

$$m=\Omega ({\log }^{2}R)$$

for any such protocol. This result is obtained by a detailed analysis of the associated decoding problem, see Supplementary Information Section 6. It applies, in particular, to natural variants of our scheme that are obtained, e.g., by replacing Edmond’s minimal matching algorithm by other procedures, including heuristic algorithms. This indicates that improving over our protocol will require a different resource state or measurement pattern.

#### Understanding the protocol with surface code single-shot decoding

We give a modular way of understanding the protocol, giving a high-level overview focused on the two main building blocks:

(I) **Generating a surface-code-encoded Bell pair**: The generation of a cluster state on an elongated lattice ${\mathcal{C}}[d\times d\times R]$ and subsequent single-qubit measurement of “bulk” qubits not belonging to the two boundaries with third coordinate equal to *u*_3_ = 1 and *u*_3_ = *R*, respectively. Up to local unitaries, this part of the procedure is identical to the original scheme of^36^. This generates a surface-code encoded Bell pair up to a (computable) Pauli correction and residual local stochastic noise [^37^, Result 3].
(II) **Single-shot decoding of the surface code**: A single-shot decoding procedure for transferring surface-code encoded quantum information onto a single physical qubit. We note that the same idea was used in^44^ for logical *T*-basis measurement. We show that this procedure is resilient to local stochastic noise: There is a threshold ${p}_{0}^{{\rm{dec}}}$ such that if the initial encoded state $\overline{\Psi }$ is corrupted by a local stochastic noise with strength $p\le {p}_{0}^{{\rm{dec}}}$, then the fidelity of the resulting single-qubit state with the logical state $\overline{\Psi }$ is linearly close to 1 as a function of the noise strength *p*. We refer to Supplementary Information Section 1 and 3 for details.

From a conceptual point of view, it is clear that combining (I) with (II) results in a procedure for generating long-range entanglement. However, our analysis of the resulting fidelity is not simply a combination of the analytical results stated above for each building block (I) and (II). This is because a threshold result derived in this way does not yield parameters of practical relevance. Instead, we establish a significantly tighter threshold estimate by a direct analysis of the achieved fidelity for the entire (combined) process.

## Discussion

In this work, we give a complete analysis of the robustness of the distance-*R* entanglement generating protocol based on the 3D cluster state of size (2*d* − 1) × (2*d* − 1) × *R* with $d=O(\log R)$ under circuit-level local stochastic noise. Our main result is a fault-tolerance threshold theorem giving a bound on the tolerable noise strength.

We also show that our protocol based on the 3D cluster state gives rise to a low-latency protocol for long-range entanglement generation by a set of *R* equidistant repeaters on a line. We prove that fault-tolerant generation of one Bell pair between two end nodes by any low-latency protocol requires at least $m=\Omega (\log R)$ qubits per node. Our construction uses $m=O({\log }^{2}R)$ quibts at each repeater, which we show is optimal for the 3D cluster-state approach. We note that it is still an open problem whether there exists a low-latency protocol which saturates the lower bound $m=\Omega (\log R)$.

## Methods

In this section, we explain how to reduce the problem of showing fault-tolerance of low-latency protocols with circuit-level noise to the setting of a noisy cluster state $EW{\left\vert 0\right\rangle }^{\otimes n}$ (and noise-free operations otherwise). We note that this reduction is same as that of ref. ^37^, and it is ubiquitous in the error correction literature, see e.g., refs. ^15,45^.

Recall that the protocol we proposed (without the correction operator) is essentially preparing the state $W{\left\vert 0\right\rangle }^{\otimes n}$ followed by the single-qubit measurements, where *W* is a Clifford unitary

$$W={U}_{\Delta }\cdots {U}_{1}$$

of depth *Δ* = 6, where each *U*_*j*_ is a tensor product of single- or two-qubit Clifford unitaries whose supports are disjoint.

A noisy implementation of the whole protocol with noise strength *p* ∈ [0, 1] can be described as the protocol performing the perfect single-qubit measurements on a noisy input state

3 $${E}_{\Delta +1}{E}_{\Delta }{U}_{\Delta }{E}_{\Delta -1}\cdots {E}_{1}{U}_{1}{E}_{0}{\left\vert 0\right\rangle }^{\otimes n}$$

with *Δ* = 6. Here, all ${E}_{j} \sim {\mathcal{N}}(p)$ are local stochastic errors of strength *p* on all qubits. In particular, *E*_0_ denotes the Pauli error caused by noisy state preparation, and *E*_*Δ*+1_ denotes the Pauli error which is introduced to describe the measurement error.

Proving the robustness of the protocol with a noisy state of the form (3) seems to be complicated. However, we can sidestep this problem with the following observation: Since all *U*_*j*_ are Clifford unitaries, we can “commute” all Pauli errors *E*_*j*_ to the left. For example, there exists a Pauli ${E}_{0}^{{\prime} }$ such that ${E}_{0}^{{\prime} }{U}_{1}={U}_{1}{E}_{0}$, i.e., ${E}_{0}^{{\prime} }={U}_{1}{E}_{0}{U}_{1}^{\dagger }$ is obtained by conjugating *E*_0_ with the Clifford unitary *U*_1_. Then, consider the Pauli ${E}_{1}{E}_{0}^{{\prime} }$ and apply the similar technique with the Clifford unitary *E*_2_. By repeating this procedure, we end up with some Pauli *E* such that the noisy state (3) is equal to

4 $$EW{\left\vert 0\right\rangle }^{\otimes n}\,.$$

More importantly, $E \sim {\mathcal{N}}({p}^{{\prime} })$ is also a local stochastic error of strength ${p}^{{\prime} }$ with ${p}^{{\prime} }=4{p}^{{4}^{-(\Delta +1)}}$. This fact follows from [^37^, Lemma 12]: The main idea here is using the commuting technique described above with the following update rules of the noise strength: If $E \sim {\mathcal{N}}(p)$ and ${E}^{{\prime} } \sim {\mathcal{N}}(q)$ are Paulis and *U* is a depth-1 Clifford unitary consisting of one- and two-qubit gates, then $E\cdot {E}^{{\prime} } \sim {\mathcal{N}}(2\max \{\sqrt{p},\sqrt{q}\})$ and $UE{U}^{\dagger } \sim {\mathcal{N}}(\sqrt{2p})$, see also [^37^,Lemma 11] for the proof of the update rules. Therefore, if we show that the the protocol is robust for a noisy initial cluster state $EW{\left\vert 0\right\rangle }^{\otimes n}$ (see Eq. (4)), where the only error during the whole process is the random Pauli $E \sim {\mathcal{N}}({p}^{{\prime} })$ with ${p}^{{\prime} }\le {p}_{0}^{{\prime} }$, then we can say that the protocol is robust with the circuit-level noise model with random Paulis ${E}_{j} \sim {\mathcal{N}}(p)$ in Eq. (3) for all *p* ≤ *p*_0_ with some constant threshold *p*_0_ > 0.

## Supplementary information

Supplementary Information

## Data Availability

Data sharing is not applicable to this article as no datasets were generated or analysed during the current study.
