# Supplementary material for: Long-range data transmission in a fault-tolerant quantum bus architecture
Source: npj Quantum Inf. 2024 Dec 26;10(1):132. doi: 10.1038/s41534-024-00928-4 (PMC11671373; doi:10.1038/s41534-024-00928-4)
Supplement: Supplementary file 1 — Supplementary Information [file 41534_2024_928_MOESM1_ESM.pdf]

Long-range data transmission in a fault-tolerant quantum bus architecture: Supplementary Information

Shin Ho Choe[1,2] and Robert König[1,2]

[1]Department of Mathematics, School of Computation, Information and Technology, Technical University of Munich, Garching, Germany
[2]Munich Center for Quantum Science and Technology (MCQST), Munich, Germany

August 12, 2024

We give detailed proofs of the main results discussed in this article. In Section 1, we introduce our single-shot decoding protocol for the surface code accompanied with background material. In Section 2, we provide a combinatorial framework for analyzing matching problems arising in the analysis of both our surface code decoding protocol and the entanglement generation protocol introduced in Section 4. Using the framework, we prove in Section 3 that our surface code decoding protocol is robust against local stochastic noise.

Then in Section 4, we introduce our long-range entanglement generation protocol. We prove in Section 5 that this protocol is resilient against local stochastic noise of strength below a certain threshold: A lower bound on the achievable distance of entanglement generation is presented in terms of the noise strength and the number of qubits. Finally, in Section 6, we present a converse result for low latency entanglement generation schemes.

1 Single-shot surface code decoding

In this section, we define our single-shot decoding protocol for the surface code: We introduce all relevant definitions to apply this protocol in practice. We defer the analysis of the resilience of this protocol against local stochastic noise to subsequent sections: In Section 2, we will first provide general bounds on certain matching problems on graphs. These will subsequently be applied in Section 3 to establish a fault-tolerance threshold theorem for the single-shot decoding protocol introduced here.

1.1 Definition of surface code

We consider a distance-d-surface code with a total of $n = 2d^2-2d+1$ qubits associated with d^2 horizontal edges and $(d-1)^2$ vertical edges as illustrated in Fig. 1. The code has smooth boundaries at the top and bottom, and rough boundaries on the left and right. In more detail, consider a square lattice in $\mathbb{R}^2$ consisting of sites

$$\widetilde{\mathcal{C}} = \left\{(u_1, u_2) \in \mathbb{Z}^2 \mid 0 \leq u_1 \leq 2d \text{ and } 0 \leq u_2 \leq 2d-2\right\} .$$

We place qubits at sites $(u_1, u_2) \in \widetilde{\mathcal{C}}$ with the property that one coordinate u_j is even, whereas the other coordinate u_k is odd, i.e., qubits are located at the sites

$$\mathcal{C} = \widetilde{\mathcal{C}} \setminus \{(o,o),(e,e)\} = \{(o,e)\} \cup \{(e,o)\} \subset \widetilde{\mathcal{C}}.$$

Here and below we write e (respectively o) for an even (respectively odd) integer and use the convention that e.g., $\{(o,o)\} \subset \widetilde{\mathcal{C}}$ is the subset of all pairs $(u_1, u_2) \in \widetilde{\mathcal{C}}$ with both u_1 and u_2 even. Let

$$d(u,v) := \sum_{j=1}^{2} |u_j - v_j|$$

denote Manhattan distance between u and v. Given a site $u \in \widetilde{\mathcal{C}}$, the set of nearest neighbors of u is defined as

$$\text{neigh}(u) = \{v \in \mathcal{C} \mid d(u,v) = 1\}$$

i.e., the nearest neighbors of u are associated with qubits at Manhattan distance 1 from u.

The surface code graph $T_{sc} = (\mathsf{V}_{\text{sc}}, \mathsf{E}_{\text{sc}})$ is defined as

$$\begin{aligned} \mathsf{V}_{\text{sc}} &= \{(e,e)\} \subset \widetilde{\mathcal{C}} \\ \mathsf{E}_{\text{sc}} &= \{\{u,v\} \in \mathsf{V}_{\text{sc}} \times \mathsf{V}_{\text{sc}} \mid (u_1, v_1) \not\in \{(0,0), (2d, 2d)\}, d(u,v) = 2\} \ . \end{aligned}$$

In other words, edges of T_{sc} are associated with pairs (u,v) separated by a distance $d(u,v) = 2$, with the exception of pairs (u,v) that lie on the same vertical at $u_1 = v_1 = 0$ or $u_1 = v_1 = 2d$. The corresponding vertices

$$\mathsf{V}'_{\text{sc}} = \{(0,e)\} \cup \{(2d,e)\} \subset \mathsf{V}_{\text{sc}}$$

are degree-1 vertices in T_{sc}, i.e., endpoints of "dangling" edges at one of the "rough" boundaries of T_{sc}: they are connected only to their right respectively left nearest neighbor. We can therefore express the set of edges of T_{sc} succintly as

$$\mathsf{E}_{\text{sc}} = \{\{u,v\} \in \mathsf{V}_{\text{sc}} \times \mathsf{V}_{\text{sc}} \mid d(u,v) = 2 \text{ and } (u,v) \not\in \mathsf{V}'_{\text{sc}} \times \mathsf{V}'_{\text{sc}}\} \ .$$

In the following, it will be convenient to label an edge $\{u,v\} \in \mathsf{E}_{\text{sc}}$ by its midpoint $(u+v)/2 \in \mathcal{C}$.

We will extensively use a "dual graph" $\mathsf{T}_{\text{sc}^*} = (\mathsf{V}_{\text{sc}^*}, \mathsf{E}_{\text{sc}^*})$. To define this, let us first consider the set

$$\widetilde{\mathcal{C}}^* = \left\{(u_1, u_2) \in \mathbb{Z}^2 \mid 1 \leq u_1 \leq 2d-1 \text{ and } -1 \leq u_2 \leq 2d-1\right\}$$

of dual sites. We note that the location of qubits can equivalently be specified as

$$\mathcal{C}^* = \{(o,e)\} \cup \{(e,o)\} \subset \widetilde{\mathcal{C}}^* \ .$$

i.e., $\mathcal{C} = \mathcal{C}^*$. The dual surface code graph $\mathsf{T}_{\text{sc}^*} = (\mathsf{V}_{\text{sc}^*}, \mathsf{E}_{\text{sc}^*})$ is defined by

$$\begin{aligned} \mathsf{V}_{\text{sc}^*} &= \{(o,o)\} \subset \widetilde{\mathcal{C}}_* \\ \mathsf{E}_{\text{sc}^*} &= \{\{u,v\} \in \mathsf{V}_{\text{sc}^*} \times \mathsf{V}_{\text{sc}^*} \mid (u_2, v_2) \not\in \{(-1,-1), (2d-1, 2d-1)\} \text{ and } d(u,v) = 2\} \ . \end{aligned}$$

Here degree-1-vertices, i.e., those belonging to

$$\mathsf{V}'_{\text{sc}^*} = \{(o,-1)\} \cup \{(o, 2d-1)\} \subset \mathsf{V}_{\text{sc}^*} \ ,$$

are located at the top- and bottom "rough" boundaries. Again, we sometimes refer to an edge $\{u,v\} \in \mathsf{E}_{\text{sc}^*}$ by its midpoint $(u+v)/2$.

The surface code associated with the surface code graphs $\mathsf{T}_{\text{sc}}, \mathsf{T}_{\text{sc}^*}$ is defined as follow: For every vertex $u \in \mathsf{V}_{\text{sc}}$ with degree $\deg_{\mathsf{T}_{\text{sc}}}(u) \geq 3$, i.e., every vertex $u \in \mathsf{V}_{\text{sc}} \setminus \mathsf{V}'_{\text{sc}}$ which is not the endpoint of a dangling edge u, there is a stabilizer generator $A_u := \prod_{v \in \text{neigh}(u)} X_v$ consisting of Pauli-X operators only. Pauli-Z-stabilizer generators are defined similarly based on the dual graph T_{sc^*}: For every $u \in \mathsf{V}^*_{\text{sc}}$, there is a stabilizer generator $B_u := \prod_{v \in \text{neigh}(u)} Z_v$ consisting of Pauli-Z operators only.

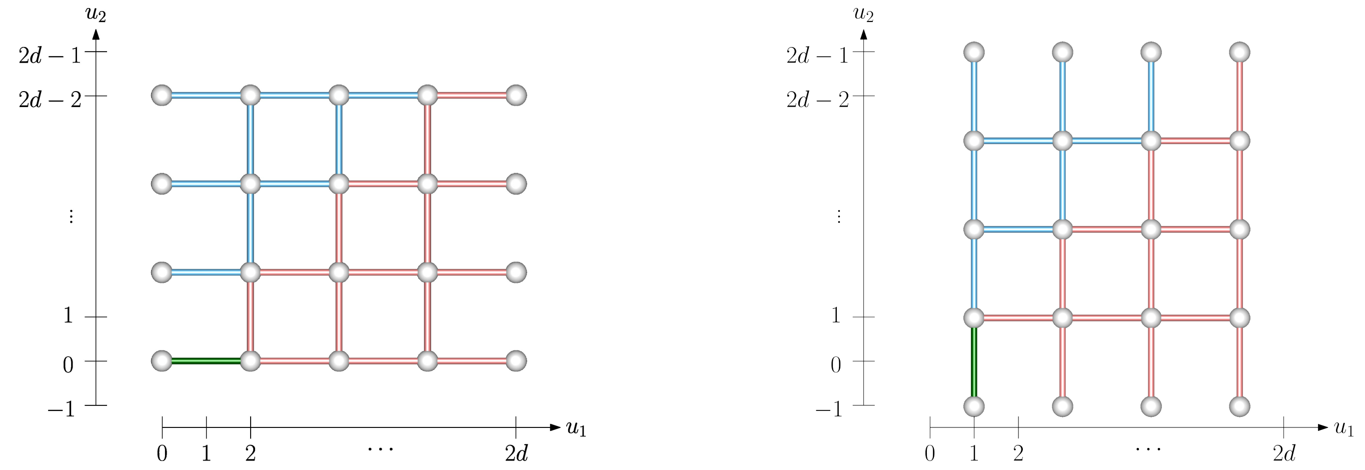

(a)

$\mathsf{T}_{\mathrm{sc}} = (\mathsf{V}_{\mathrm{sc}}, \mathsf{E}_{\mathrm{sc}})$		
subsets of E_{sc}	color	measurement
$\mathcal{X}$		X-basis
$\mathcal{Z}$		Z-basis
$\{q\}$		-

(b)

$\mathsf{T}_{\mathrm{sc}^*} = (\mathsf{V}_{\mathrm{sc}^*}, \mathsf{E}_{\mathrm{sc}^*})$		
subsets of $\mathsf{E}_{\mathrm{sc}^*}$	color	measurement
$\mathcal{X}$		X-basis
$\mathcal{Z}$		Z-basis
$\{q\}$		-

Figure 1: The surface code graph T_{sc}, and the dual graph $\mathsf{T}_{\mathrm{sc}^*}$. All qubits are situated at the midpoints of the edges. The protocol uses a measurement pattern where qubits belonging to $\mathcal{X}$ (midpoints of blue edges) are measured in the Pauli-X basis, qubits belonging to $\mathcal{Z}$ (midpoints of red edges) are measured in the Pauli-Z basis, and qubit q (midpoints of green edges) is not measured.

1.2 Graphs with internal vertices: boundary map and minimal matchings

Our decoder is based on an algorithm $\mathsf{MinMatch}$ which finds certain matchings of a graph with internal and external vertices. Let us first discuss some basic notions.

We consider pairs $(G = (V, E), V^{\mathrm{int}})$ where G is a graph and $V^{\mathrm{int}} \subset V$ a subset of vertices we call internal vertices. The complement $V^{\mathrm{ext}} := V \setminus V^{\mathrm{int}}$ will be referred to as the set of external vertices.

Define a boundary map $\partial : 2^E \to 2^{V^{\mathrm{int}}}$ that takes a subset $\mathcal{E} \subset E$ of edges to a subset $\partial\mathcal{E} \subset V^{\mathrm{int}}$ of internal vertices: An interval vertex $v \in V^{\mathrm{int}}$ belongs to $\partial\mathcal{E}$ if and only if the number of edges $e \in \mathcal{E}$ incident on v is odd. The map ∂ can be understood as the "usual" $\mathbb{Z}_2$-boundary map taking 1-chains (sets of edges) on the graph G to 0-chains (sets of vertices) on G, followed by a projection onto the subset of vertices V^{int}. We observe that, interpreted as a function $\partial : \mathbb{Z}_2^E \to \mathbb{Z}_2^{V^{\mathrm{int}}}$, the boundary map ∂ is $\mathbb{Z}_2$-linear.

Let $\mathcal{V} \subset V^{\mathrm{int}}$ be a subset of internal vertices. A subset $\mathcal{E} \subset E$ of edges is called a matching of $\mathcal{V}$ if $\partial\mathcal{E} = \mathcal{V}$. A matching $\mathcal{E}$ of $\mathcal{V}$ is called minimal if it its size $|\mathcal{E}|$ is minimal among all matchings of $\mathcal{V}$.

We note that for every graph $G = (V, E)$ with interval vertices V^{int}, there is a deterministic algorithm $\mathsf{MinMatch}$ which outputs a minimum matching $\mathsf{MinMatch}(\mathcal{V})$ of the given subset $\mathcal{V} \subset V^{\mathrm{int}}$ with runtime bounded by $O(|V|^3)$ [8, 6, 14]. This is obtained by suitably adapting Edmond's matching algorithm [3].

In Section 2, we will provide a general combinatorial analysis of matching on graphs with internal vertices. The "decoding" graphs of interest will be introduced in Section 1.5.

1.3 Measurement pattern used in protocol

Here we define the triangular measurement pattern used in our single-shot decoding protocol. Let

$$q := (1, 0) \in \mathcal{C}$$

be the qubit at the bottom left corner. The measurement pattern is then defined as follows (see Fig. 1): Every qubit belonging to

$$\mathcal{X} := \{(u_1, u_2) \in \mathcal{C} \setminus \{q\} \mid u_2 \geq u_1 + 1\}$$

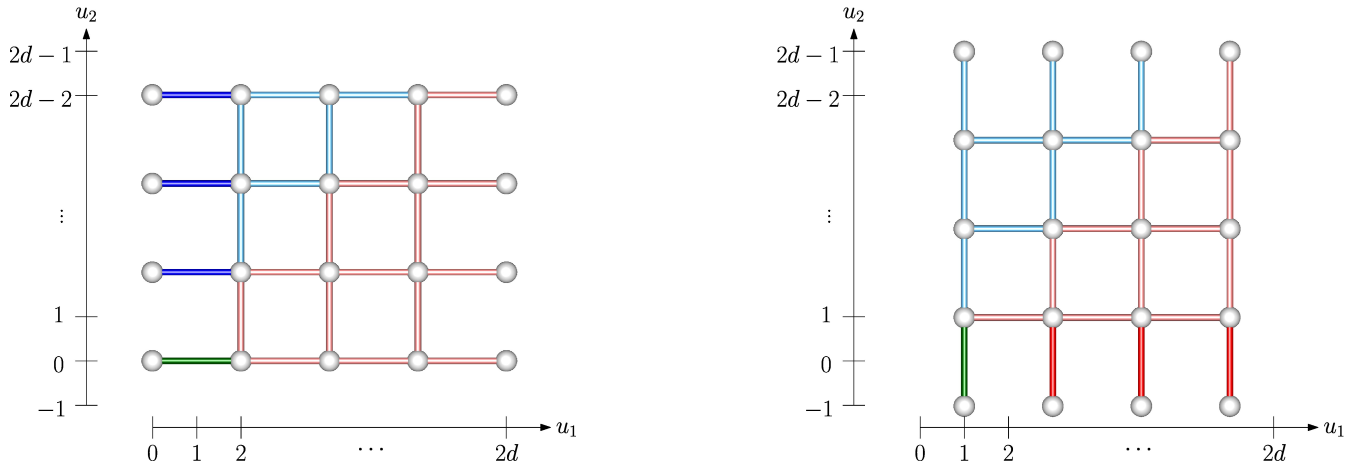

(a)

$\mathsf{T}_{\mathrm{sc}} = (\mathsf{V}_{\mathrm{sc}}, \mathsf{E}_{\mathrm{sc}})$		
subsets of E_{sc}	color	measurement
$\mathcal{L}_X$		X-basis
$\mathcal{X} \setminus \mathcal{L}_X$		X-basis
$\mathcal{Z}$		Z-basis
$\{q\}$		-

(b)

$\mathsf{T}_{\mathrm{sc}^*} = (\mathsf{V}_{\mathrm{sc}^*}, \mathsf{E}_{\mathrm{sc}^*})$		
subsets of $\mathsf{E}_{\mathrm{dec}^*}$	color	measurement
$\mathcal{X}$		X-basis
$\mathcal{L}_Z^*$		Z-basis
$\mathcal{Z} \setminus \mathcal{L}_Z^*$		Z-basis
$\{q\}$		-

Figure 2: The surface code graph T_{sc} and the dual graph $\mathsf{T}_{\mathrm{sc}^*}$, as well as the associated recovery sets $\mathcal{L}_X \subset \mathsf{E}_{\mathrm{sc}}$ (blue) and $\mathcal{L}_Z^* \subset \mathsf{E}_{\mathrm{sc}^*}$ (red).

is measured in the Pauli-X-basis. Every qubit belonging to

$$\mathcal{Z} := \{(u_1, u_2) \in \mathcal{C} \setminus \{q\} \mid u_2 < u_1 + 1\}$$

is measured in the Pauli-Z basis. The qubit at q is not measured.

1.4 Recovery sets

We will consider a special subset $\mathcal{L}_X \subset \mathsf{E}_{\mathrm{sc}}$ of edges, see Fig. 2. The set $\mathcal{L}_X$ is located on a vertical strip and consists of edges associated with the qubits

$$\mathcal{L}_X = \{(1, u_2) \mid u_2 \geq 2, u_2 \text{ even }\} \ . \tag{1}$$

(Here we use the convention that an edge $\{u, v\}$ is represented by its midpoint; it is, however, more natural to think of elements of $\mathcal{L}_X$ as edges of E_{sc}.)

Similarly, we consider a "dual" set $\mathcal{L}_Z^* \subset \mathsf{E}_{\mathrm{dec}^*}$ located on a horizontal strip defined by their midpoints

$$\mathcal{L}_Z^* = \{(u_1, 0) \mid u_2 \geq 3, u_2 \text{ odd }\} \ . \tag{2}$$

We will argue below that our protocol will need to estimate parities of certain strings restricted to $\mathcal{L}_X$ and $\mathcal{L}_Z^*$, respectively, in order to fix errors. This is because the associated qubits are closely related to the logical Pauli operators of the surface code, as discussed below. For this reason, we call $\mathcal{L}_X$ and $\mathcal{L}_Z^*$ the recovery sets.

1.5 Decoding subgraphs and internal vertices

Let us define the following "decoding" subgraph $\mathsf{T}_{\mathrm{dec}} = (\mathsf{V}_{\mathrm{dec}}, \mathsf{E}_{\mathrm{dec}})$ of T_{sc}, see Fig. 3a. The graph $\mathsf{T}_{\mathrm{dec}}$ is the subgraph induced by the set of vertices

$$\mathsf{V}_{\mathrm{dec}} = \{(u_1, u_2) \in \mathsf{V}_{\mathrm{sc}} \mid u_2 \geq u_1 > 0\} \ .$$

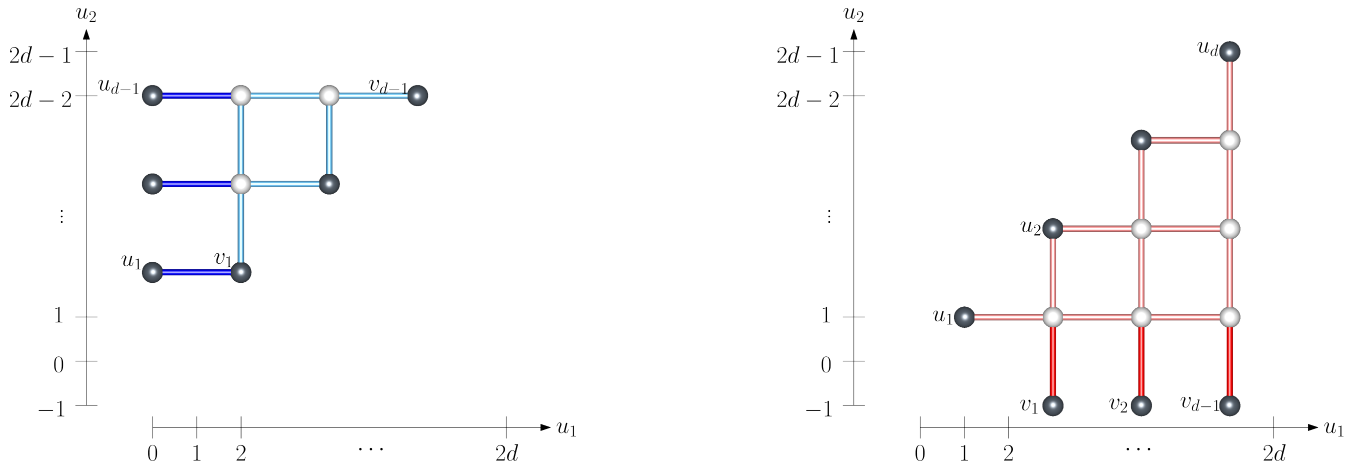

(a)

$\mathsf{T}_{\mathrm{dec}} = (\mathsf{V}_{\mathrm{dec}}, \mathsf{E}_{\mathrm{dec}})$		
subsets of $\mathsf{E}_{\mathrm{dec}}$	color	measurement
$\mathcal{L}_X$		X-basis
$\mathcal{X} \setminus \mathcal{L}_X$		X-basis

(b)

$\mathsf{T}_{\mathrm{dec}^*} = (\mathsf{V}_{\mathrm{dec}^*}, \mathsf{E}_{\mathrm{dec}^*})$		
subsets of $\mathsf{T}_{\mathrm{dec}}$	color	measurement
$\mathcal{L}_Z^*$		Z-basis
$\mathcal{Z} \setminus \mathcal{L}_Z^*$		Z-basis

Figure 3: The decoding graph $\mathsf{T}_{\mathrm{dec}}$ and the dual decoding graph $\mathsf{T}_{\mathrm{dec}^*}$. Their internal (external) vertices are illustrated as white (black, resp.) spheres. Edges are colored according to the subsets where their midpoints are contained (see the tables in the subfigures).

Alternatively, we may say that $\mathsf{T}_{\mathrm{dec}}$ is the subgraph of T_{sc} induced by the set of edges $\mathsf{E}_{\mathrm{dec}} := \mathcal{X}$. Explicitly, the edges of $\mathsf{T}_{\mathrm{dec}}$ are

$$\mathsf{E}_{\mathrm{dec}} = \{\{u,v\} \in \mathsf{V}_{\mathrm{dec}} \times \mathsf{V}_{\mathrm{dec}} \mid d(u,v) = 2\} \ .$$

Observe that the recovery set $\mathcal{L}_X \subset \mathsf{E}_{\mathrm{dec}}$ is a subset of the edges of $\mathsf{T}_{\mathrm{dec}}$. We will use $\mathsf{T}_{\mathrm{dec}}$ to estimate a parity of a certain string restricted to $\mathcal{L}_X$.

The graph $\mathsf{T}_{\mathrm{dec}}$ has distinguished internal vertices

$$\mathsf{V}_{\mathrm{dec}}^{\mathrm{int}} = \{u \in \mathsf{V}_{\mathrm{dec}} \mid \mathrm{neigh}(u) \subset \mathsf{E}_{\mathrm{dec}}\} \ . \tag{3}$$

A vertex $u \in \mathsf{V}_{\mathrm{dec}} \setminus \mathsf{V}_{\mathrm{dec}}^{\mathrm{int}}$ which is not internal is called external. We denote the set of external vertices of $\mathsf{T}_{\mathrm{dec}}$ by $\mathsf{V}_{\mathrm{dec}}^{\mathrm{ext}}$, see Fig. 3a. This definition is motivated by the fact that for every internal vertex $u \in \mathsf{V}_{\mathrm{dec}}^{\mathrm{int}}$, the "star" operator A_u of the surface code has support $\mathrm{supp}(A_u) \subset \mathsf{E}_{\mathrm{dec}}$ completely contained in the set of edges of $\mathsf{T}_{\mathrm{dec}}$. This is not the case for the external vertices.

The "dual decoding subgraph" $\mathsf{T}_{\mathrm{dec}^*} = (\mathsf{V}_{\mathrm{dec}^*}, \mathsf{E}_{\mathrm{dec}^*})$ of $\mathsf{T}_{\mathrm{sc}^*}$ is defined similarly: The graph $\mathsf{T}_{\mathrm{dec}^*}$ is the subgraph induced by the set of vertices

$$\mathsf{V}_{\mathrm{dec}^*} = \{(u_1,u_2) \in \mathsf{V}_{\mathrm{sc}} \mid u_1 \geq u_2 > 0\} \setminus \{(1,-1)\} \ ,$$

and its edges are $\mathsf{E}_{\mathrm{dec}^*} := \mathcal{Z}$. Explicitly, the edges of $\mathsf{T}_{\mathrm{dec}^*}$ are

$$\mathsf{E}_{\mathrm{dec}^*} = \{\{u,v\} \in \mathsf{V}_{\mathrm{dec}^*} \times \mathsf{V}_{\mathrm{dec}^*} \mid d(u,v) = 2\} \ .$$

Observe that the recovery set $\mathcal{L}_Z^*$ is a subset of the edges of $\mathsf{T}_{\mathrm{dec}^*}$. We will use $\mathsf{T}_{\mathrm{dec}^*}$ to estimate the parity of a certain string restricted to $\mathcal{L}_Z^*$.

The internal vertices of $\mathsf{T}_{\mathrm{dec}^*}$ are

$$\mathsf{V}_{\mathrm{dec}^*}^{\mathrm{int}} = \{u \in \mathsf{V}_{\mathrm{dec}^*} \mid \mathrm{neigh}(u) \subset \mathsf{E}_{\mathrm{dec}^*}\} \ . \tag{4}$$

Note that a vertex u of $\mathsf{T}_{\mathrm{dec}^*}$ is internal if and only if the "plaquette" operator B_u of the surface code has support $\mathrm{supp}(B_u) \subset \mathsf{E}_{\mathrm{dec}^*}$ contained in the set of edges of $\mathsf{T}_{\mathrm{dec}^*}$. A vertex $u \in \mathsf{V}_{\mathrm{dec}^*} \setminus \mathsf{V}_{\mathrm{dec}^*}^{\mathrm{int}}$ which is not internal is called external. We denote the set of external vertices of $\mathsf{T}_{\mathrm{dec}^*}$ by $\mathsf{V}_{\mathrm{dec}^*}^{\mathrm{ext}}$.

1.6 Description of the single-shot decoding procedure

We provide the description of algorithm for single-shot surface code decoding.

Algorithm 1 Single-shot surface code decoding

Require: A state on $(\mathbb{C}^2)^{\otimes \mathcal{C}}$, where $\mathcal{C}$ are the locations of qubits of a distance-d surface code.

1: Perform the following measurements: measure

every qubit in the set	in the	denote the outcome by
$\mathcal{X} \subset \mathsf{E}_{\mathrm{dec}}$	X-basis	$x \in \{0,1\}^{\mathcal{X}}$
$\mathcal{Z} \subset \mathsf{E}_{\mathrm{dec}^*}$	Z-basis	$z \in \{0,1\}^{\mathcal{Z}}$.

2: Compute the boundaries of the subsets of edges x and z in $\mathsf{T}_{\mathrm{dec}}$ and $\mathsf{T}_{\mathrm{dec}^*}$, respectively, i.e., set

$$\begin{aligned} s &\leftarrow \partial x \\ s^* &\leftarrow \partial^* z \ . \end{aligned}$$

3: Compute minimal matchings

$$\begin{aligned} m &\leftarrow \mathsf{MinMatch}(s) \\ m^* &\leftarrow \mathsf{MinMatch}^*(s^*) \end{aligned}$$

4: Compute the bits

$$\begin{aligned} \hat{s}^X &\leftarrow \langle\!\langle m, \mathcal{L}_X \rangle\!\rangle \\ \hat{s}^Z &\leftarrow \langle\!\langle m^*, \mathcal{L}_Z^* \rangle\!\rangle \ . \end{aligned}$$

5: Determine the bits

$$\begin{aligned} \hat{c}_X &\leftarrow \langle\!\langle x, \mathcal{L}_X \rangle\!\rangle \oplus \hat{s}^X \\ \hat{c}_Z &\leftarrow \langle\!\langle z, \mathcal{L}_Z^* \rangle\!\rangle \oplus \hat{s}^Z \end{aligned}$$

6: Apply $Z^{\hat{c}_X} X^{\hat{c}_Z}$ to q.

7: Return the resulting qubit state on qubit q.

The following notation is used in the algorithm: For any two sets $A, B \in \mathcal{C}$, we define

$$\langle\!\langle A, B \rangle\!\rangle := \bigoplus_{j \in \mathcal{C}} A_j B_j$$

where A and B are considered as strings (given by the characteristic function of the corresponding set) in $\{0,1\}^{\mathcal{C}}$ and $\oplus$ denotes XOR on bits. The value $\langle\!\langle A, B \rangle\!\rangle$ can equivalently be defined as the parity of $|A \cap B|$. Considering the set of strings $\{0,1\}^{\mathcal{C}}$ as a $\mathbb{Z}_2$-linear space, the map $\langle\!\langle \cdot, \cdot \rangle\!\rangle$ is clearly bilinear.

We also note that the runtime of Algorithm 1 is dominated by the computation of the matchings in step (3), i.e., runs in time $O(|\mathcal{C}|^3)$, see the remark at the end of Section 1.2.

2 A bound on matching with internal vertices

In this section, we provide a general combinatorial framework for analyzing decoding problems involving minimal matchings. These arguments will be applied in Section 3 to analyze the success probability of the single-shot decoding Algorithm 1 introduced in Section 1 in the presence of local stochastic noise.

We will also use these general concepts in Section 4 to analyze the robustness of our entanglement generation protocol based on the 3D cluster state.

We first show in Section 2.1 that a cycle, a set of edges in a graph with trivial boundary, can be decomposed as a disjoint union of simple closed paths and simple paths connecting two external vertices through internal vertices.

Next, in Section 2.2, we introduce the notion of a local stochastic subset of edges. This is a random variable $\mathcal{E}$ which is a random subset of edges, and whose distribution is exponentially decaying in the cardinality of the subset. We also introduce what we call the resilience function associated with a fixed subset $\mathcal{L}$ of edges. It is defined by a combinatorial expression in terms of a sum over simple closed paths and simple paths connecting two external endpoint vertices through internal vertices. We show that the value of resilience function is an upper bound on the probability that the parities of a local stochastic subset $\mathcal{E}$ and the minimum matching $\mathsf{MinMatch}(\partial\mathcal{E})$ of the boundary of $\mathcal{E}$ restricted to $\mathcal{L}$ differ. In particular, this will allow us to use the parity of $\mathsf{MinMatch}(\partial\mathcal{E}) \cap \mathcal{L}$ as an estimate of the parity of $\mathcal{E} \cap \mathcal{L}$. This is the key statement that we will subsequently use to analyze resilience against local stochastic noise.

2.1 Cycle decomposition

Let $(G = (V, E), V^{\mathrm{int}})$ be a graph with a distinguished set of internal vertices $V^{\mathrm{int}} \subset V$. Let $\partial : 2^E \to 2^{V^{\mathrm{int}}}$ be the boundary map defined in Section 1.2. A subset $\mathcal{E} \subset E$ is called a cycle if the boundary $\partial\mathcal{E} = \emptyset$ is trivial. We denote by $Z(G)$ the set of cyles on G. By the linearity of ∂, the set of cycles $Z(G)$ forms a linear subspace of $\mathbb{Z}_2^E$ called the cycle space of G. We will consider certain cycles which span $Z(G)$.

A closed loop $L = \{e_1, \ldots, e_\ell\} \subseteq E$ is given by a sequence $(e_1, \ldots, e_\ell)$ of distinct edges such that there exists a set of vertices $\{v_1, \ldots, v_\ell\}$ with the property that $e_j = \{v_j, v_{j+1}\}$ for all $j = 1, \ldots, \ell - 1$ and $e_\ell = \{v_\ell, v_1\}$. In particular, any closed loop L belongs to $Z(G)$, that is, $\partial L = \emptyset$. A simple closed loop $L = \{e_1, \ldots, e_\ell\}$ is a closed loop with the property that the vertices $\{v_1, \ldots, v_\ell\}$ are distinct. We note that any closed loop can be decomposed into a set of pairwise (edge-)disjoint simple closed loops. We will denote the set of simple closed loops on G by $Z_\circ(G)$.

We say that a simple (i.e., not self-intersecting) path $P = \{e_1, \ldots, e_\ell\} \subseteq E$ connects two distinct external vertices through internal vertices if there are distinct vertices $v_1, v_{\ell+1} \in V^{\mathrm{ext}}$ and $v_2, \ldots, v_\ell \in V^{\mathrm{int}}$ such that $e_j = \{v_j, v_{j+1}\}$ for $j = 1, \ldots, \ell$. Again, every such path P belongs to $Z(G)$ and the edges $\{e_j\}_{j=1}^{\ell}$ are pairwise distinct by construction. We will denote by $Z_{\mathrm{ext}}(G)$ the set of simple paths on G connecting distinct external vertices through internal vertices.

The union of $Z_\circ(G)$ and $Z_{\mathrm{ext}}(G)$ spans the cycle space $Z(G)$. In fact, a stronger property holds:

Lemma 2.1. *Let $\mathcal{Z} \in Z(G)$ be a cycle. Then $\mathcal{Z}$ is the disjoint union of simple closed loops and simple paths with endpoints in V^{ext} through internal vertices. That is, there is a family*

$$\{\mathcal{Z}_\alpha\}_\alpha \subset Z_\circ(G) \cup Z_{\mathrm{ext}}(G)$$

of cycles such that each $\mathcal{Z}_\alpha$ is either a simple closed loop or a simple path with distinct endpoints in V^{ext} passing through internal vertices, and

$$\mathcal{Z} = \bigcup_\alpha \mathcal{Z}_\alpha \qquad \textit{with} \qquad \mathcal{Z}_\alpha \cap \mathcal{Z}_\beta = \emptyset \textit{ for } \alpha \neq \beta \ .$$

Proof. Let $\mathcal{Z} \in Z(G)$ be a cycle. Suppose that there is a simple closed loop $L \in Z_\circ(G)$ that is contained in $\mathcal{Z}$. Then it follows immediately from the definitions that $\mathcal{Z}' := \mathcal{Z} \setminus L$ is also a cycle. Proceeding inductively, we may decompose $\mathcal{Z}$ as

$$\mathcal{Z} = \tilde{\mathcal{Z}} \cup \bigcup_{j=1}^{r} L_j \tag{5}$$

with pairwise disjoint simple closed loops $L_1, \ldots, L_r$ (with $r = 0$ if $\mathcal{Z}$ does not contain any simple closed loop) such that $\tilde{\mathcal{Z}}$ contain does not contain any (simple) closed loop.

We claim that $\tilde{\mathcal{Z}}$ is either empty or a union

$$\tilde{\mathcal{Z}} = \bigcup_{k=1}^{s} P_k \tag{6}$$

of pairwise disjoint paths $\{P_k\}_{k=1}^s \subset Z_{\mathrm{ext}}(G)$ with distinct endpoints in V^{ext}. Consider the following two cases:

(i) If $\tilde{\mathcal{Z}}$ does not contain an edge $e \in \tilde{\mathcal{Z}}$ with $e \cap V^{\mathrm{ext}} \neq \emptyset$, i.e., if $\tilde{\mathcal{Z}}$ only touches internal vertices of V, then the condition $\partial\tilde{\mathcal{Z}} = \emptyset$ implies that the number of edges belonging to $\tilde{\mathcal{Z}}$ which are incident on any internal vertex $v \in V^{\mathrm{int}}$ is even. Because $\tilde{\mathcal{Z}}$ does not contain any closed loop by construction, it follows that $\tilde{\mathcal{Z}} = \emptyset$.

(ii) Suppose there is an edge $e \in \tilde{\mathcal{Z}}$ such that $e \cap (V \setminus V^{\mathrm{int}}) \neq \emptyset$. Let us assume that $e = \{v, w\}$ where $v \in V \setminus V^{\mathrm{int}}$ is an external vertex. We can then construct a path $P = \{e_1, \ldots, e_\ell\} \subset \tilde{\mathcal{Z}}$ that has $v = v_s$ as starting point, first traverses the edge $e_1 := e$, and finally ends at an external vertex $v_e \neq v_s \in V \setminus V^{\mathrm{int}}$. Indeed, if $w \in V \setminus V^{\mathrm{int}}$ is an external vertex, then $P = \{e\}$ is such a path (of length 1). If $w \in V^{\mathrm{int}}$ is an internal vertex, then the condition $\tilde{\mathcal{Z}} \in Z(G)$ implies that the number of edges belonging to $\tilde{\mathcal{Z}}$ and incident on w is even. In particular, there is an edge e_2 that is distinct from e_1. We can proceed inductively to construct a path $\{e_1, \ldots, e_\ell\} \subset \tilde{\mathcal{Z}}$. Moreover, the path satisfies the desired properties, i.e., $P \in Z_{\mathrm{ext}}(G)$: The condition $\partial\tilde{\mathcal{Z}} = \emptyset$ guarantees that the path does not end at an internal vertex, and this implies that the constructed path only touches internal vertices except for the endpoints v_s and v_e which are external vertices. In addition, since $\tilde{\mathcal{Z}}$ does not contain any closed loop, the path is simple. In particular, its endpoint v_e must be an external vertex which is distinct from v_s.

Once we have found such a path P, we can remove P from $\tilde{\mathcal{Z}}$, i.e., we can consider the set $\tilde{\mathcal{Z}}' := \tilde{\mathcal{Z}} \setminus L$. It is easy to check that $\tilde{\mathcal{Z}}'$ is also a cycle which does not contain any closed loops. Proceeding inductively, we obtain the claim (6).

Combining (5) and (6) implies the claim. □

2.2 Local stochastic subsets and the resilience function

A random subset $\mathcal{E}$ of E is a random variable specified by a distribution over subsets of E. We call a random subset $\mathcal{E}$ local stochastic with parameter $p \in [0, 1]$ if and only if

$$\Pr[\mathcal{F} \subseteq \mathcal{E}] \leq p^{|\mathcal{F}|} \qquad \text{for all} \qquad \mathcal{F} \subset E\ .$$

This will be denoted $\mathcal{E} \sim \mathcal{N}(p)$.

We will consider the difference of a random subset $\mathcal{E}$ and a minimum matching $\mathsf{MinMatch}(\partial\mathcal{E})$ computed from the boundary $\partial\mathcal{E}$ of $\mathcal{E}$. More precisely, we consider the parity of this difference restricted to a subset $\mathcal{L} \subset E$. The following definition captures the relevant combinatorics of our estimates.

Definition 2.2. *Let $(G = (V, E), V^{\mathrm{int}})$ be a graph with internal vertices V^{int}. Let $\mathcal{L} \subset E$ be a subset of edges. Then the resilience function $\mathsf{res}_{\mathcal{L}} : [0, 1] \to \mathbb{R}$ of $\mathcal{L}$ is defined as*

$$\mathsf{res}_{\mathcal{L}}(p) := \sum_{\ell=1}^{\infty} \binom{\ell}{\lceil \ell/2 \rceil} \cdot |\{\mathcal{R} \in Z_\circ(G) \cup Z_{\mathrm{ext}}(G) \mid |\mathcal{R}| = \ell \text{ and } \langle\!\langle \mathcal{R}, \mathcal{L} \rangle\!\rangle = 1\}| \cdot p^{\lceil \ell/2 \rceil}\ . \tag{7}$$

The resilience function $\mathsf{res}_{\mathcal{L}} : [0, 1] \to \mathbb{R}$ provides the following upper bound on the probability that the parity of $\mathcal{E}$ and $\mathsf{MinMatch}(\partial\mathcal{E})$ restricted to $\mathcal{L}$ differ:

Proposition 2.3. *Let $\mathcal{E} \sim \mathcal{N}(p)$ be a local stochastic subset of E with parameter $p \in [0,1]$. Let $\mathcal{L} \subset E$ be a subset of edges. Then*

$$\Pr\left[\langle\!\langle \mathcal{E} \oplus \mathsf{MinMatch}(\partial\mathcal{E}), \mathcal{L}\rangle\!\rangle = 1\right] \leq \mathsf{res}_{\mathcal{L}}(p) \ .$$

Proof. The proof follows the proof of [1, Lemma 21] which is based on ideas of [5, 2]. Define

$$\mathrm{FAIL} := \{\mathcal{Y} \subseteq E \mid \langle\!\langle \mathcal{Y} \oplus \mathsf{MinMatch}(\partial\mathcal{Y}), \mathcal{L}\rangle\!\rangle = 1\}$$

such that the quantity of interest becomes $\Pr[\mathcal{E} \in \mathrm{FAIL}]$.

For any set $\mathcal{Y} \subseteq E$, the set $\mathcal{Y} \oplus \mathsf{MinMatch}(\partial\mathcal{Y})$ has trivial boundary by definition of $\mathsf{MinMatch}$. By Lemma 2.1, it belongs to the cycle space $Z(G)$ and can be decomposed as a union

$$\mathcal{Y} \oplus \mathsf{MinMatch}(\partial\mathcal{Y}) = \bigcup_{\alpha} \mathcal{Y}_\alpha \ ,$$

where each $\mathcal{Y}_\alpha \in Z_\circ(G) \cup Z_{\mathrm{ext}}(G)$ is either a simple closed loop or a simple path connecting external vertices through internal vertices, and where the sets $\mathcal{Y}_\alpha$ are pairwise disjoint. It follows that for any $\mathcal{Y} \subseteq E$ with $\mathcal{Y} \in \mathrm{FAIL}$, there is at least one $\mathcal{Y}_\alpha$ with the property that $\langle\!\langle \mathcal{Y}_\alpha, \mathcal{L}\rangle\!\rangle = 1$. That is, any such $\mathcal{Y}$ contains a subset $\mathcal{R} \in Z_\circ(G) \cup Z_{\mathrm{ext}}(G)$ which satisfies $\langle\!\langle \mathcal{R}, \mathcal{L}\rangle\!\rangle = 1$.

It follows that

$$\Pr[\mathcal{E} \in \mathrm{FAIL}] \leq \Pr\left[\exists \mathcal{R} \in Z_\circ(G) \cup Z_{\mathrm{ext}}(G) : \mathcal{R} \subset \mathcal{E} \oplus \mathsf{MinMatch}(\partial\mathcal{E}) \text{ and } \langle\!\langle \mathcal{R}, \mathcal{L}\rangle\!\rangle = 1\right] \ . \tag{8}$$

For simplicity, let us define

$$\mathcal{P}(\mathcal{L}) := \{\mathcal{R} \in Z_\circ(G) \cup Z_{\mathrm{ext}}(G) \mid \langle\!\langle \mathcal{R}, \mathcal{L}\rangle\!\rangle = 1\} \ .$$

With the union bound applied to (8) we obtain

$$\Pr[\mathcal{E} \in \mathrm{FAIL}] \leq \sum_{\mathcal{R} \in \mathcal{P}(\mathcal{L})} \Pr\left[\mathcal{R} \subseteq \mathcal{E} \oplus \mathsf{MinMatch}(\partial\mathcal{E})\right] \ . \tag{9}$$

Let $\mathcal{E} \subset E$ be arbitrary and set $\mathcal{M} := \mathsf{MinMatch}(\partial\mathcal{E})$. Suppose that $\mathcal{R} \in \mathcal{P}(\mathcal{L})$ satisfies

$$\mathcal{R} \subseteq \mathcal{E} \oplus \mathcal{M} \ .$$

We claim that at least half of the edges of $\mathcal{R}$ do not belong to $\mathcal{M}$, i.e.,

$$|\mathcal{R} \setminus \mathcal{M}| \geq |\mathcal{R}|/2 \ . \tag{10}$$

For the sake of contradiction, assume that

$$|\mathcal{R} \setminus \mathcal{M}| < |\mathcal{R}|/2 \ . \tag{11}$$

Then the set $\mathcal{M}' := \mathcal{R} \oplus \mathcal{M}$ is a matching of $\partial\mathcal{E}$ since

$$\begin{aligned} \partial\mathcal{M}' &= \partial(\mathcal{R} \oplus \mathcal{M}) \\ &= \partial\mathcal{R} \oplus \partial\mathsf{MinMatch}(\partial\mathcal{E}) \\ &= \partial\mathcal{E} \ , \end{aligned}$$

where we used that every $\mathcal{R} \in \mathcal{P}(\mathcal{L})$ has trivial boundary $\partial\mathcal{R} = \emptyset$. Furthermore, we have

$$\begin{aligned} |\mathcal{M}'| &= |\mathcal{R} \setminus \mathcal{M}| + |\mathcal{M} \setminus \mathcal{R}| \\ &= |\mathcal{R} \setminus \mathcal{M}| + |\mathcal{M}| - |\mathcal{M} \cap \mathcal{R}| \\ &= |\mathcal{R} \setminus \mathcal{M}| + |\mathcal{M}| - (|\mathcal{R}| - |\mathcal{R} \setminus \mathcal{M}|) \\ &= (2|\mathcal{R} \setminus \mathcal{M}| - |\mathcal{R}|) + |\mathcal{M}| \\ &< |\mathcal{M}| \ , \end{aligned}$$

where we used our assumption (11). Thus $\mathcal{M}'$ is a matching of $\partial\mathcal{E}$ of smaller cardinality than $\mathcal{M}$, contradicting the fact that $\mathcal{M}$ is a minimum matching of $\partial\mathcal{E}$.

Eq. (10) shows that $\mathcal{R} \subset \mathcal{E} \oplus \mathcal{M}$ together with $\mathcal{R} \in \mathcal{P}(\mathcal{L})$ imply that

$$|\mathcal{R} \cap \mathcal{E}| \geq |\mathcal{R}|/2 \ . \tag{12}$$

Indeed, the fact that $\mathcal{R}$ is contained in the symmetric difference $\mathcal{E} \oplus \mathcal{M}$ implies that $\mathcal{R}$ is the disjoint union $\mathcal{R} = (\mathcal{R}\cap\mathcal{E}) \cup (\mathcal{R}\cap\mathcal{M})$ and thus $\mathcal{R}\cap\mathcal{E} \supseteq \mathcal{R}\setminus\mathcal{M}$. This implies the claim (12) together with (10).

With (12), we conclude that for every $\mathcal{R} \in \mathcal{P}(\mathcal{L})$, we have $|\mathcal{R}\cap\mathcal{E}| \geq \lceil|\mathcal{R}|/2\rceil$ (because $|\mathcal{R}\cap\mathcal{E}|$ is an integer) and thus

$$\begin{aligned}\Pr\left[\mathcal{R} \subseteq \mathcal{E} \oplus \mathsf{MinMatch}\left(\partial\mathcal{E}\right)\right] &\leq \Pr\left[|\mathcal{R}\cap\mathcal{E}| \geq |\mathcal{R}|/2\right] \\ &\leq \Pr\left[\exists \delta \subseteq \mathcal{R} \ \text{ with } |\delta| = \lceil|\mathcal{R}|/2\rceil \text{ and } \delta \subseteq \mathcal{E}\right] \ .\end{aligned}$$

Here we used that for any set $\mathcal{E}$ with $|\mathcal{R}\cap\mathcal{E}| \geq \lceil|\mathcal{R}|/2\rceil$, there is a subset $\delta \subseteq \mathcal{R}\cap\mathcal{E}$ of size exactly equal to $\lceil|\mathcal{R}|/2\rceil$. We can sum over subsets $\delta \subset \mathcal{R}$ of cardinality $|\delta| = \lceil|\mathcal{R}|/2\rceil$ to obtain with the union bound

$$\begin{aligned}\Pr\left[\mathcal{R} \subseteq \mathcal{E} \oplus \mathsf{MinMatch}\left(\partial\mathcal{E}\right)\right] &\leq \sum_{\substack{\delta\subseteq\mathcal{R} \\ |\delta|=\lceil|\mathcal{R}|/2\rceil}} \Pr\left[\delta \subseteq \mathcal{E}\right] \\ &\leq \binom{|\mathcal{R}|}{\lceil|\mathcal{R}|/2\rceil} \cdot p^{\lceil|\mathcal{R}|/2\rceil} \ ,\end{aligned}$$

where we used that $\mathcal{E} \sim \mathcal{N}(p)$, i.e., the fact that $\mathcal{E}$ is local stochastic. The claim follows by inserting this into (9) and applying the definition of $\mathsf{res}_{\mathcal{L}}(p)$. $\square$

3 Single-shot decoding under local stochastic noise

In this section, we establish our main result about single-shot decoding of the 2D surface code. We consider a situation where a surface-code encoded state is corrupted by a local stochastic error, and where the output state ρ_{out} is obtained by running Algorithm 1 on this corrupted logical state.

In Section 3.1, we consider a half-encoded Bell state $|\Phi_{\mathcal{C}R}\rangle$ defined on the surface code system $\mathcal{C}$ of the surface code and an auxiliary qubit system R. Supposing that Algorithm 1 is executed on the surface code system of the Bell state corrupted by a Pauli error E on the surface code qubits, we show that the output state then is one of the Bell basis states $\{\Phi_{(\alpha,\beta)}\}_{\alpha,\beta\in\{0,1\}}$ defined as

$$\left|\Phi_{(\alpha,\beta)}\right\rangle = (I\otimes Z^{\alpha}X^{\beta})\,|\Phi\rangle\ ,\qquad \text{where}\qquad |\Phi\rangle = \frac{1}{\sqrt{2}}\left(|00\rangle+|11\rangle\right)\ , \tag{13}$$

and find corresponding expressions for α and β.

In Section 3.2, we compute upper bounds on the resilience function of the recovery sets $\mathcal{L}_X$ and $\mathcal{L}^*_Z$. Using these bounds and the expressions for (α,β) obtained in Section 3.1, we prove in Section 3.3 that for any input state corrupted by a local stochastic error of strength p below a certain threshold, the overlap $\langle\Psi|\rho_{\mathrm{out}}|\Psi\rangle$ between Ψ and the output state ρ_{out} is at least $1-O(p)$.

3.1 Decoding on a corrupted half-encoded Bell state

In the following, we use the logical Pauli operators

$$\begin{aligned}\overline{X} &= X(\mathcal{L}_X)X_q\\ \overline{Z} &= Z(\mathcal{L}^*_Z)Z_q\end{aligned}$$

of the surface code. We consider the action of Algorithm 1 on a certain bipartite state of the form $\Phi_{\mathcal{C}R}\in(\mathbb{C}^2)^{\otimes\mathcal{C}\cup\{R\}}\cong(\mathbb{C}^2)^{\otimes\mathcal{C}}\otimes\mathbb{C}^2$, where R is a reference qubit. Specifically, we are interested in the Bell state $\Phi_{\mathcal{C}R}$ between a surface-code encoded logical qubit and the reference qubit R. We call this state the half-encoded Bell state (since the qubit R is "physical", i.e., not encoded). The state has stabilizer generators $\{A_v\}_v\cup\{B_f\}_f\cup\{S^X,S^Z\}$, where $\{A_v\}_v$ and $\{B_f\}_f$ are the usual surface code generators (associated with vertices and faces of the surface code lattice), and where

$$\begin{array}{rcccl} S^X &:=& \overline{X}X_R &=& X(\mathcal{L}_X)X_qX_R\\ S^Z &:=& \overline{Z}Z_R &=& Z(\mathcal{L}^*_Z)Z_qX_R\ , \end{array} \tag{14}$$

are the Bell state stabilizer generators. In addition to the stabilizer generators $\{S^X,S^Z\}$, the following sets $\{S^u\}_{u\in\mathsf{V}^{\mathrm{int}}_{\mathrm{dec}}}$ and $\{S^{u^*}\}_{u^*\in\mathsf{V}^{\mathrm{int}}_{\mathrm{dec}^*}}$ of stabilizers of the state $\Phi_{\mathcal{C}R}$ play a special role in our argument. They are defined as follows: Any internal vertex $u\in\mathsf{V}^{\mathrm{int}}_{\mathrm{dec}}$ of $\mathsf{T}_{\mathrm{dec}}$ is associated with it a stabilizer generator S^u of the surface code consisting of Pauli-X-operators only, namely $S^u=A_u$. In a similar vein, any internal vertex $u^*\in\mathsf{V}^{\mathrm{int}}_{\mathrm{dec}^*}$ of $\mathsf{T}_{\mathrm{dec}^*}$ gives rise to a stabilizer S^{u^*} consisting of Z-type operators, namely the face operator B_{u^*} when u^* is considered as a face of the original surface code lattice T_{sc}. Observe that for each $u\in\mathsf{V}^{\mathrm{int}}_{\mathrm{dec}}$, the support $\mathrm{supp}(S^u)=\mathrm{Inci}(u)$ of S^u is equal to the set of edges incident on u in the graph $\mathsf{T}_{\mathrm{dec}}$. Similarly, for each $u\in\mathsf{V}^{\mathrm{int}}_{\mathrm{dec}^*}$, we have $\mathrm{supp}(S^u)=\mathrm{Inci}^*(u)$ where $\mathrm{Inci}^*(u)$ is the set of edges incident on u in the graph $\mathsf{T}_{\mathrm{dec}^*}$. That is, we have

$$S^u=\begin{cases}\prod_{v\in\mathrm{Inci}(u)}X_v & \text{for}\quad u\in\mathsf{V}^{\mathrm{int}}_{\mathrm{dec}}\\ \prod_{v\in\mathrm{Inci}(u)}Z_v & \text{for}\quad u\in\mathsf{V}^{\mathrm{int}}_{\mathrm{dec}^*}\ .\end{cases} \tag{15}$$

Consider the result of running the algorithm on a corrupted state

$$|\Psi_{\mathrm{in}}\rangle = E\,|\Phi_{\mathcal{C}R}\rangle \tag{16}$$

for a fixed Pauli error E. We assume here that $\mathrm{supp}(E) \subseteq \mathcal{C}$, i.e., the error acts on the surface code qubits only (and does not affect the reference system R). Let

$$|\psi_{\mathrm{pm}}(x,z,E)\rangle = \frac{1}{\sqrt{p(x,z|E)}} \left(\langle x|_{\mathcal{X}} H(\mathcal{X})^\dagger \otimes \langle z|_{\mathcal{Z}} \otimes I_{\{q,R\}} \right) E \,|\Phi_{\mathcal{C}R}\rangle \tag{17}$$

be the state obtained after Step 1 of the algorithm on qubit q. Here we denote by $p(x,z|E)$ the probability of obtaining the outcomes (x,z) given a Pauli error E, i.e., for the input state (16). It will be convenient in the following to use the definition

$$\mathsf{Syn}(P,Q) = \begin{cases} 0 & \text{if } P \text{ and } Q \text{ commute} \\ 1 & \text{otherwise} \end{cases}$$

for any two Pauli operators P and Q. Then the following holds:

Lemma 3.1. *Let S^X and S^Z be the operators* (14)*. Let*

$$\begin{aligned} c_X &:= \langle\!\langle x, \mathcal{L}_X \rangle\!\rangle \oplus \mathsf{Syn}(S^X, E) \\ c_Z &:= \langle\!\langle z, \mathcal{L}_Z^* \rangle\!\rangle \oplus \mathsf{Syn}(S^Z, E) \ . \end{aligned}$$

Then the post-measurement state $\psi_{\mathrm{pm}}(x,z,E)$ on the two qubits q,R after Step 1 of Algorithm 1, given an initial error E and measurement outcomes (x,z) is the Bell state (cf. (13)*)*

$$|\psi_{\mathrm{pm}}(x,z,E)\rangle = \left|\Phi_{(c_X,c_Z)}\right\rangle \ .$$

Proof. The claim is an immediate consequence of the fact that S^X and S^Z are stabilizers of $|\Phi_{\mathcal{C}R}\rangle$, as well as the expressions (14) of the stabilizer generators: Replacing $|\Phi_{\mathcal{C}R}\rangle$ in (17) by $S^X|\Phi_{\mathcal{C}R}\rangle$, we observe that the post-measurement state $|\psi_{\mathrm{pm}}\rangle = |\psi_{\mathrm{pm}}(x,z,E)\rangle$ satisfies

$$\begin{aligned} \sqrt{p}\,|\psi_{\mathrm{pm}}\rangle &= \left(\langle x|_{\mathcal{X}} H(\mathcal{X})^\dagger \otimes \langle z|_{\mathcal{Z}} \otimes I_q \right) E S^X |\Phi_{\mathcal{C}R}\rangle \\ &= (-1)^{\mathsf{Syn}(S^X,E)} \left(\langle x|_{\mathcal{X}} H(\mathcal{X})^\dagger \otimes \langle z|_{\mathcal{Z}} \otimes I_q \otimes I_R \right) S^X E \,|\Phi_{\mathcal{C}R}\rangle \\ &= (-1)^{\langle x,\mathcal{L}_X\rangle \oplus \mathsf{Syn}(S^X,E)} \left(\langle x|_{\mathcal{X}} H(\mathcal{X})^\dagger \otimes \langle z|_{\mathcal{Z}} \otimes X_q X_R \right) E\,|\Phi_{\mathcal{C}R}\rangle \ , \end{aligned}$$

where $p := p(x,z|E)$ and where we used (14) as well as the identity

$$X(\mathcal{L}_X) H(\mathcal{X})\,|x\rangle_{\mathcal{X}} = (-1)^{\langle x,\mathcal{L}_X\rangle} H(\mathcal{X})\,|x\rangle_{\mathcal{X}} \ .$$

We conclude that

$$\begin{aligned} |\psi_{\mathrm{pm}}\rangle &= (-1)^{\langle x,\mathcal{L}_X\rangle \oplus \mathsf{Syn}(S^X,E)} X_q X_R\,|\psi_{\mathrm{pm}}\rangle \\ &= (-1)^{c_X} X_q X_R\,|\psi_{\mathrm{pm}}\rangle \ , \end{aligned}$$

by definition of c_X.

In an analogous manner, we can check that $|\psi_{\mathrm{pm}}\rangle = (-1)^{c_Z} Z_q Z_R\,|\psi_{\mathrm{pm}}\rangle$ by replacing $|\Phi_{\mathcal{C}R}\rangle$ in (17) by $S^Z|\Phi_{\mathcal{C}R}\rangle$. Thus the two-qubit state $|\psi_{\mathrm{pm}}\rangle$ on systems q,R is stabilized by $\{(-1)^{c_X} X_q X_R, (-1)^{c_Z} Z_q Z_R\}$. This implies the claim. □

According to Lemma 3.1, the post-measurement state is fully determined by the single-qubit measurement results (x,z) and the bits $\mathsf{Syn}(S^X,E)$ and $\mathsf{Syn}(S^Z,E)$. To analyze these bits, we introduce decomposition

$$E = E^X E^Z \tag{18}$$

of E into a product of Pauli-X and Pauli-Z operators E^X and E^Z, respectively. (We note that if $E \sim \mathcal{N}(p)$ is a local stochastic error of strength p, the same is true for both E^X and E^Z, i.e., $E^X \sim \mathcal{N}(p)$ and $E^Z \sim \mathcal{N}(p)$, a fact we will use below.)

In (18), the global phase is ignored since we will only need the supports $\mathrm{supp}(E^X)$ and $\mathrm{supp}(E^Z)$. We show that the bits $\mathsf{Syn}(S^X,E)$ and $\mathsf{Syn}(S^Z,E)$ are determined by the physical errors on qubits $\{q_1,q_2\}$ as well as a certain restriction of the error as specified in the following statement:

Lemma 3.2. *Let E be a Pauli operator on $\mathcal{C}$ and let S^X and S^Z be the Pauli operators defined by* (14)*. Decompose E into a product $E = E^X E^Z$ of Pauli-X- and Pauli-Z-operators, respectively. Then*

$$\mathsf{Syn}(S^X, E) = \langle\!\langle \{q\}, \mathrm{supp}(E^Z) \rangle\!\rangle \oplus \langle\!\langle \mathcal{L}_X, \mathrm{supp}(E^X) \rangle\!\rangle \tag{19}$$
$$\mathsf{Syn}(S^Z, E) = \langle\!\langle \{q\}, \mathrm{supp}(E^Z) \rangle\!\rangle \oplus \langle\!\langle \mathcal{L}_Z^*, \mathrm{supp}(E^Z) \rangle\!\rangle \ . \tag{20}$$

Proof. Let E be an arbitrary Pauli operator on $\mathcal{C}$. With expression (14) for S^X, we have

$$\begin{aligned}\mathsf{Syn}(S^X, E) &= \mathsf{Syn}\left(X_q X_R X\left(\mathcal{L}_X\right), E\right) \\ &= \langle\!\langle \{q\}, \mathrm{supp}(E^X) \rangle\!\rangle \oplus \langle\!\langle \mathcal{L}_X, \mathrm{supp}(E^X) \rangle\!\rangle\end{aligned}$$

since E acts trivially on the reference system R. The claim for $\mathsf{Syn}(S^Z, E)$ follows similarly. □

Unfortunately, the operators E^X and E^Z are not determined by the measurement results (x, z). In particular, the bits $\mathsf{Syn}(S^X, E)$ and $\mathsf{Syn}(S^Z, E)$ cannot be computed from the measurement outcomes. Algorithm 1 therefore generates estimates $\hat{s}^X$ and $\hat{s}^Z$ for these bits, see step 4.

To motivate the definition of these bits, and to derive a sufficient condition for when the algorithm is correct (i.e., generates the Bell state Φ), we discuss how the measurement results (x, z) constrain the error E, or more precisely the operators E^X and E^Z. That is, we consider what information can be gathered from these measurement outcomes. Observe that the operators (E^X, E^Z) are fully determined by the supports $\mathrm{supp}(E^X)$ and $\mathrm{supp}(E^Z)$. We obtain constraints on these sets expressed in terms of the strings s, s^* computed in Step 2 of Algorithm 1.

Lemma 3.3. *The strings $s \in \{0,1\}^{\mathsf{V}_{\mathrm{dec}}^{\mathrm{int}}}$ and $s^* \in \{0,1\}^{\mathsf{V}_{\mathrm{dec}*}^{\mathrm{int}}}$ computed (from the measurement outcomes (x, z)) in Step 2 satisfy*

$$s = \partial \mathrm{supp}(E^Z) \tag{21}$$
$$s^* = \partial^* \mathrm{supp}(E^X) \ . \tag{22}$$

Proof. Let $s = (s_u) \in \{0,1\}^{\mathsf{V}_{\mathrm{dec}}^{\mathrm{int}}}$ be the string computed in step 2, i.e., $s = \partial x$. Let $u \in \mathsf{V}_{\mathrm{dec}}^{\mathrm{int}}$. Since $s_u = (\partial x)_u = \langle\!\langle x, \mathrm{Inci}(u) \rangle\!\rangle$ and $\left(\partial \mathrm{supp}(E^Z)\right)_u = \langle\!\langle \mathrm{Inci}(u), \mathrm{supp}(E^Z) \rangle\!\rangle$, we need to show that

$$\langle\!\langle x, \mathrm{Inci}(u) \rangle\!\rangle = \langle\!\langle \mathrm{Inci}(u), \mathrm{supp}(E^Z) \rangle\!\rangle \qquad \text{for every} \qquad u \in \mathsf{V}_{\mathrm{dec}}^{\mathrm{int}} \ . \tag{23}$$

Note that S^u stabilizes the (uncorrupted) state $|\Phi_{\mathcal{C}R}\rangle$ (by definition of the latter). Replacing $|\Phi_{\mathcal{C}R}\rangle$ in (17) by $S^u |\Phi_{\mathcal{C}R}\rangle$ yields

$$\begin{aligned}|\psi_{\mathrm{pm}}\rangle &= \frac{1}{\sqrt{p}} \left(\langle x|_{\mathcal{X}} H(\mathcal{X})^\dagger \otimes \langle z|_{\mathcal{Z}} \otimes I_{\{q,R\}} \right) E S^u W \left|0^{\mathcal{C}}\right\rangle \\ &= \frac{1}{\sqrt{p}} (-1)^{\mathsf{Syn}(S^u, E)} \left(\langle x|_{\mathcal{X}} H(\mathcal{X})^\dagger \otimes \langle z|_{\mathcal{Z}} \otimes I_{\{q,R\}} \right) S^u E W \left|0^{\mathcal{C}}\right\rangle \\ &= (-1)^{\mathsf{Syn}(S^u, E) \oplus \langle x, \mathrm{Inci}(u) \rangle} |\psi_{\mathrm{pm}}\rangle \ .\end{aligned} \tag{24}$$

Here the third equality follows from the identity

$$S^u H(\mathcal{X}) |x\rangle = (-1)^{\langle x, \mathrm{Inci}(u) \rangle} H(\mathcal{X}) |x\rangle$$

which is a consequence of the definition of S^u (as an X-type stabilizer generator the surface code), as well as the fact that for $u \in \mathsf{V}_{\mathrm{dec}}^{\mathrm{int}}$, the set $\mathrm{supp}(S^u)$ does not contain q for any $u \in \mathsf{V}_{\mathrm{dec}}^{\mathrm{int}}$, see Eq. (15). Identity (24) implies that

$$\mathsf{Syn}(S^u, E) = \langle\!\langle x, \mathrm{Inci}(u) \rangle\!\rangle \qquad \text{for} \qquad u \in \mathsf{V}_{\mathrm{dec}}^{\mathrm{int}} \ . \tag{25}$$

Observe that according to (15), we also have

$$\mathsf{Syn}(S^u, E) = \mathsf{Syn}\left(\left(\prod_{v\in \mathrm{Inci}(u)} X_v\right), E\right) = \langle\!\langle \mathrm{Inci}(u), \mathrm{supp}(E^Z)\rangle\!\rangle \qquad \text{for any} \qquad u \in \mathsf{V}_{\mathrm{dec}}^{\mathrm{int}} \ . \tag{26}$$

Combining (25) with (26) gives the claim (23).

The claim for s^* can be proved in an analogous manner using (15). □

We now argue that the sets $m \subset \mathsf{E}_{\mathrm{dec}}$ and $m^* \subset \mathsf{E}_{\mathrm{dec}^*}$ computed in Step 3 of Algorithm 1 can be considered to be proxys for the sets $\mathrm{supp}(E^Z) \subset \mathsf{E}_{\mathrm{dec}}$ and $\mathrm{supp}(E^X) \subset \mathsf{E}_{\mathrm{dec}^*}$. More precisely, we may define Pauli errors

$$\begin{array}{rcl} \widehat{E}^X & = & X(m^*) \\ \widehat{E}^Z & = & Z(m) \ . \end{array} \tag{27}$$

We note that by definition of m and m^* as boundaries of subsets of edges in the graph $\mathsf{T}_{\mathrm{dec}}$ and $\mathsf{T}_{\mathrm{dec}^*}$, respectively, and the definition of these graphs, neither m nor m^* contains the qubit q. Thus

$$\{q\} \cap \mathrm{supp}(\widehat{E}^X) = \{q\} \cap \mathrm{supp}(\widehat{E}^Z) = \emptyset \ . \tag{28}$$

We trivially have

$$\begin{aligned} m &= \mathrm{supp}(\widehat{E}^Z) \\ m^* &= \mathrm{supp}(\widehat{E}^X) \end{aligned}$$

by the definition (27) of these operators, and it follows immediately from the definition of m and m^* that $(\widehat{E}^X, \widehat{E}^Z)$ satisfy constraints analogous to the constraints (21), (22) obeyed by (E^X, E^Z), i.e., we have

$$\begin{aligned} s &= \partial \mathrm{supp}(\widehat{E}^Z) \\ s^* &= \partial^* \mathrm{supp}(\widehat{E}^X) \ . \end{aligned}$$

Thus $\widehat{E} := \widehat{E}^X \widehat{E}^Z$ is an error consistent with the observed syndrome (s, s^*) with the property that $\widehat{E}^Z$ and $\widehat{E}^X$ each are minimum weight matchings in $\mathsf{T}_{\mathrm{dec}}$ and $\mathsf{T}_{\mathrm{dec}^*}$, respectively.

Because the error $\widehat{E}$ can be computed from the measurement outcomes, this motivates using the pair $\left(\mathsf{Syn}(S^X, \widehat{E}), \mathsf{Syn}(S^Z, \widehat{E})\right)$ as an estimate for $\left(\mathsf{Syn}(S^X, E), \mathsf{Syn}(S^Z, E)\right)$. Indeed, this is the reasoning underlying the definition

$$\begin{array}{rcl} \hat{s}^X & := & \langle\!\langle \mathcal{L}_X, m\rangle\!\rangle \\ \hat{s}^Z & := & \langle\!\langle \mathcal{L}_Z^*, m^*\rangle\!\rangle \end{array} \tag{29}$$

in Step 4 of the algorithm: It is easy to check that $(\hat{s}^X, \hat{s}^Z)$ defined in this way satisfy

$$\begin{array}{rcl} \hat{s}^X & = & \mathsf{Syn}(S^X, \widehat{E}) \\ \hat{s}^Z & = & \mathsf{Syn}(S^Z, \widehat{E}) \end{array} \tag{30}$$

because of Expressions (19), (20): Indeed, we have for example

$$\begin{aligned} \mathsf{Syn}(S^X, \widehat{E}) &= \mathsf{Syn}\left(X(\mathcal{L}_X) X_q X_R, \widehat{E}^X \widehat{E}^Z\right) \\ &= \left\langle\!\!\left\langle \mathcal{L}_X, \mathrm{supp}(\hat{E}^Z)\right\rangle\!\!\right\rangle \\ &= \langle\!\langle \mathcal{L}_X, m\rangle\!\rangle \end{aligned}$$

where we used the definition (14) in the first step, and the fact that $\hat{E}^Z$ has no support on $\{q, R\}$ in the second step (cf. (28)). Identity (30) shows that the pair $(\hat{s}^X, \hat{s}^Z)$ is a natural estimate for the bits $\big(\mathsf{Syn}(S^X, E), \mathsf{Syn}(S^Z, E)\big)$, the idea being that $\widehat{E}$ is a decent estimate of E.

Having motivated the algorithm, let us show that Algorithm 1 produces a Bell state $\Phi_{(\alpha,\beta)}$. Below, we will use the expressions for (α, β) computed here below to analyze the success probability of the protocol (i.e., the probability that $(\alpha, \beta) = (0, 0)$) for local stochastic noise.

Theorem 3.4. *Let $E = E^X E^Z$ be an arbitrary Pauli operator on $(\mathbb{C}^2)^{\otimes \mathcal{C}}$. Suppose Algorithm 1 is executed with an auxiliary qubit system R and the initial state $|\Psi_{in}\rangle$ specified by (16), i.e., the (half-encoded) Bell state $\Phi_{\mathcal{C}R}$ corrupted by the error E. Then the output of Algorithm 1 on qubits q, R is the Bell state $\big|\Phi_{(\alpha,\beta)}\big\rangle$, where*

$$\begin{aligned}\alpha &= \big\langle\!\big\langle \{q\}, \mathrm{supp}(E^Z)\big\rangle\!\big\rangle \oplus \big\langle\!\big\langle \mathrm{supp}(E^Z) \oplus \mathsf{MinMatch}(\partial \mathrm{supp}(E^Z)), \mathcal{L}_X\big\rangle\!\big\rangle \qquad \textit{and} \\ \beta &= \big\langle\!\big\langle \{q\}, \mathrm{supp}(E^X)\big\rangle\!\big\rangle \oplus \big\langle\!\big\langle \mathrm{supp}(E^X) \oplus \mathsf{MinMatch}^*(\partial \mathrm{supp}(E^X)), \mathcal{L}_Z^*\big\rangle\!\big\rangle\ . \end{aligned} \tag{31}$$

Proof. We first show that the output is the Bell state $\Phi_{(\alpha,\beta)}$ with

$$\begin{aligned}\alpha &= \big\langle\!\big\langle \{q\}, \mathrm{supp}(E^Z)\big\rangle\!\big\rangle \oplus \big\langle\!\big\langle \mathrm{supp}(E^Z) \oplus m, \mathcal{L}_X\big\rangle\!\big\rangle \qquad \text{and} \\ \beta &= \big\langle\!\big\langle \{q\}, \mathrm{supp}(E^X)\big\rangle\!\big\rangle \oplus \big\langle\!\big\langle \mathrm{supp}(E^X) \oplus m^*, \mathcal{L}_Z^*\big\rangle\!\big\rangle \end{aligned} \tag{32}$$

where $m \subset \mathsf{E}_{\mathrm{dec}}$ and $m^* \subset \mathsf{E}_{\mathrm{dec}^*}$ are the minimum matchings computed in Step 3 of the algorithm.

According to Lemma 3.1, the post-measurement state after Step 1 of Algorithm 1 is the Bell state $\big|\Phi_{(c_X,c_Z)}\big\rangle$ with

$$\begin{aligned} c_X &= \langle\!\langle x, \mathcal{L}_X\rangle\!\rangle \oplus \mathsf{Syn}(S^X, E) \\ c_Z &= \langle\!\langle z, \mathcal{L}_Z^*\rangle\!\rangle \oplus \mathsf{Syn}(S^Z, E)\ . \end{aligned}$$

Since the algorithm applies the correction operation $Z^{\hat{c}_X} X^{\hat{c}_Z}$ in Step 6, the output of the algorithm is the Bell state $\big|\Psi_{(c_X \oplus \hat{c}_X, c_Z \oplus \hat{c}_Z)}\big\rangle$. It follows with the definition of $(\hat{c}_X, \hat{c}_Z)$ in Step 5, i.e.,

$$\begin{aligned} \hat{c}_X &= \langle\!\langle x, \mathcal{L}_X\rangle\!\rangle \oplus \hat{s}^X \\ \hat{c}_Z &= \langle\!\langle z, \mathcal{L}_Z^*\rangle\!\rangle \oplus \hat{s}^Z \end{aligned}$$

that the output is the Bell state $\big|\Phi_{(\alpha,\beta)}\big\rangle$ where

$$\begin{aligned} \alpha &= \hat{s}^X \oplus \mathsf{Syn}(S^X, E) \qquad \text{and} \\ \beta &= \hat{s}^Z \oplus \mathsf{Syn}(S^Z, E)\ . \end{aligned}$$

The claim (32) now follows by combining the expressions (19), (20) for $\big(\mathsf{Syn}(S^X, E), \mathsf{Syn}(S^Z, E)\big)$ from Lemma 3.2 with the Definitions (29) of $(\hat{s}^X, \hat{s}^Z)$ used in Step 4 of the algorithm.

The claim of the theorem now follows by combining (32) with the fact that

$$\begin{aligned} m &= \mathsf{MinMatch}(s) = \mathsf{MinMatch}(\partial \mathrm{supp}(E^Z)) \\ m^* &= \mathsf{MinMatch}^*(s^*) = \mathsf{MinMatch}^*(\partial^* \mathrm{supp}(E^X)) \end{aligned}$$

by the definition of (m, m^*), and the expressions for (s, s^*) established in Lemma 3.3. □

3.2 Bounds on the resilience functions for $\mathsf{T}_{\mathrm{dec}}$ and $\mathsf{T}_{\mathrm{dec}^*}$

Here we establish the following bounds on the resilience functions of interest:

Lemma 3.5. *Consider the graph $\mathsf{T}_{\mathrm{dec}}$ with internal vertices $\mathsf{V}_{\mathrm{dec}}^{\mathrm{int}}$ defined by* (3)*, see Fig. 3a. Let $\mathcal{L}_X \subset \mathsf{E}_{\mathrm{dec}}$ be the subset of edges on the vertical strip in Fig. 3a (cf.* (1)*). Then the resilience function $\mathsf{res}_{\mathcal{L}_X}$ satisfies*

$$\mathsf{res}_{\mathcal{L}_X}(p) \leq 54p \tag{33}$$

for any $p \in [0, \frac{1}{144}]$.

Proof. Consider a simple closed loop $L \in Z_\circ(\mathsf{T}_{\text{dec}})$. Clearly, the definition of $\mathcal{L}_X$ implies that L and $\mathcal{L}_X$ have no overlap. In particular, $\langle\!\langle L, \mathcal{L}_X \rangle\!\rangle = 0$, implying that closed loops do not contribute to the resilience function $\mathsf{res}_{\mathcal{L}_X}$ in this case.

To describe the set $Z_{\text{ext}}(\mathsf{T}_{\text{dec}})$ of simple paths with endpoints in $\mathsf{V}_{\text{dec}}^{\text{ext}}$ through internal vertices, we label the external vertices in $\mathsf{V}_{\text{dec}}^{\text{ext}}$ as

$$\mathsf{V}_{\text{dec}}^{\text{ext}} = \{u_1, \ldots, u_{d-1}, v_1, \ldots, v_{d-1}\} \ ,$$

see Fig. 3a. Explicitly, the external vertices are

$$u_j = (0, 2j), \quad v_j = (2, 2j) \qquad \text{for} \qquad j = \{1, \ldots, d-1\} \ .$$

Clearly, a simple path $P \in Z_{\text{ext}}(\mathsf{T}_{\text{dec}})$ with endpoints in $\mathsf{V}_{\text{dec}}^{\text{ext}}$ through internal vertices satisfies $\langle\!\langle P, \mathcal{L}_X \rangle\!\rangle = 1$ if and only if P starts at u_j (with $j \in \{1, \ldots, d-1\}$) and ends at v_k (with $k \in \{1, \ldots, d-1\}$). For $j \in \{1, \ldots, d-1\}$ and $\ell \in \mathbb{N}$, let $\Delta(u_j, \ell)$ be the set of all such paths $P \in Z_{\text{ext}}(\mathsf{T}_{\text{dec}})$ of length ℓ that start at u_j and end at some vertex in the set $\{v_k\}_{k=1}^{d-1}$. Then we have

$$\begin{aligned}
\mathsf{res}_{\mathcal{L}_X}(p) &= \sum_{\ell=1}^{\infty} \binom{\ell}{\lceil \ell/2 \rceil} \cdot \Big| \bigcup_{j=1}^{d-1} \Delta(u_j, \ell) \Big| \cdot p^{\lceil \ell/2 \rceil} \\
&= \sum_{j=1}^{d-1} \sum_{\ell=L_j^{\min}}^{L_j^{\max}} \binom{\ell}{\lceil \ell/2 \rceil} \cdot |\Delta(u_j, \ell)| \cdot p^{\lceil \ell/2 \rceil} \ . \qquad (34)
\end{aligned}$$

because $\Delta(u_j, \ell)$ and $\Delta(u_{j'}, \ell)$ are disjoint for $j \neq j'$ since correspondings paths have distinct starting points $u_j \neq u_{j'}$. Here $L_j^{\min}$ and $L_j^{\max}$ are the minimal and maximal lengths of a simple path $P \in Z_{\text{ext}}(\mathsf{T}_{\text{dec}})$ starting at u_j and ending in $\{v_k\}_{k=1}^{d-1}$ without touching any other external vertices.

The set $\Delta(u_1, 1)$ consists of a single path, i.e., $|\Delta(u_1, 1)| = 1$. For $j \in \{2, \ldots, d-1\}$, we use that the graph T_{dec} has vertices of degree at most 4, which implies that

$$|\Delta(u_j, \ell)| \leq 3^{\ell-1} \qquad \text{for all} \qquad \ell \in \mathbb{N} \ .$$

This is because any path $P \in \Delta(u_j, \ell)$ has the same first edge (connected to u_j), and there are at most 3 choices for each of the remaining edges.) Furthermore, it is clear that

$$L_j^{\min} \geq j \ .$$

We conclude (where for $\ell > 1$, we use the inequalities $p^{\lceil \ell/2 \rceil} \leq p^{\ell/2}$ and $\binom{\ell}{\lceil \ell/2 \rceil} \leq 2^\ell$) that

$$\begin{aligned}
\mathsf{res}_{\mathcal{L}_X}(p) &\leq p + \sum_{j=2}^{d-1} \sum_{\ell=j}^{L_j^{\max}} 2^\ell 3^{\ell-1} p^{\ell/2} \\
&\leq p + \frac{1}{3} \sum_{j=2}^{d-1} \sum_{\ell=j}^{\infty} q^\ell \qquad \text{where} \qquad q := 6\sqrt{p} \\
&= \frac{q^2}{6} + \frac{q^2 - q^d}{3(1-q)^2} \\
&\leq p + \frac{4}{3}\left(q^2 - q^d\right) \\
&\leq p + \frac{4}{3} \cdot (6\sqrt{p})^2 \\
&\leq 54p \ .
\end{aligned}$$

Here we used the fact $q \leq 6\sqrt{\frac{1}{144}} = \frac{1}{2}$ and that $\frac{1}{1-q} \leq 2$. □

A similar bound applies to the dual decoding graph $\mathsf{T}_{\mathrm{dec}^*}$.

Lemma 3.6. *Consider the dual graph $\mathsf{T}_{\mathrm{dec}^*}$ with internal vertices $\mathsf{V}^{\mathrm{int}}_{\mathrm{dec}^*}$ defined by* (4)*, see Fig. 3b. Let $\mathcal{L}_Z^* \subset \mathsf{E}_{\mathrm{dec}^*}$ be the subset of edges on the horizontal strip in Fig. 3b (cf.* (2)*). Then the resilience function $\mathsf{res}_{\mathcal{L}_Z^*}$ satisfies*

$$\mathsf{res}_{\mathcal{L}_Z^*}(p) \leq 38p$$

for any $p \in [0, \frac{1}{144}]$.

The proof is analogous to that of Lemma 3.5. We include it in Appendix B for completeness.

3.3 A bound on single-shot decoding under local stochastic noise

Consider a distance-d surface code of $|\mathcal{C}| = 2d^2 - 2d + 1$ qubits with $d \geq 2$ and logical operators $\overline{X} = \prod_{j \in \mathcal{L}_X \cup \{q\}} X_j$ and $\overline{Z} = \prod_{j \in \mathcal{L}_Z \cup \{q\}}$. Let $|\overline{0}\rangle$ and $|\overline{1}\rangle$ be code states to eigenvalues $+1$ and -1 of $\overline{Z}$. Let

$$\begin{array}{cccc} V: & \mathbb{C}^2 & \mapsto & (\mathbb{C}^2)^{\otimes \mathcal{C}} \\ & \alpha|0\rangle + \beta|1\rangle & \mapsto & \alpha|\overline{0}\rangle + \beta|\overline{1}\rangle \end{array}$$

be an isometric encoding map, and let

$$\begin{array}{cccc} \mathcal{E}: & \mathcal{B}(\mathbb{C}^2) & \to & \mathcal{B}((\mathbb{C}^2)^{\otimes \mathcal{C}}) \\ & \rho & \mapsto & V\rho V^\dagger \end{array}$$

be an encoding channel associated with V. For any state $\rho \in \mathcal{B}(\mathbb{C}^2)$, the state $\mathcal{E}(\rho)$ is the corresponding encoded (logical) state.

We describe what single-shot decoding protocol achieves in terms of its logical action. For this purpose, let us define the single-qubit random Pauli channel

$$\mathcal{P}_\nu(\rho) := \nu_0 \rho + \nu_1 X\rho X^\dagger + \nu_2 Y\rho Y^\dagger + \nu_3 Z\rho Z^\dagger \ .$$

for any probability distribution $\nu = (\nu_0, \nu_1, \nu_2, \nu_3)$, $\nu_j \geq 0$ and $\sum_{j=0}^3 \nu_j = 1$.

For $p \in [0, 1]$, let $E \sim \mathcal{N}(p)$ be a local stochastic error with parameter p. This defines a "noise channel" Λ_p by

$$\begin{array}{cccc} \Lambda_p: & \mathcal{B}((\mathbb{C}^2)^{\otimes \mathcal{C}}) & \to & \mathcal{B}((\mathbb{C}^2)^{\otimes \mathcal{C}}) \\ & \rho & \mapsto & \Lambda_p(\rho) := \sum_{E_0 \text{ Pauli on } (\mathbb{C}^2)^{\otimes \mathcal{C}}} \Pr[E = E_0]\, E_0 \rho E_0^\dagger \end{array} \tag{35}$$

be the CPTP map that applies the error E to a state. Finally, let $\mathcal{D}: \mathcal{B}((\mathbb{C}^2)^{\otimes \mathcal{C}}) \to \mathcal{B}((\mathbb{C}^2)^{\{q\}})$ be the CPTP map corresponding to an ideal execution of Algorithm 1. Then the following holds:

Theorem 3.7 (Single-shot decoding of a surface code)**.** *Consider local stochastic noise as expressed by a CPTP map Λ_p of the form* (35)*, with noise strength*

$$p \leq \frac{1}{144} \ .$$

There is a probability distribution $\nu = (\nu_0, \nu_1, \nu_2, \nu_3)$ over (I, X, Y, Z) such that the following holds:

(i) Suppose we run algorithm 1 on the noisy encoded state $\Lambda_p \circ \mathcal{E}(\rho)$, where $\rho \in \mathcal{B}(\mathbb{C}^2)$ is an arbitrary single-qubit state. Then the resulting output state $\mathcal{D} \circ \Lambda_p \circ \mathcal{E}(\rho)$ on qubit q is the state $\mathcal{P}_\nu(\rho)$.

(ii) We have $1 - \nu_0 \leq 94p$.

In particular, Theorem 3.7 implies that applying Algorithm 1 to any encoded pure state $\Lambda_p(V|\Psi\rangle\langle\Psi|V^\dagger)$ corrupted by Λ_p yields an output state $\rho_{\text{out}} = \mathcal{D}\circ\Lambda_p\circ\mathcal{E}(|\Psi\rangle\langle\Psi|)$ with overlap at least

$$\langle\Psi|\,\rho_{\text{out}}\,|\Psi\rangle \geq \nu_0 \geq 1-94p$$

with the original state.

Proof. Let us denote by $L\cong\mathbb{C}^2$ the "logical qubit" to be encoded, and let $R\cong\mathbb{C}^2$ be a reference system. We consider the action of the noise and the decoding protocol when applied to the two-qubit Bell state $\Phi_{RL} := \Phi_{(0,0)}$, i.e., we compute the Choi-Jamiolkowski state

$$J_{Rq} := (\mathsf{id}_R\otimes\mathcal{D}\circ\Lambda_p\circ\mathcal{E})(\Phi_{RL})\ .$$

Observe that $\Phi'_{R\mathcal{C}} := (\mathsf{id}_R\otimes\mathcal{E})(\Phi_{RL})$ is the half-encoded Bell state by definition. It thus follows from Theorem 3.4 that

$$J_{Rq} := (\mathsf{id}_R\otimes\mathcal{P}_\nu)(\left|\Phi_{(0,0)}\right\rangle\left\langle\Phi_{(0,0)}\right|) \tag{36}$$

for a probability distribution $\nu = (\nu_0,\nu_1,\nu_2,\nu_3)$ over (I,X,Y,Z). In this expression, ν_0 is the probability that the condition (31) is satisfied. Because of the Choi-Jamiolkowski isomorphism, Eq. (36) shows that the channel $\mathcal{D}\circ\Lambda_p\circ\mathcal{E} = \mathcal{P}_\nu$ is a random Pauli channel. It thus remains to establish an upper bound on $1-\nu_0$.

Let us introduce the following four random variables:

$$\begin{aligned}
A_Z &:= \langle\!\langle \{q\}, \mathrm{supp}(E^Z)\rangle\!\rangle\\
B_Z &= \langle\!\langle \mathrm{supp}(E^Z)\oplus \mathsf{MinMatch}(\partial\mathrm{supp}(E^Z)), \mathcal{L}_X\rangle\!\rangle\\
A_X &:= \langle\!\langle \{q\}, \mathrm{supp}(E^X)\rangle\!\rangle\\
B_X &:= \langle\!\langle \mathrm{supp}(E^X)\oplus \mathsf{MinMatch}^*(\partial\mathrm{supp}(E^X)), \mathcal{L}^*_Z\rangle\!\rangle\ .
\end{aligned}$$

Then (31) translates to

$$\nu_0 = \Pr\left[(A_Z,B_Z)\in\{(0,0),(1,1)\}\text{ and }(A_X,B_X)\in\{(0,0),(1,1)\}\right]\ .$$

The union bound gives

$$1-\nu_0 \leq \Pr\left[(A_Z,B_Z)\in\{(0,1),(1,0)\}\right] + \Pr\left[(A_X,B_X)\in\{(0,1),(1,0)\}\right]\ .$$

Again using the union bound, we have

$$\begin{aligned}
\Pr\left[(A_Z,B_Z)\in\{(0,1),(1,0)\}\right] &\leq \Pr\left[(A_Z,B_Z)=(0,1)\right] + \Pr\left[(A_Z,B_Z)=(1,0)\right]\\
&\leq \Pr\left[B_Z=1\right] + \Pr\left[A_Z=1\right]\ .
\end{aligned}$$

Applying the same reasoning to (A_X,B_X), we conclude that

$$1-\nu_0 \leq \Pr\left[A_X=1\right] + \Pr\left[A_Z=1\right] + \Pr\left[B_X=1\right] + \Pr\left[B_Z=1\right]\ .$$

By definition, we have

$$\begin{aligned}
\Pr\left[A_Z=1\right] &= \Pr\left[\langle\!\langle \{q\},\mathrm{supp}(E^Z)\rangle\!\rangle = 1\right]\\
&\leq p
\end{aligned}$$

where we used that $E^Z\sim\mathcal{N}(p)$ is local stochastic noise with the same parameter p as E. By similar reasoning, we have

$$\Pr\left[A_X=1\right]\leq p\ .$$

Furthermore, we have

$$\begin{aligned}\Pr\left[B_Z = 1\right] &= \Pr\left[\langle\!\langle \mathrm{supp}(E^Z) \oplus \mathsf{MinMatch}(\partial \mathrm{supp}(E^Z)), \mathcal{L}_X \rangle\!\rangle = 1\right] \\ &\leq \mathsf{res}_{\mathcal{L}_X}(p) \\ &\leq 54p\end{aligned}$$

where we used the definition of A_Z, the fact that $E^Z \sim \mathcal{N}(p)$ and Proposition 2.3, as well as the upper bound (33). Similarly, we have

$$\begin{aligned}\Pr\left[B_X = 1\right] &\leq \mathsf{res}_{\mathcal{L}_Z^*}(p) \\ &\leq 38p\ .\end{aligned}$$

Combining these inequalities, we obtain

$$\begin{aligned}1 - \nu_0 &\leq p + p + 38p + 54p \\ &\leq 94p\end{aligned}$$

as claimed. □

4 Entanglement generation from a noisy 3D cluster state

In the following, we describe a protocol for generating a two-qubit Bell state in a noise-robust fashion. It is based on the cluster state $W\left|0^{\mathcal{C}}\right\rangle$ on a 3D lattice $\mathcal{C}$, a state which can be generated by a depth-6 circuit W. This state is measured using single-qubit measurements on all but two qubits. Based on the measurement outcomes, a certain correction operation is applied to one of the remaining two qubits.

In Section 4.1 we discuss the relevant definitions underlying the cluster state. In Section 4.2 we describe the single-qubit measurement pattern used in the protocol. Section 4.3 introduces certain subgraphs of the cluster state lattice and additional data such as distinguished subsets of qubits having to do with the encoded logical information. These are relevant for the definition of certain decoding functions used in the protocol.

Section 4.4 identifies certain stabilizers of the graph state that are key to the construction and analysis of the entanglement generation protocol. We give a complete description of the protocol in Section 4.5. Finally, in Section 4.6, we compute the output state of this algorithm when it is run on a noisy cluster state $EW\left|0^{\mathcal{C}}\right\rangle$ which is corrupted by a Pauli error E. This will be used to analyze the success probability for local stochastic errors in Section 5.

4.1 Cluster state: Definition

Our construction is based on a 3D cluster state. To introduce this state, we mostly follow the conventions of [1] but use different conventions to describe so-called dangling edges. Contrary to Ref. [1] we will consider their degree-1 endpoints as regular vertices.

Let $d \geq 2$ and let $R \geq 3$ be an odd integer. Consider the set

$$\widetilde{\mathcal{C}}[d \times d \times R] := \{(u_1, u_2, u_3) \in \mathbb{Z}^3 \mid 0 \leq u_1 \leq 2d,\ 0 \leq u_2 \leq 2d-2,\ 1 \leq u_3 \leq R\}$$

(From the perspective of coding, the integers d and R are associated with the distance d of a surface code, and a "cluster state distance" $\frac{R+1}{2}$, respectively.) In the following, we simply write $\widetilde{\mathcal{C}} = \widetilde{\mathcal{C}}[d \times d \times R]$. Elements $(u_1, u_2, u_3) \in \widetilde{\mathcal{C}}$ will be referred to as sites.

Qubits are located at each site belonging to the subset

$$\mathcal{C}[d \times d \times R] := \{u \in \widetilde{\mathcal{C}} \mid 1 \leq u_1 \leq 2d-1\} \setminus \{(o,o,o),(e,e,e)\} \subset \widetilde{\mathcal{C}}$$

where each e (o) refers to any even (odd) integer. The set $\mathcal{C} = \mathcal{C}[d \times d \times R]$ is called the cluster state lattice of the form $d \times d \times R$. We will refer to a site $u \in \mathcal{C}$ interchangeably as site u or qubit u. In the following, we use the convention that e.g., $\{(e, e, R)\} \subset \widetilde{\mathcal{C}}$ denotes the set of all $(u_1, u_2, u_3) \in \widetilde{\mathcal{C}}$ with u_1, u_2 even and $u_3 = R$.

Given a site $u \in \widetilde{\mathcal{C}}$, the set of nearest neighbors of u is defined as

$$\text{neigh}(u) = \{v \in \mathcal{C} \mid d(u, v) = 1\} \qquad \text{where} \qquad d(u, v) := \sum_{i=1}^{3} |u_i - v_i| \ , \tag{37}$$

i.e., the nearest neighbors of u are associated with qubits at Manhattan distance 1 from u. Because elements of $\widetilde{\mathcal{C}} \setminus \mathcal{C}$ are excluded in (37), any qubit $u \in \mathcal{C}$ has at most four nearest neighbor qubits.

The cluster state of interest is associated with the graph whose vertex set is $\mathcal{C}$, and where the set of edges consists of pairs $\{u, v\}$ with $v \in \text{neigh}(u)$, see Fig. 4 for an illustration of this graph. It is defined as

$$W \left|0^{\mathcal{C}}\right\rangle \ ,$$

where

$$W = H(\mathcal{C}) \left(\prod_{\{u,v\}} CZ_{u,v} \right) H(\mathcal{C}) \ ,$$

with the product taken over all edges of the graph, i.e., all pairs $u, v \in \mathcal{C}$ such that $v \in \text{neigh}(u)$. Here $CZ_{u,v}$ denotes a controlled-Z gate on qubits u and v. By definition, the unitary W can be implemented using a depth-6 circuit with nearest-neighbor gates. (Note that our definition of the graph state differs from the more commonly used definition by an additional layer of Hadamard-gates.) The state $W \left|0^{\mathcal{C}}\right\rangle$ is a stabilizer state with stabilizer group generated by

$$G_u = Z_u \prod_{v \in \text{neigh}(u)} X_v \qquad \text{with} \qquad u \in \mathcal{C} \ . \tag{38}$$

As in the case of surface codes, it will also need a dual description. For this, let $\widetilde{\mathcal{C}}^* = \widetilde{\mathcal{C}}^*[d \times d \times R]$ be the set of dual sites defined as

$$\widetilde{\mathcal{C}}^*[d \times d \times R] := \left\{(u_1, u_2, u_3) \in \mathbb{Z}^3 \mid 1 \leq u_1 \leq 2d - 1 \text{ and } -1 \leq u_2 \leq 2d - 1 \text{ and } 1 \leq u_3 \leq R\right\} \ .$$

Then we can equivalently express the location of qubits as

$$\mathcal{C}^* = \{u \in \widetilde{\mathcal{C}}^* \mid 0 \leq u_2 \leq r - 1\} \setminus \{(o, o, o), (e, e, e)\} \subset \widetilde{\mathcal{C}}^* \ ,$$

i.e., we have $\mathcal{C} = \mathcal{C}^*$.

4.2 Measurement pattern used in protocol

In this section, we describe the measurement pattern used in our protocol. We partition the set of qubits as $\mathcal{C} = \mathcal{A} \cup \mathcal{B}$ where

$$\begin{aligned} \mathcal{B} &= \{(e, o, 1), (o, e, 1), (e, o, R), (o, e, R) \in \mathcal{C}\}, \\ \mathcal{A} &= \mathcal{C} \setminus \mathcal{B}. \end{aligned}$$

The set $\mathcal{B}$ is the union of $\{(e, o, 1), (o, e, 1)\}$, i.e., a subset of all qubits at the "front" layer (i.e., with $u_3 = 1$) of $\mathcal{C}$, and $\{(e, o, R), (o, e, R)\}$, a subset of all qubits at the "back" layer (i.e., with $u_3 = R$) of $\mathcal{C}$. In prior work [11, 1], no measurements were applied to these qubits; instead, long-range encoded

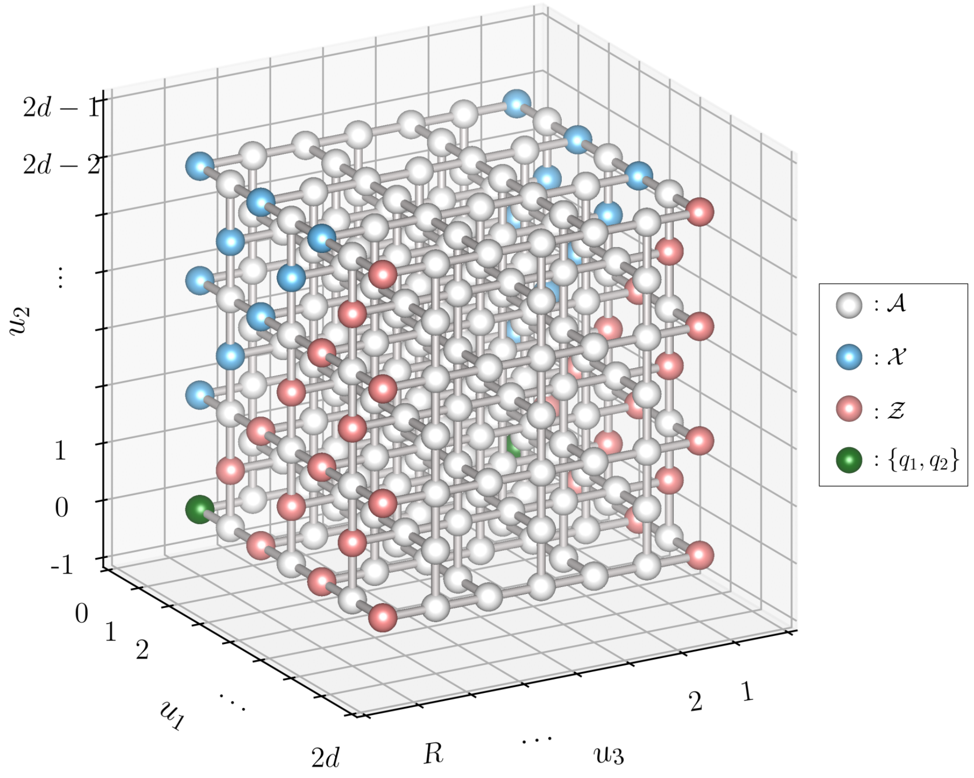

Figure 4: The graph associated with the three-dimensional cluster state $W\left|0^{\mathcal{C}}\right\rangle$. Each qubit is located at a vertex (sphere) in $\mathcal{C}$. Measurement pattern used in protocol is illustrated with colors.

entanglement between two surface codes of distance d associated with these front- and back-layer qubits was established.

The remaining qubits $\{(e, e, 1)\}$ in the front layer are omitted from $\mathcal{B}$ but belong to $\mathcal{A}$. Similarly, qubits $\{(e, e, R)\}$ in the back layer belong to $\mathcal{A}$. These are auxiliary qubits whose measurement (specified below) reveals information about the associated surface code stabilizers in the corresponding layer. Similarly, the measurement results associated with the remaining qubits of $\mathcal{A}$ belonging to the "bulk" reveal information about the logical error of the encoded surface code state (or, in our scheme, the final two-qubit entangled state).

As in [11, 1], all qubits belonging to $\mathcal{A}$ are measured in the Z-basis. Because our goal is to establish entanglement between two (physical) qubits, we additionally measure all qubits in $\mathcal{B}$, except for two qubits

$$q_1 := (1, 0, 1)$$
$$q_2 := (1, 0, R)$$

belonging to the front and back layers, respectively. Our protocol generates a Bell state between these two qubits.

The measurement pattern for the front layer qubits of $\mathcal{B}$ mirrors the single-shot decoding procedure for the surface code: Qubits belonging to the upper left triangular region are measured in the X-basis, whereas qubits situated inside the lower right triangular region are measured in the Z-basis. Qubits in the back layer of $\mathcal{B}$ are measured with an analogous pattern. Formally, the sets of qubits in $\mathcal{B} \setminus \{q_1, q_2\}$ measured in the X- respectively Z-basis are

$$\mathcal{X} = \{(u_1, u_2, u_3) \in \mathcal{B} \setminus \{q_1, q_2\} \mid u_2 \geq 1 + u_1\}$$
$$\mathcal{Z} = \{(u_1, u_2, u_3) \in \mathcal{B} \setminus \{q_1, q_2\} \mid u_2 < 1 + u_1\} .$$

The measurement outcome will be denoted by (a, x, z), where

$$a \in \{0,1\}^{\mathcal{A}}$$
$$x \in \{0,1\}^{\mathcal{X}}$$
$$z \in \{0,1\}^{\mathcal{Z}}$$

are the measurement results associated with the qubits belonging to $\mathcal{A}$, $\mathcal{X}$ and $\mathcal{Z}$, respectively. This completes the description of the measurements used in the protocol, see Fig. 4.

4.3 Subgraphs, recovery graphs and internal vertices

In this section, we introduce various subgraph of the cluster state lattice. We also define certain subsets of qubits that are relevant for our entanglement generation protocol.

Specifically, in Section 4.3.1, we introduce the "glued graph" T_{gl}, as well as the "dual glued graph" $\mathsf{T}^*_{\mathrm{gl}}$. They are obtained by gluing together surface code graphs respectively dual surface code graphs with the cluster state lattice. These graphs are at the basis of our entanglement generation protocol, and clarify the relationship to the single-shot decoding procedure for the surface code discussed in Section 1.

In Section 4.3.2, we then introduce the decoding graph $\mathsf{T}_{\mathrm{cl,dec}}$ and its dual counterpart $\mathsf{T}_{\mathrm{cl,dec}^*}$. In Section 4.3.3, we additionally define certain subsets of their edges, the so-called recovery sets. We also identify the associated internal vertices. Our entanglement generation protocol involves solving corresponding matching problems.

4.3.1 Glued graphs

We introduce certain graphs in $\widetilde{\mathcal{C}}$ and their dual in $\widetilde{\mathcal{C}}^*$ as discussed in [1]. Those graphs are subgraphs or supergraphs of certain graphs that will be introduced in Section 4.3.2.

We first define an "even graph" $\mathsf{T}_{\mathrm{even}} = (\mathsf{V}_{\mathrm{even}}, \mathsf{E}_{\mathrm{even}})$ in $\widetilde{\mathcal{C}}$. The set $\mathsf{V}_{\mathrm{even}} = \mathsf{V}^\circ_{\mathrm{even}} \cup \mathsf{V}'_{\mathrm{even}}$ of vertices has two disjoint subsets

$$\mathsf{V}^\circ_{\mathrm{even}} = \{u \in \{(e,e,e)\} \mid 2 \leq u_2 \leq 2d-2\} \subset \widetilde{\mathcal{C}} \qquad \text{and}$$
$$\mathsf{V}'_{\mathrm{even}} = \{u \in \{(e,e,1),(e,e,R)\} \mid u_1 \notin \{0,2d\}\} \cup \{(0,e,e)\} \cup \{(2d,e,e)\} \subset \widetilde{\mathcal{C}}\ .$$

Here we avoid the terminology of dangling edges used in [1] and instead collect endpoints of such edges using the vertex set $\mathsf{V}'_{\mathrm{even}}$. We note that $\mathsf{V}'_{\mathrm{even}}$ consists of vertices $u = (u_1, u_2, u_3)$ in the "front" ($u_3 = 1$), "back" ($u_3 = R$), "left" ($u_1 = 0$), or "right" ($u_1 = 2d$) boundary. Any pair $u, v \in \mathsf{V}_{\mathrm{even}}$ of vertices at distance $d(u, v) = 2$ is connected by an edge in $\mathsf{T}_{\mathrm{even}}$, except when u, v both belong to $\mathsf{V}'_{\mathrm{even}}$. Other edges in $\mathsf{T}_{\mathrm{even}}$ are of the form $\{u, v\}$ with

$$u = (u_1,u_2,u_3) \in \{(e,e,1),(e,e,R)\} \qquad \text{and} \qquad v = \begin{cases} (u_1,u_2,u_3+1) & \text{if } u_3 = 1 \\ (u_1,u_2,u_3-1) & \text{if } u_3 = R\ . \end{cases} \tag{39}$$

It is easy to check that these edges constitute all pairs of vertices $u, v \in \mathsf{V}_{\mathrm{even}}$ satisfying $d(u, v) = 1$. In summary, the set of edges of $\mathsf{T}_{\mathrm{even}}$ is

$$\begin{aligned}\mathsf{E}_{\mathrm{even}} = &\left\{\{u,v\} \in \mathsf{V}^2_{\mathrm{even}} \mid d(u,v) = 2 \text{ and } (u,v) \notin \mathsf{V}'_{\mathrm{even}} \times \mathsf{V}'_{\mathrm{even}}\right\} \\ &\cup \left\{\{u,v\} \in \mathsf{V}^2_{\mathrm{even}} \mid d(u,v) = 1\right\}\ .\end{aligned}$$

The "odd graph" $\mathsf{T}_{\mathrm{odd}} = (\mathsf{V}_{\mathrm{odd}}, \mathsf{E}_{\mathrm{odd}})$ in $\widetilde{\mathcal{C}}^*$ is a dual version of $\mathsf{T}_{\mathrm{even}}$. The set $\mathsf{V}_{\mathrm{odd}} = \mathsf{V}^\circ_{\mathrm{odd}} \cup \mathsf{V}'_{\mathrm{odd}}$ of vertices consists of two disjoint subsets

$$\mathsf{V}^\circ_{\mathrm{odd}} = \{u \in \{(o,o,o)\} \mid u_2 \notin \{-1,r\}, u_3 \notin \{1,R\}\} \subset \widetilde{\mathcal{C}}^* \qquad \text{and}$$
$$\mathsf{V}'_{\mathrm{odd}} = \{u \in \{(o,o,1),(o,o,R)\} \mid u_2 \notin \{-1,r\}\} \cup \{u \in \{(o,-1,o),(o,r,o)\} \mid u_3 \notin \{1,R\}\} \subset \widetilde{\mathcal{C}}^*\ .$$

We note that V'_{odd} consists of vertices $u = (u_1, u_2, u_3)$ in the "front" ($u_3 = 1$), "back" ($u_3 = R$), "top" ($u_2 = 2d$), or "bottom" ($u_2 = -1$) boundary. The set of edges of T_{odd} is

$$\mathsf{E}_{\text{odd}} = \{\{u, v\} \in \mathsf{V}^2_{\text{odd}} \mid d(u, v) = 2 \text{ and } (u, v) \notin \mathsf{V}'_{\text{odd}} \times \mathsf{V}'_{\text{odd}}\} \ .$$

The surface code graph $\mathsf{T}_{\text{cl,sc}}$ has vertices belonging to the set of primal sites $\widetilde{\mathcal{C}}$. It has two connected components lying in the planes $u_3 = 1$ and $u_3 = R$. Each connected component is isomorphic to the surface code graph $\mathsf{T}_{\text{sc}} = (\mathsf{V}_{\text{sc}}, \mathsf{E}_{\text{sc}})$ introduced in Section 1.1. Let us simply write

$$\mathsf{T}_{\text{cl,sc}} = \mathsf{T}_{\text{sc}} \times \{1, R\}$$

for this graph to emphasize this fact. In more detail, we have

$$\begin{aligned}
\mathsf{V}_{\text{cl,sc}} &= \left(\bigcup_{(u_1,u_2)\in \mathsf{V}_{\text{sc}}} \{(u_1, u_2, 1)\}\right) \cup \left(\bigcup_{(u_1,u_2)\in \mathsf{V}_{\text{sc}}} \{(u_1, u_2, R)\}\right)\\
\mathsf{E}_{\text{cl,sc}} &= \left(\bigcup_{\{(u_1,\,u_2),\,(v_1,\,v_2)\}\,\in\,\mathsf{E}_{\text{sc}}} \Big\{\{(u_1, u_2, 1), (v_1, v_2, 1)\}\Big\}\right) \cup \left(\bigcup_{\{(u_1,\,u_2),\,(v_1,\,v_2)\}\,\in\,\mathsf{E}_{\text{sc}}} \Big\{\{(u_1, u_2, R), (v_1, v_2, R)\}\Big\}\right) \ .
\end{aligned}$$

The graph $\mathsf{T}_{\text{cl,sc}^*}$ similarly consists of two copies of the dual surface code graph T_{sc} located in the planes $u_3 = 1$ and $u_3 = R$, respectively, which we express as

$$\mathsf{T}_{\text{cl,sc}^*} = \mathsf{T}_{\text{sc}^*} \times \{1, R\} \ .$$

Written out, this means that

$$\begin{aligned}
\mathsf{V}_{\text{cl,sc}^*} &= \left(\bigcup_{(u_1,u_2)\in \mathsf{V}_{\text{sc}^*}} \{(u_1, u_2, 1)\}\right) \cup \left(\bigcup_{(u_1,u_2)\in \mathsf{V}_{\text{sc}^*}} \{(u_1, u_2, R)\}\right)\\
\mathsf{E}_{\text{cl,sc}^*} &= \left(\bigcup_{\{(u_1,\,u_2),\,(v_1,\,v_2)\}\,\in\,\mathsf{E}_{\text{sc}^*}} \{(u_1, u_2, 1), (v_1, v_2, 1)\}\right) \cup \left(\bigcup_{\{(u_1,\,u_2),\,(v_1,\,v_2)\}\,\in\,\mathsf{E}_{\text{sc}^*}} \{(u_1, u_2, R), (v_1, v_2, R)\}\right) \ .
\end{aligned}$$

Now we are able to introduce a certain "glued graph" $\mathsf{T}_{\text{gl}} = (\mathsf{V}_{\text{gl}}, \mathsf{E}_{\text{gl}})$, as well as a "dual glued graph" $\mathsf{T}^*_{\text{gl}} = (\mathsf{V}^*_{\text{gl}}, \mathsf{E}^*_{\text{gl}})$, in terms of the subgraphs T_{even}, $\mathsf{T}_{\text{cl,sc}}$, T_{odd}, and $\mathsf{T}_{\text{cl,sc}^*}$, see Fig. 5.

The glued graph T_{gl} is obtained by attaching the surface graph $\mathsf{T}_{\text{cl,sc}}$ to the even graph T_{even}; here the two copies of the surface code end up being connected to the rough front- and back boundaries of the even graph T_{even}. The graph T_{gl} is formally defined by setting

$$\begin{aligned}
\mathsf{V}_{\text{gl}} &= \mathsf{V}_{\text{even}} \cup \mathsf{V}_{\text{cl,sc}}\\
\mathsf{E}_{\text{gl}} &= \mathsf{E}_{\text{even}} \cup \mathsf{E}_{\text{cl,sc}} \ .
\end{aligned}$$

We denote this construction simply by

$$\mathsf{T}_{\text{gl}} = \mathsf{T}_{\text{even}} \cup \mathsf{T}_{\text{cl,sc}} \ .$$

The "dual glued graph" $\mathsf{T}^*_{\text{gl}} = (\mathsf{V}^*_{\text{gl}}, \mathsf{E}^*_{\text{gl}})$ is defined by gluing the dual surface graph $\mathsf{T}_{\text{cl,sc}}$ to the odd graph T_{odd}, i.e.,

$$\mathsf{T}^*_{\text{gl}} = \mathsf{T}_{\text{odd}} \cup \mathsf{T}_{\text{cl,sc}^*} \ .$$

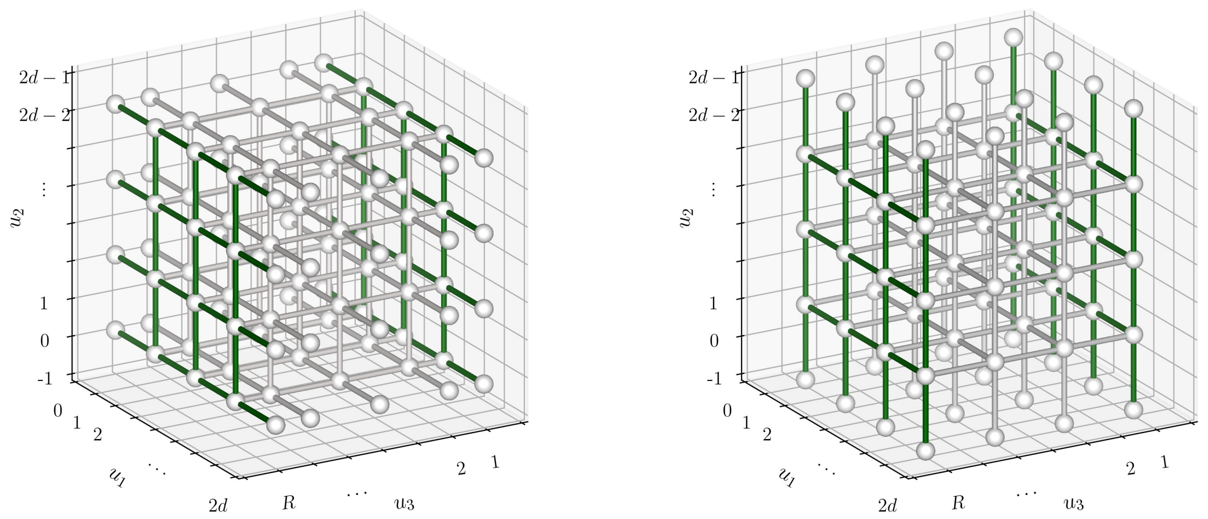

(a) The glued graph T_{gl} as a union of two subgraphs: $\mathsf{T}_{\mathrm{even}}$ (gray) and $\mathsf{T}_{\mathrm{cl,sc}}$ (green).

(b) The dual glued graph $\mathsf{T}^*_{\mathrm{gl}}$ as a union of two subgraphs: $\mathsf{T}_{\mathrm{odd}}$ (gray) and $\mathsf{T}_{\mathrm{cl,sc}^*}$ (green).

Figure 5: The glued graph $\mathsf{T}_{\mathrm{gl}} = \mathsf{T}_{\mathrm{even}} \cup \mathsf{T}_{\mathrm{cl,sc}}$ and the dual glued graph $\mathsf{T}^*_{\mathrm{gl}} = \mathsf{T}_{\mathrm{odd}} \cup \mathsf{T}_{\mathrm{cl,sc}^*}$.

4.3.2 Decoding subgraphs

In the following, we describe how to process the measurement results in order to determine a suitable correction operation on the post-measurement on qubits $\{q_1, q_2\}$ to obtain the Bell state Φ. More precisely, the correction is a Pauli operator of the form $Z^{\hat{c}_X} X^{\hat{c}_Z}$, where $\hat{c}_X$ and $\hat{c}_Z$ are computed from the single-qubit measurements results by a classical computation we describe below. The computation of $\hat{c}_X$ requires finding a minimal matching of a subset of marked vertices on a certain “decoding” graph $\mathsf{T}_{\mathrm{cl,dec}}$ with interior and exterior vertices. The computation of $\hat{c}_Z$ proceeds in a similar “dual” manner and involves a “dual decoding graph” $\mathsf{T}_{\mathrm{cl,dec}^*}$, see Fig. 6 for an illustration of these graphs.

The graphs $\mathsf{T}_{\mathrm{cl,dec}}$ and $\mathsf{T}_{\mathrm{cl,dec}^*}$ play a central role in our protocol: We will relate the edges of the graphs $\mathsf{T}_{\mathrm{cl,dec}}$ and $\mathsf{T}_{\mathrm{cl,dec}^*}$ with the measured qubits. We will argue how the measurement results determine certain subsets of edges in these two graphs. These subsets are used to obtain certain subsets of marked vertices (by taking boundaries). The pair $(\hat{c}_X, \hat{c}_Z)$ is computed from these subsets of marked vertices.

The decoding graph $\mathsf{T}_{\mathrm{cl,dec}} = (\mathsf{V}_{\mathrm{cl,dec}}, \mathsf{E}_{\mathrm{cl,dec}})$ is the result of gluing two copies of the subgraph $\mathsf{T}_{\mathrm{dec}} \subset \mathsf{T}_{\mathrm{sc}}$ (instead of two copies of the entire graph T_{sc}) to $\mathsf{T}_{\mathrm{even}}$, see Fig. 6a. By construction, the resulting decoding graph $\mathsf{T}_{\mathrm{cl,dec}}$ is a subgraph of T_{gl}. Concisely, we have

$$\mathsf{T}_{\mathrm{cl,dec}} = \mathsf{T}_{\mathrm{even}} \cup (\mathsf{T}_{\mathrm{dec}} \times \{1, R\}) \subset \mathsf{T}_{\mathrm{gl}} \ . \tag{40}$$

The dual decoding graph $\mathsf{T}_{\mathrm{cl,dec}^*} = (\mathsf{V}_{\mathrm{cl,dec}^*}, \mathsf{E}_{\mathrm{cl,dec}^*})$ is a subgraph of the dual glued graph $\mathsf{T}^*_{\mathrm{gl}}$ and is defined in a similar manner by

$$\mathsf{T}_{\mathrm{cl,dec}^*} = \mathsf{T}_{\mathrm{odd}} \cup (\mathsf{T}_{\mathrm{dec}^*} \times \{1, R\}) \subset \mathsf{T}^*_{\mathrm{gl}} \ , \tag{41}$$

see Fig. 6b.

We explain the relationship between the qubits that are measured and the edges of the graphs $\mathsf{T}_{\mathrm{cl,dec}}$ and $\mathsf{T}_{\mathrm{cl,dec}^*}$ using a bijection

$$\Lambda : \mathsf{E}_{\mathrm{cl,dec}} \cup \mathsf{E}_{\mathrm{cl,dec}^*} \to \mathcal{C} \setminus \{q_1, q_2\} \ .$$

called the “labeling” map. The map Λ identifies each edge of the graphs $\mathsf{T}_{\mathrm{cl,dec}}$ and $\mathsf{T}_{\mathrm{cl,dec}^*}$ with the site of a qubit located at the midpoint of the edge or at one of the vertices adjacent to the edge: Let $e = \{u, v\} \in \mathsf{E}_{\mathrm{cl,dec}} \cup \mathsf{E}_{\mathrm{cl,dec}^*}$ be one of edges of the graphs $\mathsf{T}_{\mathrm{cl,dec}}$ and $\mathsf{T}_{\mathrm{cl,dec}^*}$. If $d(u, v) = 2$, then Λ

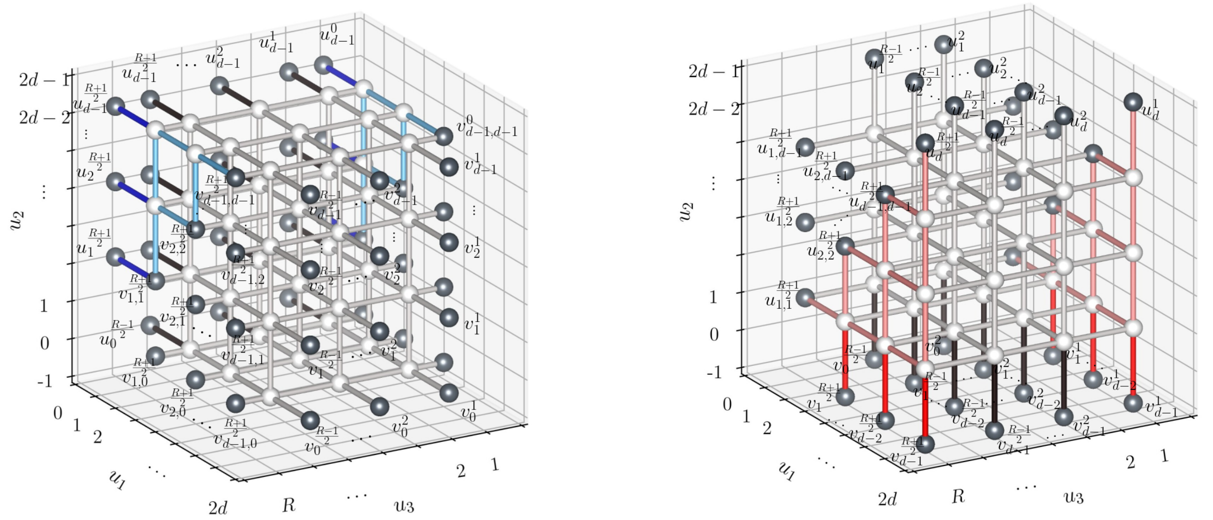

(a)

$\mathsf{T}_{\text{cl,dec}} = (\mathsf{V}_{\text{cl,dec}}, \mathsf{E}_{\text{cl,dec}})$		
subsets of $\mathsf{E}_{\text{cl,dec}}$	color	measurement
$\mathcal{L}_{\text{cl},X} \cap \mathcal{A}$	(black)	Z-basis
$(\mathsf{E}_{\text{cl,dec}} \setminus \mathcal{L}_{\text{cl},X}) \cap \mathcal{A}$	(light grey)	Z-basis
$\mathcal{L}_{\text{cl},X} \cap \mathcal{X}$	(blue)	X-basis
$(\mathsf{E}_{\text{cl,dec}} \setminus \mathcal{L}_{\text{cl},X}) \cap \mathcal{X}$	(light blue)	X-basis

(b)

$\mathsf{T}_{\text{cl,dec}^*} = (\mathsf{V}_{\text{cl,dec}}, \mathsf{E}_{\text{cl,dec}})$		
subsets of $\mathsf{E}_{\text{cl,dec}^*}$	color	measurement
$\mathcal{L}^*_{\text{cl},Z} \cap \mathcal{A}$	(black)	Z-basis
$(\mathsf{E}_{\text{cl,dec}^*} \setminus \mathcal{L}^*_{\text{cl},Z}) \cap \mathcal{A}$	(light grey)	Z-basis
$\mathcal{L}^*_{\text{cl},Z} \cap \mathcal{Z}$	(red)	Z-basis
$(\mathsf{E}_{\text{cl,dec}^*} \setminus \mathcal{L}^*_{\text{cl},Z}) \cap \mathcal{Z}$	(pink)	Z-basis

Figure 6: The decoding graph $\mathsf{T}_{\text{cl,dec}}$ as well as the dual decoding graph $\mathsf{T}_{\text{cl,dec}^*}$. External vertices $\mathsf{V}^{\text{ext}}_{\text{cl,dec}}$ and $\mathsf{V}^{\text{ext}}_{\text{cl,dec}^*}$ (black spheres) and internal vertices $\mathsf{V}^{\text{int}}_{\text{cl,dec}}$ and $\mathsf{V}^{\text{int}}_{\text{cl,dec}^*}$ (white spheres) are illustrated. The tables describe in which basis qubits corresponding to the edges are measured.

maps the edge e to the midpoint $\Lambda(e) = \frac{u+v}{2}$ of the vertices u and v. Otherwise, i.e., if $d(u,v) \neq 2$, then we must have $d(u,v) = 1$ and it follows that e belongs to $\mathsf{E}_{\text{even}} \subset \mathsf{E}_{\text{cl,dec}}$, and is of the form

$$\{u,v\} = \begin{cases} \{(u_1,u_2,1),(u_1,u_2,2)\} & \text{if } \{u_3,v_3\} = \{1,2\} \\ \{(u_1,u_2,R-1),(u_1,u_2,R)\} & \text{if } \{u_3,v_3\} = \{R-1,R\} \end{cases} \qquad \text{with} \qquad u_1,u_2 \text{ even}$$

as stated in (39) of Section 4.3.1. In this case, the edge e is mapped by Λ to the site $(u_1,u_2,1)$ if $\{u_3,v_3\} = \{1,2\}$ or the site (u_1,u_2,R) otherwise. In summary, we can write the map Λ as

$$\Lambda(\{u,v\}) = \begin{cases} (u_1,u_2,1) & \text{if } \{u,v\} \in \mathsf{E}_{\text{cl,dec}},\ d(u,v) = 1 \text{ and } \{u_3,v_3\} = \{1,2\} \\ (u_1,u_2,R) & \text{if } \{u,v\} \in \mathsf{E}_{\text{cl,dec}},\ d(u,v) = 1 \text{ and } \{u_3,v_3\} = \{R-1,R\} \\ \left(\frac{u_1+v_1}{2}, \frac{u_2+v_2}{2}, \frac{u_3+v_3}{2}\right) & \text{otherwise.} \end{cases} \tag{42}$$

It is easy to check that the map Λ is a bijection.

Using the labeling map Λ, we can identify each edge of $\mathsf{T}_{\text{cl,dec}}$ or $\mathsf{T}_{\text{cl,dec}^*}$ with one of the measured qubits, or equivalently, the site of the latter. We say an edge $e \in \mathsf{E}_{\text{cl,dec}} \cup \mathsf{E}_{\text{cl,dec}^*}$ is labeled by a site $w \in \mathcal{C} \setminus \{q_1,q_2\}$ if $\Lambda(e) = w$.

With the labeling map Λ, we have

$$\begin{aligned} \mathcal{X} &= \Lambda(\mathsf{E}_{\text{dec}} \times \{1,R\}) \\ \mathcal{Z} &= \Lambda(\mathsf{E}_{\text{dec}^*} \times \{1,R\}) \\ \Lambda(\mathsf{E}_{\text{cl,dec}}) \cap \mathcal{A} &= \Lambda(\mathsf{E}_{\text{even}}) \\ \Lambda(\mathsf{E}_{\text{cl,dec}^*}) \cap \mathcal{A} &= \Lambda(\mathsf{E}_{\text{odd}})\ . \end{aligned} \tag{43}$$

Sometimes we simply denote an edge e by the corresponding label $\Lambda(e)$, leaving the map Λ implicit.

Following this convention Eq. (43) becomes

$$\begin{aligned}
\mathcal{X} &= \mathsf{E}_{\text{dec}} \times \{1, R\} \\
\mathcal{Z} &= \mathsf{E}_{\text{dec}^*} \times \{1, R\} \\
\mathsf{E}_{\text{cl,dec}} \cap \mathcal{A} &= \mathsf{E}_{\text{even}} \\
\mathsf{E}_{\text{cl,dec}^*} \cap \mathcal{A} &= \mathsf{E}_{\text{odd}} \ .
\end{aligned}$$

Because of the definitions (40) and (41) of the graphs $\mathsf{T}_{\text{cl,dec}}$ and $\mathsf{T}_{\text{cl,dec}^*}$, this allows us to write the set of edges $\mathsf{E}_{\text{cl,dec}}$ and $\mathsf{E}_{\text{cl,dec}^*}$ as unions of pairwise disjoint subsets $\mathcal{X}$, $\mathcal{Z}$, $\mathsf{E}_{\text{cl,dec}} \cap \mathcal{A}$ and $\mathsf{E}_{\text{cl,dec}^*} \cap \mathcal{A}$ of the measured qubits $\mathcal{C} \setminus \{q_1, q_2\}$, namely

$$\begin{aligned}
\mathsf{E}_{\text{cl,dec}} &= \mathcal{X} \cup (\mathsf{E}_{\text{cl,dec}} \cap \mathcal{A}) \\
\mathsf{E}_{\text{cl,dec}^*} &= \mathcal{Z} \cup (\mathsf{E}_{\text{cl,dec}^*} \cap \mathcal{A})
\end{aligned} \tag{44}$$

In our entanglement generation protocol, we will use (44) to interpret the measurement outcomes $(a, x, z) \in \{0, 1\}^{\mathcal{A} \cup \mathcal{X} \cup \mathcal{Z}}$ as defining two subsets of $\mathsf{E}_{\text{cl,dec}}$ and $\mathsf{E}_{\text{cl,dec}^*}$, respectively.

4.3.3 Recovery sets and internal vertices of $\mathsf{T}_{\text{cl,dec}}$ and $\mathsf{T}_{\text{cl,dec}^*}$

We define recovery sets $\mathcal{L}_{\text{cl},X} \subset \mathsf{E}_{\text{cl,dec}}$ and $\mathcal{L}^*_{\text{cl},Z} \subset \mathsf{E}_{\text{cl,dec}^*}$ to be used later, see Fig. 6 for an illustration. The role of these sets in the context of the graph state will be clarified below, see Lemma 4.2.

The recovery set $\mathcal{L}_{\text{cl},X} \subset \mathsf{E}_{\text{cl,dec}}$ consists of edges incident on degree-1 vertices (so-called dangling edges in the terminology of [1]) on the left rough boundary of $\mathsf{T}_{\text{cl,dec}}$. That is,

$$\begin{aligned}
\mathcal{L}_{\text{cl},X} = \{\{(0, u_2, u_3), (2, u_2, u_3)\} \mid u_2 \geq 2, u_2 \text{ even}, u_3 \in \{1, R\}\} \\
\cup \{\{(0, u_2, u_3), (2, u_2, u_3)\} \mid u_2, u_3 \text{ even } \} \ ,
\end{aligned}$$

or more succinctly (labeling edges by sites with the map Λ)

$$\mathcal{L}_{\text{cl},X} = \{(1, u_2, u_3) \mid u_2 \geq 2, u_2 \text{ even}, u_3 \in \{1, R\}\} \cup \{(1, u_2, u_3) \mid u_2 \geq 2, u_2, u_3 \text{ even } \} \ . \tag{45}$$

Observe that the first set in this union is simply $\mathcal{L}_X \times \{1, R\}$, i.e., it consists of two copies of the set $\mathcal{L}_X$ introduced for the surface code in Section 1.4. We can also write this set as $\mathcal{L}_{\text{cl},X} \cap \mathcal{B}$ or equivalently as $\mathcal{L}_{\text{cl},X} \cap \mathcal{X}$ (by the fact that $\mathcal{B} \cap \mathsf{E}_{\text{cl,dec}} = \mathcal{X}$) using the labels. Similarly, the second set in the union (45) is $\mathcal{L}_{\text{cl},X} \cap \mathcal{A}$. In summary, the recovery set $\mathcal{L}_{\text{cl},X}$ is the disjoint union

$$\mathcal{L}_{\text{cl},X} = (\mathcal{L}_{\text{cl},X} \cap \mathcal{A}) \cup (\mathcal{L}_{\text{cl},X} \cap \mathcal{B}) \ .$$

The dual recovery set $\mathcal{L}^*_{\text{cl},Z} \subset \mathsf{E}_{\text{cl,dec}^*}$ consists of edges incident on degree-1 vertices at the bottom of $\mathsf{E}_{\text{cl,dec}^*}$. For every site $(u_1, u_2, u_3) \in \{(o, -1, o)\} \setminus \{q_1, q_2\} \in \widetilde{\mathcal{C}}^*$, there exists such an edge incident on the vertex at $(u_1, 1, u_3)$; these edges are labeled by their midpoint $(u_1, 0, u_3)$. Such an edge belongs to $\mathcal{A}$ if and only if $u_3 \notin \{1, R\}$, and to $\mathcal{B}$ otherwise. In summary, the recovery set $\mathcal{L}^*_{\text{cl},Z}$ is the disjoint union

$$\mathcal{L}^*_{\text{cl},Z} = (\mathcal{L}^*_{\text{cl},Z} \cap \mathcal{A}) \cup (\mathcal{L}^*_{\text{cl},Z} \cap \mathcal{B})$$

where

$$\begin{aligned}
\mathcal{L}^*_{\text{cl},Z} \cap \mathcal{A} &= \{(u_1, u_2, u_3) \in \{(o, 0, o)\} \mid u_1 \notin \{1, R\}\} \\
\mathcal{L}^*_{\text{cl},Z} \cap \mathcal{B} &= \{(u_1, u_2, u_3) \in \{(o, 0, o)\} \mid u_1 \in \{1, R\}\} \setminus \{q_1, q_2\} \ .
\end{aligned}$$

Finally, let us define the set $\mathsf{V}^{\text{int}}_{\text{cl,dec}} \subset \mathsf{V}_{\text{cl,dec}}$ of internal vertices of the decoding graph $\mathsf{T}_{\text{cl,dec}}$ and similarly, the set $\mathsf{V}^{\text{int}}_{\text{cl,dec}^*} \subset \mathsf{V}_{\text{cl,dec}^*}$ of internal vertices of the dual decoding graph $\mathsf{T}_{\text{cl,dec}^*}$. We call their complements $\mathsf{V}^{\text{ext}}_{\text{cl,dec}} := \mathsf{V}_{\text{cl,dec}} \setminus \mathsf{V}^{\text{int}}_{\text{cl,dec}}$ and $\mathsf{V}^{\text{ext}}_{\text{cl,dec}^*} := \mathsf{V}_{\text{cl,dec}^*} \setminus \mathsf{V}^{\text{int}}_{\text{cl,dec}^*}$ the sets of external vertices, see Fig. 6.

For the decoding graph $\mathsf{T}_{\text{cl,dec}}$, the set of internal vertices is

$$\mathsf{V}^{\text{int}}_{\text{cl,dec}} = \{u \in \mathsf{V}_{\text{even}} \mid \text{neigh}(u) \subset \mathsf{E}_{\text{even}}\} \cup \left(\mathsf{V}^{\text{int}}_{\text{dec}} \times \{1, R\}\right) \ . \tag{46}$$

The set of internal vertices of the graph $\mathsf{T}_{\text{cl,dec}^*}$ is defined similarly as

$$\mathsf{V}^{\text{int}}_{\text{cl,dec}^*} = \{u \in \mathsf{V}_{\text{odd}} \mid \text{neigh}(u) \subset \mathsf{E}_{\text{odd}}\} \cup \left(\mathsf{V}^{\text{int}}_{\text{dec}^*} \times \{1, R\}\right) \ ,$$

or equivalently as

$$\mathsf{V}^{\text{int}}_{\text{cl,dec}^*} = \{u \in \mathsf{V}_{\text{cl,dec}^*} \mid \text{neigh}(u) \subset \mathsf{E}_{\text{cl,dec}^*}\} \ .$$

4.4 Stabilizers of the cluster state

In this section, we consider certain products of the stabilizer generators $\{G_u\}_{u\in\mathcal{C}}$ of the cluster state $W\left|0^{\mathcal{C}}\right\rangle$ defined by (38). The first set of operators will be used to define an error syndrome computable from single-qubit measurements performed in our protocol. To introduce these operators, we denote by $\text{Inci}_G(v)$ the set of all edges of a graph G which are incident on a vertex v. Here the graph G is either $\mathsf{T}_{\text{cl,dec}}$ or $\mathsf{T}_{\text{cl,dec}^*}$. Again using the labeling map Λ (cf. (42)), we will sometimes regard $\text{Inci}_G(v)$ as a set of sites (associated with the measured qubits). Key to our procedure is the fact that certain products of the stabilizer generators (38) of the cluster state $W\left|0^{\mathcal{C}}\right\rangle$ have supports given by sets of the form $\text{Inci}_{\mathsf{T}_{\text{cl,dec}}}(v)$ for $v \in \mathsf{V}_{\text{cl,dec}}$ or $\text{Inci}_{\mathsf{T}_{\text{cl,dec}^*}}(v)$ for $v \in \mathsf{V}_{\text{cl,dec}^*}$. More precisely, we have the following:

Lemma 4.1. *Define S^u for $u \in \mathsf{V}^{\text{int}}_{\text{cl,dec}}$ as*

$$S^u := \begin{cases} \prod_{v\in\text{neigh}(u)} G_v & \text{if } u_3 \notin \{1, R\} \\ G_u & \text{if } u_3 \in \{1, R\} \ . \end{cases} \tag{47}$$

Then

$$S^u = \left(\prod_{v\in\text{Inci}_{\mathsf{T}_{\text{cl,dec}}}(u)\cap\mathcal{A}} Z_v\right)\left(\prod_{v\in\text{Inci}_{\mathsf{T}_{\text{cl,dec}}}(u)\cap\mathcal{B}} X_v\right) \ . \tag{48}$$

Similarly, define S^u for $u \in \mathsf{V}^{\text{int}}_{\text{cl,dec}^*}$ as

$$S^u = \begin{cases} \prod_{v\in\text{neigh}(u)} G_v & \text{if } u_3 \notin \{1, R\} \\ G_{u\pm(0,0,1)} \prod_{v\in\text{neigh}(u)\cap\mathcal{B}} G_v & \text{if } u_3 \in \{1, R\} \end{cases} \tag{49}$$

where the sign $\pm$ in (49) is "$+$" if u is situated in the front layer, and "$-$" if u is situated in the back layer so that $u \pm (0, 0, 1) \in \mathcal{C}$. Then

$$S^u = \prod_{v\in\text{Inci}_{\mathsf{T}_{\text{cl,dec}^*}}(u)} Z_v \qquad \text{for all} \qquad u \in \mathsf{V}^{\text{int}}_{\text{cl,dec}^*} \ . \tag{50}$$

We include a proof of this lemma in Appendix A.

We also need two additional products S^X and S^Z of the stabilizer generators $\{G_u\}_{u\in\mathcal{C}}$. These have support on the two qubits $\{q_1, q_2\}$ between which the protocol establishes entanglement, and their eigenvalues fix the state of these two qubits as described below (see Lemma 4.3).

Lemma 4.2. *Define the operators*

$$S^X := \prod_{u\in\mathcal{L}_{\text{cl},X}\cap\mathcal{B}} G_u \qquad \text{and} \qquad S^Z := \prod_{u\in\mathcal{L}^*_{\text{cl},Z}\cup\{q_1,q_2\}} G_u \ .$$

Then

$$S^X = X_{q_1} X_{q_2} X\left(\mathcal{L}_{\text{cl},X} \cap \mathcal{B}\right) Z\left(\mathcal{L}_{\text{cl},X} \cap \mathcal{A}\right) \tag{51}$$

$$S^Z = Z_{q_1} Z_{q_2} Z\left(\mathcal{L}^*_{\text{cl},Z} \cap \mathcal{B}\right) Z\left(\mathcal{L}^*_{\text{cl},Z} \cap \mathcal{A}\right) \ . \tag{52}$$

We note that similar expressions for products of stabilizer generators have been exploited in the seminal work [11]. For completeness, we give a proof of this lemma in Appendix A.

4.5 Description of the entanglement generation protocol

The entanglement generation protocol is given as Algorithm 2 below. It takes as input a state $|\Psi_{\text{in}}\rangle$ of the qubits on $\mathcal{C}$ and produces a two-qubit state $|\Psi_{\text{out}}\rangle$ on qubits $\{q_1, q_2\}$.

In the following, we again identify a string $a \in \{0,1\}^{\mathcal{C}}$ with a subset of $\mathcal{C}$ (interpreting a as its characteristic function). The bit-wise XOR $a \oplus b$ of two strings $a, b \in \{0,1\}^{\mathcal{C}}$ then corresponds to taking the symmetric difference of the associated sets.

Algorithm 2 Fault-tolerant entanglement generation protocol

Require: Input: A state on $(\mathbb{C}^2)^{\otimes \mathcal{C}}$, where $\mathcal{C} = \mathcal{C}[d \times d \times R]$ is the set of the locations of qubits.

1: Perform the following measurements: measure

every qubit in the set	in the	denote the outcome by
$\mathcal{X}$	X-basis	$x \in \{0,1\}^{\mathcal{X}}$
$\mathcal{Z}$	Z-basis	$z \in \{0,1\}^{\mathcal{Z}}$
$\mathcal{A}$	Z-basis	$a \in \{0,1\}^{\mathcal{A}}$

2: Compute the boundaries of the edges $(a \cap \mathsf{E}_{\text{cl,dec}}) \oplus x$ and $(a \cap \mathsf{E}_{\text{cl,dec}^*}) \oplus z$, respectively, i.e., set

$$s \leftarrow \partial_{\mathsf{T}_{\text{cl,dec}}}((a \cap \mathsf{E}_{\text{cl,dec}}) \oplus x)$$
$$s^* \leftarrow \partial_{\mathsf{T}_{\text{cl,dec}^*}}((a \cap \mathsf{E}_{\text{cl,dec}^*}) \oplus z)$$

3: Compute minimal matchings

$$m \leftarrow \mathsf{MinMatch}_{\mathsf{T}_{\text{cl,dec}}}(s)$$
$$m^* \leftarrow \mathsf{MinMatch}_{\mathsf{T}_{\text{cl,dec}^*}}(s^*)$$

4: Compute the bits

$$\hat{s}^X \leftarrow \langle\!\langle m, \mathcal{L}_{\text{cl},X} \rangle\!\rangle$$
$$\hat{s}^Z \leftarrow \left\langle\!\!\left\langle m^*, \mathcal{L}^*_{\text{cl},Z} \right\rangle\!\!\right\rangle$$

5: Determine the bits

$$\hat{c}_X \leftarrow \langle\!\langle a, \mathcal{L}_{\text{cl},X} \cap \mathcal{A} \rangle\!\rangle \oplus \langle\!\langle x, \mathcal{L}_{\text{cl},X} \cap \mathcal{B} \rangle\!\rangle \oplus \hat{s}^X$$
$$\hat{c}_Z \leftarrow \left\langle\!\!\left\langle a, \mathcal{L}^*_{\text{cl},Z} \cap \mathcal{A} \right\rangle\!\!\right\rangle \oplus \left\langle\!\!\left\langle z, \mathcal{L}^*_{\text{cl},Z} \cap \mathcal{B} \right\rangle\!\!\right\rangle \oplus \hat{s}^Z$$

6: Apply $Z^{\hat{c}_X} X^{\hat{c}_Z}$ to q_1.

7: Return the two-qubit state on the qubits $\{q_1, q_2\}$.

We will show that if the input state $|\Psi_{\text{in}}\rangle$ is the cluster state $W\left|0^{\mathcal{C}}\right\rangle$, then the output of Algorithm 2 is the two-qubit Bell state $|\Phi\rangle$. More generally, we will show that the procedure yields a Bell state $\left|\Phi_{(\alpha,\beta)}\right\rangle$ if we start with a cluster state corrupted by a Pauli error.

4.6 Entanglement generation from a corrupted cluster state

In this section, we examine the result of running Algorithm 2 on the corrupted cluster state

$$\left|\Psi^{\mathcal{C}}_{\text{in}}\right\rangle = EW\left|0^{\mathcal{C}}\right\rangle \tag{53}$$

for a fixed Pauli error E. We show that the final state on qubits q_1, q_2 is one of the four Bell states $\{|\Phi_{(\alpha,\beta)}\rangle\}_{\alpha,\beta\in\{0,1\}}$, and give a formula for $(\alpha,\beta)\in\{0,1\}^2$ in terms of the error E (see Theorem 4.6). This is the basis of our analysis of local stochastic noise in Section 5.

To determine the state at the end of the protocol, first consider the post-measurement state

$$|\psi_{\mathrm{pm}}(a,x,z,E)\rangle = \frac{1}{\sqrt{p(a,x,z|E)}}\left(\langle a|_{\mathcal{A}} \otimes \langle x|_{\mathcal{X}} H(\mathcal{X})^\dagger \otimes \langle z|_{\mathcal{Z}} \otimes I_{\{q_1,q_2\}}\right) EW\left|0^{\mathcal{C}}\right\rangle \tag{54}$$

obtained after step 1 of the algorithm on qubits $\{q_1,q_2\}$. Here $p(a,x,z \mid E)$ denotes the probability of obtaining the outcomes (a,x,z) given a Pauli error E, i.e., for the input state (53). This state is a Bell state determined by the measurement outcomes (a,x,z) and the error E as follows:

Lemma 4.3. *Let S^X and S^Z be the operators introduced in Lemma 4.2. Let*

$$\begin{aligned} c_X &:= \langle\!\langle a, \mathcal{L}_{\mathrm{cl},X} \cap \mathcal{A}\rangle\!\rangle \oplus \langle\!\langle x, \mathcal{L}_{\mathrm{cl},X} \cap \mathcal{B}\rangle\!\rangle \oplus \mathsf{Syn}(S^X, E) \\ c_Z &:= \langle\!\langle a, \mathcal{L}^*_{\mathrm{cl},Z} \cap \mathcal{A}\rangle\!\rangle \oplus \langle\!\langle z, \mathcal{L}^*_{\mathrm{cl},Z} \cap \mathcal{B}\rangle\!\rangle \oplus \mathsf{Syn}(S^Z, E)\ . \end{aligned}$$

Then, given measurement outcomes (a,x,z), the post-measurement state after Step 1 of Algorithm 2 running on a corrupted input state $EW\left|0^{\mathcal{C}}\right\rangle$ is

$$|\psi_{\mathrm{pm}}(a,x,z,E)\rangle = \left|\Phi_{(c_X,c_Z)}\right\rangle\ .$$

Proof. The claim is an immediate consequence of the following two facts:

(i) The operators S^X and S^Z are defined as products of the stabilizer generators $\{G_u\}_{u\in\mathcal{C}}$ and thus stabilize the ideal cluster state $W\left|0^{\mathcal{C}}\right\rangle$.

(ii) The operators S^X and S^Z are – up to additional Pauli operators on the measured qubits – proportional to the stabilizer generators $\{X_{q_1}X_{q_2}, Z_{q_1}Z_{q_2}\}$ of the Bell state $\left|\Phi_{(0,0)}\right\rangle$, see (51) and (52) of Lemma 4.2.

Replacing $W\left|0^{\mathcal{C}}\right\rangle$ in (54) by $S^XW\left|0^{\mathcal{C}}\right\rangle$ using property (i), we observe that the post-measurement state $\psi_{\mathrm{pm}} = \psi_{\mathrm{pm}}(a,x,z,E)$ satisfies

$$\begin{aligned} \sqrt{p}\,|\psi_{\mathrm{pm}}\rangle &= \left(\langle a|_{\mathcal{A}} \otimes \langle x|_{\mathcal{X}} H(\mathcal{X})^\dagger \otimes \langle z|_{\mathcal{Z}} \otimes I_{\{q_1,q_2\}}\right) ES^XE\left|0^{\mathcal{C}}\right\rangle \\ &= (-1)^{\mathsf{Syn}(S^X,E)}\left(\langle a|_{\mathcal{A}} \otimes \langle x|_{\mathcal{X}} H(\mathcal{X})^\dagger \otimes \langle z|_{\mathcal{Z}} \otimes I_{\{q_1,q_2\}}\right) S^XE\left|0^{\mathcal{C}}\right\rangle \\ &= (-1)^{\langle\!\langle a,\mathcal{L}_{\mathrm{cl},X}\cap\mathcal{A}\rangle\!\rangle\oplus\langle\!\langle x,\mathcal{L}_{\mathrm{cl},X}\cap\mathcal{B}\rangle\!\rangle\oplus\mathsf{Syn}(S^X,E)}\left(\langle a|_{\mathcal{A}} \otimes \langle x|_{\mathcal{X}} H(\mathcal{X})^\dagger \otimes \langle z|_{\mathcal{Z}} \otimes X_{q_1}X_{q_2}\right) EW\left|0^{\mathcal{C}}\right\rangle\ , \end{aligned}$$

where $p := p(a,x,z|E)$ and where we used (51) as well as the identities

$$\begin{aligned} X(\mathcal{L}_{\mathrm{cl},X}\cap\mathcal{B})H(\mathcal{X})\,|x\rangle_{\mathcal{X}} &= (-1)^{\langle\!\langle x,\mathcal{L}_{\mathrm{cl},X}\cap\mathcal{B}\rangle\!\rangle}H(\mathcal{X})\,|x\rangle_{\mathcal{X}} \\ Z(\mathcal{L}_{\mathrm{cl},X}\cap\mathcal{A})\,|a\rangle_{\mathcal{A}} &= (-1)^{\langle\!\langle a,\mathcal{L}_{\mathrm{cl},X}\cap\mathcal{A}\rangle\!\rangle}\,|a\rangle_{\mathcal{A}}\ . \end{aligned}$$

We conclude that

$$\begin{aligned} |\psi_{\mathrm{pm}}\rangle &= (-1)^{\langle\!\langle a,\mathcal{L}_{\mathrm{cl},X}\cap\mathcal{A}\rangle\!\rangle\oplus\langle\!\langle x,\mathcal{L}_{\mathrm{cl},X}\cap\mathcal{B}\rangle\!\rangle\oplus\mathsf{Syn}(S^X,E)}X_{q_1}X_{q_2}\,|\psi_{\mathrm{pm}}\rangle \\ &= (-1)^{c_X}X_{q_1}X_{q_2}\,|\psi_{\mathrm{pm}}\rangle\ , \end{aligned}$$

by definition of c_X.

In an analogous manner, we can check $|\psi_{\mathrm{pm}}\rangle = (-1)^{c_Z}Z_{q_1}Z_{q_2}\,|\psi_{\mathrm{pm}}\rangle$ by replacing $W\left|0^{\mathcal{C}}\right\rangle$ in (54) by $S^ZW\left|0^{\mathcal{C}}\right\rangle$. Thus ψ_{pm} is stabilized by $\{(-1)^{c_X}X_{q_1}X_{q_2}, (-1)^{c_Z}Z_{q_1}Z_{q_2}\}$. This implies the claim. □

According to Lemma 4.3, the post-measurement state is fully determined by the single-qubit measurement results (a,x,z) and the bits $\mathsf{Syn}(S^X,E)$ and $\mathsf{Syn}(S^Z,E)$. These bits in turn are determined by the physical errors on qubits $\{q_1,q_2\}$ as well as a certain restriction of the error as specified in the following statement:

Lemma 4.4. *Let E be a Pauli operator on $\mathcal{C}$ and let S^X and S^Z be the Pauli operators defined in Lemma 4.2. Let us decompose E into a product $E = E^X E^Z$ of Pauli-X- and Pauli-Z-operators, respectively. Define*

$$E_{\mathrm{gl}} = E^X|_{\mathsf{E}_{\mathrm{cl,dec}} \cap \mathcal{A}} E^Z|_{\mathcal{X}} \qquad \textit{and} \tag{55}$$

$$E^*_{\mathrm{gl}} = E^X|_{\mathsf{E}_{\mathrm{cl,dec}^*}} \tag{56}$$

where $F|_\Omega := \prod_{j\in\Omega} F_j$ for any Pauli operator $F = \prod_{j\in\mathcal{C}} F_j$ on $\mathcal{C}$ and $\Omega \subset \mathcal{C}$. Then

$$\mathsf{Syn}(S^X, E) = \langle\!\langle \{q_1, q_2\}, \mathrm{supp}(E^Z)\rangle\!\rangle \oplus \langle\!\langle \mathcal{L}_{\mathrm{cl},X}, \mathrm{supp}(E_{\mathrm{gl}})\rangle\!\rangle \tag{57}$$

$$\mathsf{Syn}(S^Z, E) = \langle\!\langle \{q_1, q_2\}, \mathrm{supp}(E^X)\rangle\!\rangle \oplus \langle\!\langle \mathcal{L}^*_{\mathrm{cl},Z}, \mathrm{supp}(E^*_{\mathrm{gl}})\rangle\!\rangle \ . \tag{58}$$

Proof. Let E be an arbitrary Pauli operator on $\mathcal{C}$. Using expression (51) for S^X, we have

$$\begin{aligned}
\mathsf{Syn}(S^X, E) &= \mathsf{Syn}\left(X_{q_1} X_{q_2} X\left(\mathcal{L}_{\mathrm{cl},X} \cap \mathcal{B}\right) Z\left(\mathcal{L}_{\mathrm{cl},X} \cap \mathcal{A}\right), E\right)\\
&= \langle\!\langle \{q_1, q_2\}, \mathrm{supp}(E^Z)\rangle\!\rangle \oplus \langle\!\langle \mathcal{L}_{\mathrm{cl},X} \cap \mathcal{B}, \mathrm{supp}(E^Z)\rangle\!\rangle \oplus \langle\!\langle \mathcal{L}_{\mathrm{cl},X} \cap \mathcal{A}, \mathrm{supp}(E^X)\rangle\!\rangle\\
&= \langle\!\langle \{q_1, q_2\}, \mathrm{supp}(E^Z)\rangle\!\rangle \oplus \langle\!\langle \mathcal{L}_{\mathrm{cl},X} \cap \mathcal{B}, \mathrm{supp}(E^Z|_{\mathcal{X}})\rangle\!\rangle \oplus \langle\!\langle \mathcal{L}_{\mathrm{cl},X} \cap \mathcal{A}, \mathrm{supp}(E^X|_{\mathsf{E}_{\mathrm{cl,dec}} \cap \mathcal{A}})\rangle\!\rangle\\
&= \langle\!\langle \{q_1, q_2\}, \mathrm{supp}(E^Z)\rangle\!\rangle \oplus \langle\!\langle \mathcal{L}_{\mathrm{cl},X}, \mathrm{supp}(E_{\mathrm{gl}})\rangle\!\rangle
\end{aligned}$$

by definition of E_{gl}, see Eq. (55). A similar calculation yields

$$\begin{aligned}
\mathsf{Syn}(S^Z, E) &= \langle\!\langle \{q_1, q_2\}, \mathrm{supp}(E^X)\rangle\!\rangle \oplus \langle\!\langle \mathcal{L}^*_{\mathrm{cl},Z} \cap \mathcal{B}, \mathrm{supp}(E^X|_{\mathcal{Z}})\rangle\!\rangle \oplus \langle\!\langle \mathcal{L}^*_{\mathrm{cl},Z} \cap \mathcal{A}, \mathrm{supp}\left(E^X|_{\mathcal{A}_{\mathrm{dec}^*}}\right)\rangle\!\rangle\\
&= \langle\!\langle \{q_1, q_2\}, \mathrm{supp}(E^X)\rangle\!\rangle \oplus \langle\!\langle \mathcal{L}^*_{\mathrm{cl},Z}, \mathrm{supp}(E^*_{\mathrm{gl}})\rangle\!\rangle \ ,
\end{aligned}$$

as claimed. □

Unfortunately, the operators E_{gl} and E^*_{gl} defined in (55) and (56) are not determined by the measurement results (a, x, z). In particular, $\mathsf{Syn}(S^X, E)$ and $\mathsf{Syn}(S^Z, E)$ cannot be computed from the measurement outcomes. Algorithm 2 therefore generates estimates $\hat{s}^X$ and $\hat{s}^Z$ for these bits, see step (4).

To motivate the definition of these bits, and compute the final state after the entanglement generation protocol, we discuss how the measurement results (a, x, z) constrain the error E, or more precisely the operators E_{gl} and E^*_{gl}. Observe that by their definitions (55) and (56), these operators are fully determined by the supports $\mathrm{supp}(E_{\mathrm{gl}})$ and $\mathrm{supp}(E^*_{\mathrm{gl}})$. We obtain constraints on these sets expressed in terms of the strings s, s^* computed in step 2 of Algorithm 2.

Lemma 4.5. *The subsets $s \subset \mathsf{V}^{\mathrm{int}}_{\mathrm{cl,dec}}$ and $s^* \subset \mathsf{V}^{\mathrm{int}}_{\mathrm{cl,dec}^*}$ computed (from the measurement outcomes (a, x, z)) in Step 2 satisfy*

$$s = \partial_{\mathsf{T}_{\mathrm{cl,dec}}}(\mathrm{supp}(E_{\mathrm{gl}})) \tag{59}$$

$$s^* = \partial_{\mathsf{T}_{\mathrm{cl,dec}^*}}(\mathrm{supp}(E^*_{\mathrm{gl}})) \ . \tag{60}$$

Proof. Let $s = (s_u) \in \{0,1\}^{\mathsf{V}^{\mathrm{int}}_{\mathrm{cl,dec}}}$ be the string computed in step 2, i.e.,

$$s = \partial_{\mathsf{T}_{\mathrm{cl,dec}}}\left(a \cap \mathsf{E}_{\mathrm{cl,dec}} \oplus x\right) \ ,$$

and let $u \in \mathsf{V}^{\mathrm{int}}_{\mathrm{cl,dec}}$. Linearity of the map $\partial_{\mathsf{T}_{\mathrm{cl,dec}}}$ implies that

$$\begin{aligned}
s_u &= \left(\partial_{\mathsf{T}_{\mathrm{cl,dec}}}(a)\right)_u \oplus \left(\partial_{\mathsf{T}_{\mathrm{cl,dec}}}(x)\right)_u\\
&= \langle\!\langle a, \mathrm{Inci}_{\mathsf{T}_{\mathrm{cl,dec}}}(u)\rangle\!\rangle \oplus \langle\!\langle x, \mathrm{Inci}_{\mathsf{T}_{\mathrm{cl,dec}}}(u)\rangle\!\rangle \ .
\end{aligned} \tag{61}$$

Since $\left(\partial_{\mathsf{T}_{\mathrm{cl,dec}}}(\mathrm{supp}(E_{\mathrm{gl}}))\right)_u = \langle\!\langle \mathrm{Inci}_{\mathsf{T}_{\mathrm{cl,dec}}}(u), \mathrm{supp}(E_{\mathrm{gl}})\rangle\!\rangle$, we need to show that

$$\langle\!\langle a, \mathrm{Inci}_{\mathsf{T}_{\mathrm{cl,dec}}}(u)\rangle\!\rangle \oplus \langle\!\langle x, \mathrm{Inci}_{\mathsf{T}_{\mathrm{cl,dec}}}(u)\rangle\!\rangle = \langle\!\langle \mathrm{Inci}_{\mathsf{T}_{\mathrm{cl,dec}}}(u), \mathrm{supp}(E_{\mathrm{gl}})\rangle\!\rangle \ . \tag{62}$$

We first show that

$$\langle\!\langle a, \mathrm{Inci}_{\mathsf{T}_{\mathrm{cl,dec}}}(u)\rangle\!\rangle \oplus \langle\!\langle x, \mathrm{Inci}_{\mathsf{T}_{\mathrm{cl,dec}}}(u)\rangle\!\rangle = \mathsf{Syn}(S^u, E)\ . \tag{63}$$

Note that S^u stabilizes the state $W\left|0^{\mathcal{C}}\right\rangle$ since it is a product of the stabilizer generators $\{G_v\}_v$ of $W\left|0^{\mathcal{C}}\right\rangle$. Replacing $W\left|0^{\mathcal{C}}\right\rangle$ in (54) by $S^uW\left|0^{\mathcal{C}}\right\rangle$ yields

$$\begin{aligned}
|\psi_{\mathrm{pm}}\rangle &= \frac{1}{\sqrt{p}}\left(\langle a|_{\mathcal{A}} \otimes \langle x|_{\mathcal{X}}\, H(\mathcal{X})^\dagger \otimes \langle z|_{\mathcal{Z}} \otimes I_{\{q_1,q_2\}}\right) ES^uW\left|0^{\mathcal{C}}\right\rangle \\
&= \frac{1}{\sqrt{p}}(-1)^{\mathsf{Syn}(S^u,E)}\left(\langle a|_{\mathcal{A}} \otimes \langle x|_{\mathcal{X}}\, H(\mathcal{X})^\dagger \otimes \langle z|_{\mathcal{Z}} \otimes I_{\{q_1,q_2\}}\right) S^uEW\left|0^{\mathcal{C}}\right\rangle \\
&= (-1)^{\mathsf{Syn}(S^u,E)\oplus\langle\!\langle a,\mathrm{Inci}_{\mathsf{T}_{\mathrm{cl,dec}}}(u)\rangle\!\rangle\oplus\langle\!\langle x,\mathrm{Inci}_{\mathsf{T}_{\mathrm{cl,dec}}}(u)\rangle\!\rangle}\,|\psi_{\mathrm{pm}}\rangle\ .
\end{aligned}\tag{64}$$

Here the third equality follows from the identity

$$S^u\left(|a\rangle_{\mathcal{A}}\left(H(\mathcal{X})\,|x\rangle\right)\right) = (-1)^{\langle\!\langle a,\mathrm{Inci}_{\mathsf{T}_{\mathrm{cl,dec}}}(u)\rangle\!\rangle\oplus\langle\!\langle x,\mathrm{Inci}_{\mathsf{T}_{\mathrm{cl,dec}}}(u)\rangle\!\rangle}\left(|a\rangle_{\mathcal{A}}\left(H(\mathcal{X})\,|x\rangle\right)\right)$$

which is a consequence of (48) and the fact that $a \subset \mathcal{A}$ and $x \subset \mathcal{X} \subset \mathcal{B}$ are disjoint. Identity (64) together with (61) establishes (63).

Next, we show that

$$\langle\!\langle \mathrm{Inci}_{\mathsf{T}_{\mathrm{cl,dec}}}(u), \mathrm{supp}(E_{\mathrm{gl}})\rangle\!\rangle = \mathsf{Syn}(S^u, E)\ . \tag{65}$$

Observe that according to (48), we have

$$\begin{aligned}
\mathsf{Syn}(S^u, E) &= \mathsf{Syn}\left(\left(\prod_{v\in \mathrm{Inci}_{\mathsf{T}_{\mathrm{cl,dec}}}(u)\cap\mathcal{A}} Z_v\right)\left(\prod_{v\in \mathrm{Inci}_{\mathsf{T}_{\mathrm{cl,dec}}}(u)\cap\mathcal{B}} X_v\right), E\right) \\
&= \langle\!\langle \mathrm{Inci}_{\mathsf{T}_{\mathrm{cl,dec}}}(u)\cap\mathcal{A}, \mathrm{supp}(E^X)\rangle\!\rangle \oplus \langle\!\langle \mathrm{Inci}_{\mathsf{T}_{\mathrm{cl,dec}}}(u)\cap\mathcal{B}, \mathrm{supp}(E^Z)\rangle\!\rangle
\end{aligned}$$

for any $u \in \mathsf{V}^{\mathrm{int}}_{\mathrm{cl,dec}}$. From this expression, the definition (55) of E_{gl} and the fact that $\mathcal{A}$ and $\mathcal{B}$ are disjoint, the identity (65) follows. Together (63), the claim (62) follows.

The claim for s^* can be proved in an analogous manner using (50). □

We now argue that the strings m and m^* computed in Step 3 of Algorithm 2 can be considered to be proxys for the sets $\mathrm{supp}(E_{\mathrm{gl}})$ and $\mathrm{supp}(E^*_{\mathrm{gl}})$. More precisely, we may define a Pauli error

$$\widehat{E} := \widehat{E}_{\mathrm{gl}}\widehat{E}^*_{\mathrm{gl}} \qquad \text{with} \qquad \begin{array}{rcl} \widehat{E}_{\mathrm{gl}} &:=& X(m\cap\mathcal{A})Z(m\cap\mathcal{B}) \\ \widehat{E}^*_{\mathrm{gl}} &:=& Z(m^*)\ . \end{array}$$

Then we have $m = \mathrm{supp}(\widehat{E}_{\mathrm{gl}})$ and $m^* = \mathrm{supp}(\widehat{E}^*_{\mathrm{gl}})$ by definition of these operators, and it follows immediately from the definition of m and m^* that $(\widehat{E}_{\mathrm{gl}}, \widehat{E}^*_{\mathrm{gl}})$ satisfy constraints analogous to the constraints (59), (60) obeyed by $(E_{\mathrm{gl}}, E^*_{\mathrm{gl}})$, i.e.,

$$\begin{aligned}
s &= \partial_{\mathsf{T}_{\mathrm{cl,dec}}}(\mathrm{supp}(\widehat{E}_{\mathrm{gl}})) \\
s^* &= \partial_{\mathsf{T}_{\mathrm{cl,dec}^*}}(\mathrm{supp}(\widehat{E}^*_{\mathrm{gl}}))\ .
\end{aligned}$$

Thus $\widehat{E} = \widehat{E}_{\mathrm{gl}}\widehat{E}^*_{\mathrm{gl}}$ is an error consistent with the observed syndrome (s, s^*) with the property that $\widehat{E}_{\mathrm{gl}}$ and $\widehat{E}^*_{\mathrm{gl}}$ each are of minimum weight relative to $\mathsf{V}^{\mathrm{int}}_{\mathrm{cl,dec}}$ and $\mathsf{V}^{\mathrm{int}}_{\mathrm{cl,dec}^*}$, respectively.

Because the error $\widehat{E}$ can be computed from the measurement outcomes, this motivates using the pair $\left(\mathsf{Syn}(S^X, \widehat{E}), \mathsf{Syn}(S^Z, \widehat{E})\right)$ as an estimate for $\left(\mathsf{Syn}(S^X, E), \mathsf{Syn}(S^Z, E)\right)$. Indeed, this is the reasoning underlying the definition

$$\begin{array}{rcl} \hat{s}^X &:=& \langle\!\langle \mathcal{L}_{\mathrm{cl},X}, m\rangle\!\rangle \\ \hat{s}^Z &:=& \langle\!\langle \mathcal{L}^*_{\mathrm{cl},Z}, m^*\rangle\!\rangle \end{array} \tag{66}$$

in Step 4 of the algorithm: It is easy to check that $(\hat{s}^X, \hat{s}^Z)$ defined in this way satisfy

$$\begin{aligned}\hat{s}^X &= \mathsf{Syn}(S^X, \widehat{E}_{\mathrm{gl}})\\ \hat{s}^Z &= \mathsf{Syn}(S^Z, \widehat{E}^*_{\mathrm{gl}})\end{aligned}$$

because of expressions (57) and since $\widehat{E}_{\mathrm{gl}}$ and $\widehat{E}^*_{\mathrm{gl}}$ have no support on $\{q_1, q_2\}$.

Having motivated the algorithm, let us next give an expression for the resulting final state when the algorithm is run on a corrupted cluster state.

Theorem 4.6. *Let E be a Pauli error on $\mathcal{C}$. Suppose we run Algorithm 2 on the input state $EW\left|0^{\mathcal{C}}\right\rangle$. Then the output is the Bell state $\Phi_{(\alpha,\beta)}$ where*

$$\begin{aligned}\alpha &= \left\langle\!\!\left\langle \{q_1,q_2\}, \mathrm{supp}(E^Z)\right\rangle\!\!\right\rangle \oplus \left\langle\!\!\left\langle \mathrm{supp}(E_{\mathrm{gl}}) \oplus \mathsf{MinMatch}_{\mathsf{T}_{\mathrm{cl,dec}}}\left(\partial_{\mathsf{T}_{\mathrm{cl,dec}}}(\mathrm{supp}(E_{\mathrm{gl}}))\right), \mathcal{L}_{\mathrm{cl},X}\right\rangle\!\!\right\rangle\\ \beta &= \left\langle\!\!\left\langle \{q_1,q_2\}, \mathrm{supp}(E^X)\right\rangle\!\!\right\rangle \oplus \left\langle\!\!\left\langle \mathrm{supp}(E^*_{\mathrm{gl}}) \oplus \mathsf{MinMatch}_{\mathsf{T}_{\mathrm{cl,dec^*}}}\left(\partial_{\mathsf{T}_{\mathrm{cl,dec^*}}}(\mathrm{supp}(E^*_{\mathrm{gl}}))\right), \mathcal{L}^*_{\mathrm{cl},Z}\right\rangle\!\!\right\rangle\ .\end{aligned}$$

Proof. Because m, m^* are equal to

$$\begin{aligned}m &= \mathsf{MinMatch}_{\mathsf{T}_{\mathrm{cl,dec}}}(s) = \mathsf{MinMatch}_{\mathsf{T}_{\mathrm{cl,dec}}}\left(\partial_{\mathsf{T}_{\mathrm{cl,dec}}}(\mathrm{supp}(E_{\mathrm{gl}}))\right)\\ m^* &= \mathsf{MinMatch}_{\mathsf{T}_{\mathrm{cl,dec^*}}}(s^*) = \mathsf{MinMatch}_{\mathsf{T}_{\mathrm{cl,dec^*}}}\left(\partial_{\mathsf{T}_{\mathrm{cl,dec^*}}}(\mathrm{supp}(E^*_{\mathrm{gl}})\right)\ ,\end{aligned}$$

see Step 3 of the algorithm and Lemma 4.5, the claim amounts to the statement that the output state is the Bell state $\Phi_{(\alpha,\beta)}$ where

$$\begin{aligned}\alpha &= \left\langle\!\!\left\langle \{q_1,q_2\}, \mathrm{supp}(E^Z)\right\rangle\!\!\right\rangle \oplus \left\langle\!\!\left\langle \mathrm{supp}(E_{\mathrm{gl}}) \oplus m, \mathcal{L}_{\mathrm{cl},X}\right\rangle\!\!\right\rangle\\ \beta &= \left\langle\!\!\left\langle \{q_1,q_2\}, \mathrm{supp}(E^X)\right\rangle\!\!\right\rangle \oplus \left\langle\!\!\left\langle \mathrm{supp}(E^*_{\mathrm{gl}}) \oplus m^*, \mathcal{L}^*_{\mathrm{cl},Z}\right\rangle\!\!\right\rangle\ .\end{aligned}$$

According to Lemma 4.3, the post-measurement state after Step 1 of Algorithm 2 is the Bell state $\left|\Phi_{(c_X,c_Z)}\right\rangle$ with

$$\begin{aligned}c_X &= \left\langle\!\!\left\langle a, \mathcal{L}_{\mathrm{cl},X} \cap \mathcal{A}\right\rangle\!\!\right\rangle \oplus \left\langle\!\!\left\langle x, \mathcal{L}_{\mathrm{cl},X} \cap \mathcal{B}\right\rangle\!\!\right\rangle \oplus \mathsf{Syn}(S^X, E)\\ c_Z &= \left\langle\!\!\left\langle a, \mathcal{L}^*_{\mathrm{cl},Z} \cap \mathcal{A}\right\rangle\!\!\right\rangle \oplus \left\langle\!\!\left\langle z, \mathcal{L}^*_{\mathrm{cl},Z} \cap \mathcal{B}\right\rangle\!\!\right\rangle \oplus \mathsf{Syn}(S^Z, E)\ .\end{aligned}$$

Since the algorithm applies the correction operation $Z^{\hat{c}_X} X^{\hat{c}_Z}$ in Step 6, the output of the algorithm is the Bell state $\left|\Psi_{(c_X \oplus \hat{c}_X, c_Z \oplus \hat{c}_Z)}\right\rangle$. It follows with the definition of $(\hat{c}_X, \hat{c}_Z)$ in Step 5, i.e.,

$$\begin{aligned}\hat{c}_X &= \left\langle\!\!\left\langle a, \mathcal{L}_{\mathrm{cl},X} \cap \mathcal{A}\right\rangle\!\!\right\rangle \oplus \left\langle\!\!\left\langle x, \mathcal{L}_{\mathrm{cl},X} \cap \mathcal{B}\right\rangle\!\!\right\rangle \oplus \hat{s}^X\\ \hat{c}_Z &= \left\langle\!\!\left\langle a, \mathcal{L}^*_{\mathrm{cl},Z} \cap \mathcal{A}\right\rangle\!\!\right\rangle \oplus \left\langle\!\!\left\langle z, \mathcal{L}^*_{\mathrm{cl},Z} \cap \mathcal{B}\right\rangle\!\!\right\rangle \oplus \hat{s}^Z\end{aligned}$$

that the output is the Bell state $\left|\Phi_{(\alpha,\beta)}\right\rangle$ where

$$\begin{aligned}\alpha &= \hat{s}^X \oplus \mathsf{Syn}(S^X, E)\\ \beta &= \hat{s}^Z \oplus \mathsf{Syn}(S^Z, E)\ .\end{aligned}$$

The claim now follows by combining the expressions (57), (58) for $\left(\mathsf{Syn}(S^X, E), \mathsf{Syn}(S^Z, E)\right)$ from Lemma 4.4 with the Definitions (66) of $(\hat{s}^X, \hat{s}^Z)$ used in Step (4) of the algorithm. □

We note that all theorems and lemmas in this section still hold if we replace the function $\mathsf{MinMatch}$ on $\mathsf{T}_{\mathrm{cl,dec}}$ and $\mathsf{T}_{\mathrm{cl,dec^*}}$ with any function on $\mathsf{V}^{\mathrm{int}}_{\mathrm{cl,dec}}$ and $\mathsf{V}^{\mathrm{int}}_{\mathrm{cl,dec^*}}$ with binary output. The fact that $\mathsf{MinMatch}$ produces a matching is – up to this point – only used to motivate the definition of $\hat{s}^X$ and $\hat{s}^Z$ in Algorithm 2. We will use this property of $\mathsf{MinMatch}$ in Section 5 to show that our protocol is robust against local stochastic noise.

5 Entanglement generation with local stochastic noise

In this section, we show that the entanglement generation protocol given by Algorithm 2 applied to the cluster state $W\left|0^{\mathcal{C}}\right\rangle$ is robust against local stochastic noise E of any noise strength p below some threshold p_0. In more detail, we consider a cluster state lattice $\mathcal{C}$ of the form $\mathcal{C}=\mathcal{C}[d\times d\times R]$ (with $d=O(\log R)$) and show that even when the ideal cluster state $W\left|0^{\mathcal{C}}\right\rangle$ is replaced by $EW\left|0^{\mathcal{C}}\right\rangle$, the resulting state has fidelity at least $1-O(p)$ with the Bell state Φ independently of R (see Corollary 5.4). In particular, the range R of the entanglement generation can be exponential in the surface code distance d while still generating constant-fidelity entanglement.

We proceed as follows: In Section 5.1, we construct upper bounds on the resilience functions $\mathsf{res}_{\mathcal{L}_{\mathrm{cl},X}}(p)$ and $\mathsf{res}_{\mathcal{L}^*_{\mathrm{cl},Z}}(p)$ of the recovery sets $\mathcal{L}_{\mathrm{cl},X}$ and $\mathcal{L}^*_{\mathrm{cl},Z}$. In Section 5.2, we then establish a lower bound on the success probability of the entanglement generation protocol using the results of Section 5.1.

5.1 Bounds on the resilience function for $\mathsf{T}_{\mathrm{cl,dec}}$ and $\mathsf{T}_{\mathrm{cl,dec}^*}$

Here we establish upper bounds on the resilience functions of the recovery sets $\mathcal{L}_{\mathrm{cl},X}$ and $\mathcal{L}^*_{\mathrm{cl},Z}$ for the decoding graphs $\mathsf{T}_{\mathrm{cl,dec}}$ and $\mathsf{T}_{\mathrm{cl,dec}^*}$ for small values of p. These upper bounds immediately give lower bounds on the success probability of the entanglement generation protocol, see Section 5.2.

Lemma 5.1. *Consider the decoding graph $\mathsf{T}_{\mathrm{cl,dec}}=(\mathsf{V}_{\mathrm{cl,dec}},\mathsf{E}_{\mathrm{cl,dec}})$ with set of internal vertices $\mathsf{V}^{\mathrm{int}}_{\mathrm{cl,dec}}$ defined on the lattice $\widetilde{\mathcal{C}}[d\times d\times R]$. Let $\mathcal{L}_{\mathrm{cl},X}\subset\mathsf{E}_{\mathrm{cl,dec}}$ be the recovery set introduced in Section 4.3.2. Then the resilience function $\mathsf{res}_{\mathcal{L}_{\mathrm{cl},X}}$ satisfies*

$$\mathsf{res}_{\mathcal{L}_{\mathrm{cl},X}}(p)\leq\left(2402+100d(R-1)\left(10\sqrt{p}\right)^{d-2}\right)\cdot p \tag{67}$$

for any $p\in[0,\frac{1}{400}]$.

Proof. Let $L\in Z_\circ(\mathsf{T}_{\mathrm{cl,dec}})$ be a simple closed loop. Then it is clear from the definition of $\mathcal{L}_{\mathrm{cl},X}$ that L and $\mathcal{L}_{\mathrm{cl},X}$ have no overlap, and in particular, $\langle\!\langle L,\mathcal{L}_{\mathrm{cl},X}\rangle\!\rangle=0$. This implies that simple closed loops do not contribute to the resilience function $\mathsf{res}_{\mathcal{L}_{\mathrm{cl},X}}(p)$ (see Definition (7)).

To describe the set $Z_{\mathrm{ext}}(\mathsf{T}_{\mathrm{cl,dec}})$ of simple paths connecting external vertices through internal vertices, we partition the set $\mathsf{V}^{\mathrm{ext}}_{\mathrm{cl,dec}}=\mathsf{V}^{\mathrm{ext,left}}_{\mathrm{cl,dec}}\cup\mathsf{V}^{\mathrm{ext,right}}_{\mathrm{cl,dec}}$ of external vertices into the set $\mathsf{V}^{\mathrm{ext,left}}_{\mathrm{cl,dec}}$ of vertices forming the "left" boundary of the decoding graph $\mathsf{T}_{\mathrm{cl,dec}}$ and the set $\mathsf{V}^{\mathrm{ext,right}}_{\mathrm{cl,dec}}$ of remaining external vertices forming the "right", "front", and "back" boundaries. We label the external vertices in $\mathsf{V}^{\mathrm{ext}}_{\mathrm{cl,dec}}$ as

$$\begin{aligned}\mathsf{V}^{\mathrm{ext,left}}_{\mathrm{cl,dec}}&=\left\{u_j^k\mid 1\leq j\leq d-1,k\in\left\{0,\frac{R+1}{2}\right\}\right\}\cup\left\{u_j^k\mid 0\leq j\leq d-1,1\leq k\leq\frac{R-1}{2}\right\}\\ \mathsf{V}^{\mathrm{ext,right}}_{\mathrm{cl,dec}}&=\left\{v_{i,j}^k\mid 1\leq i\leq d-1,0\leq j\leq i,k\in\left\{0,\frac{R+1}{2}\right\}\right\}\cup\left\{v_j^k\mid 0\leq j\leq d-1,1\leq k\leq\frac{R-1}{2}\right\}\ ,\end{aligned}$$

see Fig. 6. Written out, the external vertices are

$$u_j^k=\begin{cases}(0,2j,1) & \text{for } k=0,\ 1\leq j\leq d-1\\ (0,2j,2k) & \text{for } 1\leq k\leq\frac{R-1}{2},\ 0\leq j\leq d-1\\ (0,2j,R) & \text{for } k=\frac{R+1}{2},\ 1\leq j\leq d-1\end{cases} \tag{68}$$

$$v_{i,j}^k=\begin{cases}(2i,2j,1) & \text{for } k=0,\ 1\leq i\leq d-1,\ 0\leq j\leq i\\ (2i,2j,R) & \text{for } k=\frac{R+1}{2},\ 1\leq i\leq d-1,\ 0\leq j\leq i\end{cases}$$

$$v_j^k=(2d,2j,2k)\qquad\text{for } 1\leq k\leq\frac{R-1}{2},\ 0\leq j\leq d-1\ . \tag{69}$$

It is clear from the definition of $\mathsf{T}_{\text{cl,dec}}$ that a simple path $P \in Z_{\text{ext}}(\mathsf{T}_{\text{cl,dec}})$ satisfies $\langle\!\langle P, \mathcal{L}_{\text{cl},X} \rangle\!\rangle = 1$ if and only if P connects an external vertex $u \in \mathsf{V}_{\text{cl,dec}}^{\text{ext,left}}$ to another external vertex $v \in \mathsf{V}_{\text{cl,dec}}^{\text{ext,right}}$. Here we say such a path P (through internal vertices) starts at u and ends at v. Let us denote the set of all simple paths of length ℓ which start at $u_j^k \in \mathsf{V}_{\text{cl,dec}}^{\text{ext,left}}$ and end at $v \in \mathsf{V}_{\text{cl,dec}}^{\text{ext,right}}$ through internal vertices by $\Delta(u_j^k, \ell)$. Then we can write the set of all simple paths of length ℓ in $Z_{\text{ext}}(\mathsf{T}_{\text{cl,dec}})$ satisfying $\langle\!\langle P, \mathcal{L}_{\text{cl},X} \rangle\!\rangle = 1$ as

$$\{P \in Z_{\text{ext}}(\mathsf{T}_{\text{cl,dec}}) \mid |P| = \ell \text{ and } \langle\!\langle P, \mathcal{L}_{\text{cl},X} \rangle\!\rangle = 1\} = \bigcup_{u \in \mathsf{V}_{\text{cl,dec}}^{\text{ext,left}}} \Delta(u, \ell) \ . \tag{70}$$

Note that $\Delta(u, \ell)$ and $\Delta(u', \ell')$ are disjoint for $(u, \ell) \neq (u', \ell')$ since the corresponding paths either start at different vertices or have different lengths. We also note that $\Delta(u, \ell)$ is empty for sufficiently large ℓ since we are considering simple paths and the decoding graph $\mathsf{T}_{\text{cl,dec}}$ is finite. It follows from the definition (7) of the resilience function and from (70) that

$$\begin{aligned} \mathsf{res}_{\mathcal{L}_{\text{cl},X}}(p) &= \sum_{\ell=1}^{\infty} \binom{\ell}{\lceil \ell/u \rceil} \cdot \left| \bigcup_{u \in \mathsf{V}_{\text{cl,dec}}^{\text{ext,left}}} \Delta(u, \ell) \right| \cdot p^{\lceil \ell/2 \rceil} \\ &= \sum_{u \in \mathsf{V}_{\text{cl,dec}}^{\text{ext,left}}} \sum_{\ell=L_u^{\min}}^{L_u^{\max}} \binom{\ell}{\lceil \ell/2 \rceil} \cdot |\Delta(u, \ell)| \cdot p^{\lceil \ell/2 \rceil} \ . \end{aligned} \tag{71}$$

Here $L_u^{\min}$ and $L_u^{\max}$ are the minimal and maximal lengths of paths in $\bigcup_{\ell=1}^{\infty} \Delta(u, \ell)$ for $u \in \mathsf{V}_{\text{cl,dec}}^{\text{ext,left}}$.

We rewrite the expression (71) by partitioning the set $\mathsf{V}_{\text{cl,dec}}^{\text{ext,left}}$ as follows. Let us define

$$\mathsf{V}_{\text{cl,dec}}^{\text{ext,left}}(s) = \left\{ u \in \mathsf{V}_{\text{cl,dec}}^{\text{ext,left}} \mid L_u^{\min} = s \right\} \qquad \text{for} \qquad s \geq 1 \ .$$

In other words, $\mathsf{V}_{\text{cl,dec}}^{\text{ext,left}}(s)$ is the set of external vertices u in $\mathsf{V}_{\text{cl,dec}}^{\text{ext,left}}$ such that the minimal distance to $\mathsf{V}_{\text{cl,dec}}^{\text{ext,right}}$ is s in terms of the length of paths connecting u and $\mathsf{V}_{\text{cl,dec}}^{\text{ext,right}}$. It is clear from the definition of $\mathsf{T}_{\text{cl,dec}}$ that the set $\mathsf{V}_{\text{cl,dec}}^{\text{ext,left}}(s)$ is empty for all $s > d$. Furthermore, the sets $\{\mathsf{V}_{\text{cl,dec}}^{\text{ext,left}}(s)\}_{s=1}^{d}$ are pairwise disjoint and their union is $\mathsf{V}_{\text{cl,dec}}^{\text{ext,left}}$. Thus we can write the expression (71) as

$$\mathsf{res}_{\mathcal{L}_{\text{cl},X}}(p) = \sum_{s=1}^{d} A_s \qquad \text{where} \qquad A_s = \sum_{u \in \mathsf{V}_{\text{cl,dec}}^{\text{ext,left}}(s)} \sum_{\ell=s}^{L_u^{\max}} \binom{\ell}{\lceil \ell/2 \rceil} \cdot |\Delta(u, \ell)| \cdot p^{\lceil \ell/2 \rceil} \ . \tag{72}$$

We first compute A_1. It is clear from the definition of $\mathsf{T}_{\text{cl,dec}}$ that u_1^0 and $u_1^{\frac{R+1}{2}}$ are the only vertices in $\mathsf{V}_{\text{cl,dec}}^{\text{ext,left}}(1)$, i.e.,

$$\mathsf{V}_{\text{cl,dec}}^{\text{ext,left}}(1) = \left\{ u_1^0, u_1^{\frac{R+1}{2}} \right\} \ . \tag{73}$$

In addition, the set of all simple paths through internal vertices starting at either u_1^0 or $u_1^{\frac{R+1}{1}}$ and ending at any external vertex in $\mathsf{V}_{\text{cl,dec}}^{\text{ext,right}}$ consists of only two length-1 paths, the path $\left(\{u_1^0, v_{1,1}^0\}\right)$ and the path $\left(\left\{u_1^{\frac{R+1}{2}}, v_{1,1}^{\frac{R+1}{2}}\right\}\right)$. In particular, we have

$$L_{u_1^0}^{\max} = L_{u_1^{\frac{R+1}{2}}}^{\max} = 1 \ . \tag{74}$$

According to (73) and (74) with the definition of A_1 from (72), we have

$$\begin{aligned} A_1 &= \binom{1}{\lceil 1/2 \rceil} \cdot \left|\Delta(u_1^0, 1)\right| \cdot p^{\lceil 1/2 \rceil} + \binom{1}{\lceil 1/2 \rceil} \cdot \left|\Delta(u_1^{\frac{R+1}{2}}, 1)\right| \cdot p^{\lceil 1/2 \rceil} \\ &= 2 \cdot p \ . \end{aligned} \tag{75}$$

Next, we consider A_d. It is clear from the definition of $\mathsf{T}_{\text{cl,dec}}$ that $u_j^k \notin \mathsf{V}_{\text{cl,dec}}^{\text{ext,left}}(d)$ for $1 \leq j \leq d-1$ and $k \in \left\{0, \frac{R+1}{2}\right\}$. This gives the upper bound

$$\begin{aligned} \left|\mathsf{V}_{\text{cl,dec}}^{\text{ext,left}}(d)\right| &\leq \left|\mathsf{V}_{\text{cl,dec}}^{\text{ext,left}} \setminus \left\{u_j^k \mid 1 \leq j \leq d-1, k \in \left\{0, \frac{R+1}{2}\right\}\right\}\right| \\ &= d \cdot \left(\frac{R-1}{2}\right) . \end{aligned} \tag{76}$$

The fact that the decoding graph $\mathsf{T}_{\text{cl,dec}}$ has vertices of degree at most 6 implies that

$$|\Delta(u, \ell)| \leq 5^\ell \qquad \text{for} \qquad u \in \mathsf{V}_{\text{cl,dec}}^{\text{ext,left}} . \tag{77}$$

Combining (76), (77) and the fact that $\binom{\ell}{\lceil \ell/2 \rceil} \leq 2^\ell$ and $p^{\lceil \ell/2 \rceil} \leq p^{\ell/2}$ with the definition (72) of A_s with $s = d$, we obtain

$$\begin{aligned} A_d &\leq d \cdot \left(\frac{R-1}{2}\right) \sum_{\ell=d}^{\infty} 2^\ell \cdot 5^\ell \cdot p^{\ell/2} \\ &= d \cdot \left(\frac{R-1}{2}\right) \sum_{\ell=d}^{\infty} (10\sqrt{p})^\ell \\ &= d \cdot \left(\frac{R-1}{2}\right) \frac{q^d}{1-q} \qquad \text{where} \qquad q := 10\sqrt{p} . \end{aligned} \tag{78}$$

Here the geometric series converges since the assumption $0 \leq p \leq \frac{1}{400}$ implies $0 \leq q \leq \frac{1}{2}$.

It remains to calculate an upper bound of $\sum_{s=2}^{d-1} A_s$. Straightforward counting yields

$$\left|\mathsf{V}_{\text{cl,dec}}^{\text{ext,left}}(s)\right| \leq 2 \cdot s \qquad \text{for} \qquad 2 \leq s \leq d-1 .$$

It follows from an analogous argument as for the upper bound on A_d that

$$\begin{aligned} \sum_{s=2}^{d-1} A_s &\leq \sum_{s=2}^{d-1} \left(2 \cdot s \sum_{\ell=s}^{\infty} 2^\ell \cdot 5^\ell \cdot p^{\ell/2}\right) \\ &= \sum_{s=2}^{d-1} \left(2 \cdot s \cdot \frac{q^s}{1-q}\right) \qquad \text{where} \qquad q := 10\sqrt{p} \\ &\leq \frac{4q^2}{(1-q)^2} + \frac{2q^3}{(1-q)^3} . \end{aligned} \tag{79}$$

Here again, the geometric- and the arithmetico-geometric series converge since $0 \leq q \leq \frac{1}{2}$. Combining (75), (78) and (79), we have

$$\begin{aligned} \mathsf{res}_{\mathcal{L}_{\text{cl},X}}(p) &= \sum_{s=1}^{d} A_s \\ &\leq 2p + \frac{4q^2}{(1-q)^2} + \frac{2q^3}{(1-q)^3} + d \cdot \frac{R-1}{2} \cdot \frac{q^d}{1-q} \\ &= \left[2 + \frac{400}{(1-q)^2} + \frac{200q}{(1-q)^3} + 50d(R-1) \cdot \frac{(10\sqrt{p})^{d-2}}{1-q}\right] \cdot p . \end{aligned} \tag{80}$$

Here we used the definition of q for the last equality. We have $q \leq \frac{1}{2}$ and $\frac{1}{1-q} \leq 2$, hence we obtain the desired bound (67) from (80). □

The resilience function $\mathsf{res}_{\mathcal{L}_{\text{cl},Z}^*}(p)$ of the dual decoding graph and the recovery set $\mathcal{L}_{\text{cl},Z}^*$ can be upper bounded in a similar manner.

Lemma 5.2. *Consider the dual decoding graph $\mathsf{T}_{\mathrm{cl,dec}^*} = (\mathsf{V}_{\mathrm{cl,dec}^*}, \mathsf{E}_{\mathrm{cl,dec}^*})$ with set of internal vertices $\mathsf{V}^{\mathrm{int}}_{\mathrm{cl,dec}^*}$ defined on the dual lattice $\widetilde{\mathcal{C}}^*[d \times d \times R]$. Let $\mathcal{L}^*_{\mathrm{cl},Z} \subset \mathsf{E}_{\mathrm{cl,dec}^*}$ be the recovery set introduced in Section 4.3.2. Then the resilience function $\mathsf{res}_{\mathcal{L}^*_{\mathrm{cl},Z}}$ satisfies*

$$\mathsf{res}_{\mathcal{L}^*_{\mathrm{cl},Z}}(p) \leq \left[2400 + 100d(R+1)(10\sqrt{p})^{d-2}\right] \cdot p \ . \tag{81}$$

for any $p \in [0, \frac{1}{400}]$.

The proof mirrors that of Lemma 5.1 and is given in Appendix C for completeness.

5.2 A lower bound on the success probability of the protocol

Now we state our main result, a lower bound on the success probability of our entanglement generation protocol. Here the success probability (denoted $\nu_{(0,0)}$ in the following) is the probability (taken over the random choice of local stochastic error E as well as the measurements outcomes) that the final state of the protocol is the Bell state $\Phi = \Phi_{(0,0)}$. Note that this quantity is in one-to-one-correspondence with the fidelity of the (average) output state with the Bell state Φ. This is because the protocol always produces one of the four orthogonal Bell states.

Theorem 5.3 (Entanglement generation with local stochastic noise). *Let $d \geq 2$, and let $R \geq 3$ be an odd integer. Suppose we run Algorithm 2 on a noisy cluster state $EW\left|0^{\mathcal{C}}\right\rangle$ on a lattice $\mathcal{C}[d \times d \times R]$, where the cluster state is corrupted by a local stochastic error $E \sim \mathcal{N}(p)$ with $p \in [0, \frac{1}{400}]$. Then there is a probability distribution $\nu = (\nu_{(0,0)}, \nu_{(0,1)}, \nu_{(1,0)}, \nu_{(1,1)})$ such that the following holds:*

(i) The resulting output state is the two-qubit Bell state $\left|\Phi_{(\alpha,\beta)}\right\rangle$ with probability $\nu_{(\alpha,\beta)}$ for every pair $(\alpha, \beta) \in \{0,1\}^2$.

(ii) The success probability $\nu_{(0,0)}$ of the entanglement generation protocol satisfies

$$\nu_{(0,0)} \geq 1 - \left[4806 + 200dR(10\sqrt{p})^{d-2}\right] \cdot p \ . \tag{82}$$

Proof. Due to Lemma 4.3, it suffices to establish an upper bound on $1 - \nu_{(0,0)}$. Let us introduce the following four random variables:

$$\begin{aligned}
A_Z &:= \left\langle\!\left\langle \{q_1, q_2\}, \mathrm{supp}(E^Z) \right\rangle\!\right\rangle \\
B_Z &:= \left\langle\!\left\langle \mathrm{supp}(E_{\mathrm{gl}}) \oplus \mathsf{MinMatch}_{\mathsf{T}_{\mathrm{cl,dec}}}(\partial_{\mathsf{T}_{\mathrm{cl,dec}}} \mathrm{supp}(E_{\mathrm{gl}})), \mathcal{L}_{\mathrm{cl},X} \right\rangle\!\right\rangle \\
A_X &:= \left\langle\!\left\langle \{q_1, q_2\}, \mathrm{supp}(E^X) \right\rangle\!\right\rangle \\
B_X &:= \left\langle\!\left\langle \mathrm{supp}(E^*_{\mathrm{gl}}) \oplus \mathsf{MinMatch}_{\mathsf{T}_{\mathrm{cl,dec}^*}}(\partial_{\mathsf{T}_{\mathrm{cl,dec}^*}} \mathrm{supp}(E^*_{\mathrm{gl}})), \mathcal{L}^*_{\mathrm{cl},Z} \right\rangle\!\right\rangle \ .
\end{aligned} \tag{83}$$

Then Theorem 4.6 translates to

$$\nu_{(0,0)} = \Pr[(A_Z, B_Z) \in \{(0,0),(1,1)\} \text{ and } (A_X, B_X) \in \{(0,0),(1,1)\}] \ . \tag{84}$$

The union bound gives

$$1 - \nu_{(0,0)} \leq \Pr[(A_Z, B_Z) \in \{(0,1),(1,0)\}] + \Pr[(A_X, B_X) \in \{(0,1),(1,0)\}] \ . \tag{85}$$

Using the union bound on the first term of (85), we have

$$\begin{aligned}
\Pr[(A_Z, B_Z) \in \{(0,1),(1,0)\}] &\leq \Pr[(A_Z, B_Z) = (0,1)] + \Pr[(A_Z, B_Z) = (1,0)] \\
&\leq \Pr[B_Z = 1] + \Pr[A_Z = 1] \ .
\end{aligned}$$

Applying the same reasoning to the second term of (85) yields

$$1 - \nu_{(0,0)} \leq \Pr[A_X = 1] + \Pr[A_Z = 1] + \Pr[B_X = 1] + \Pr[B_Z = 1] \ . \tag{86}$$

By the definition of A_X and (bi-)linearity of $\langle\!\langle \cdot, \cdot \rangle\!\rangle$ together with the union bound, we have

$$\begin{aligned}\Pr[A_X = 1] &\leq \Pr\big[\langle\!\langle \{q_1\}, \mathrm{supp}(E^Z) \rangle\!\rangle \oplus \langle\!\langle \{q_2\}, \mathrm{supp}(E^Z) \rangle\!\rangle = 1\big] \\ &\leq \Pr\big[\langle\!\langle \{q_1\}, \mathrm{supp}(E^Z) \rangle\!\rangle = 1\big] + \Pr\big[\langle\!\langle \{q_2\}, \mathrm{supp}(E^Z) \rangle\!\rangle = 1\big] \\ &\leq p + p = 2p \end{aligned} \tag{87}$$

where we used that $E^Z \sim \mathcal{N}(p)$ is local stochastic noise with the same parameter as E. In a similar manner, we obtain

$$\Pr[A_Z = 1] = 2p\ . \tag{88}$$

We also have

$$\begin{aligned}\Pr[B_Z = 1] &= \Pr\big[\langle\!\langle \mathrm{supp}(E_{\mathrm{gl}}) \oplus \mathsf{MinMatch}_{\mathsf{T}_{\mathrm{cl,dec}}}(\partial_{\mathsf{T}_{\mathrm{cl,dec}}} \mathrm{supp}(E_{\mathrm{gl}})), \mathcal{L}_{\mathrm{cl},X} \rangle\!\rangle = 1\big] \\ &\leq \mathsf{res}_{\mathcal{L}_{\mathrm{cl},X}}(p) \\ &\leq \big[2402 + 100d(R-1)(10\sqrt{p})^{d-2}\big] \cdot p \end{aligned} \tag{89}$$

where the definition of B_Z, the fact that $E_{\mathrm{gl}} \sim \mathcal{N}(p)$, Proposition 2.3 and Lemma 5.1 are used. By similar reasoning, we get

$$\begin{aligned}\Pr[B_X = 1] &= \Pr\big[\langle\!\langle \mathrm{supp}(E^*_{\mathrm{gl}}) \oplus \mathsf{MinMatch}_{\mathsf{T}_{\mathrm{cl,dec^*}}}(\partial_{\mathsf{T}_{\mathrm{cl,dec^*}}} \mathrm{supp}(E^*_{\mathrm{gl}})), \mathcal{L}^*_{\mathrm{cl},Z} \rangle\!\rangle = 1\big] \\ &\leq \mathsf{res}_{\mathcal{L}^*_{\mathrm{cl},Z}}(p) \\ &\leq \big[2400 + 100d(R+1)(10\sqrt{p})^{d-2}\big] \cdot p\ , \end{aligned} \tag{90}$$

see Lemma 5.2. Combining inequalities (87), (88), (89) and (90) together with (86), we obtain the claim (82). ☐

Theorem 5.3 immediately implies the following corollary. It shows that for any sufficiently small constant noise-strength, a constant-fidelity Bell state can be established over a distance R which is exponential in d.

Corollary 5.4. *Let $p \in (0, \frac{1}{5006}]$, $d \in \mathbb{N}$ with $d \geq 3$ and $R \in \mathbb{N}$ be odd with $R \geq 3$. Suppose we run Algorithm 2 on the noisy cluster state $EW\left|0^{\mathcal{C}}\right\rangle$ with local stochastic noise $E \sim \mathcal{N}(p)$ in the lattice $\mathcal{C}\left[d \times d \times R\right]$ with*

$$R \leq \frac{1}{d}\left(\frac{1}{10\sqrt{p}}\right)^{d-2}\ . \tag{91}$$

Then the probability $\nu_{(0,0)}$ of successful entanglement generation satisfies

$$\nu_{(0,0)} \geq 1 - 5006p\ .$$

Proof. Due to the condition $p \in (0, \frac{1}{5006}] \subset [0, \frac{1}{400}]$, the inequality (82) holds. Assuming that R satisfies the upper bound (91), the claim follows. ☐

6 Limits on low-latency entanglement generation

Here we establish limits on fault-tolerant long-range entanglement generation using adaptive shallow (i.e., constant-depth) circuits along a line of repeater stations. Such circuits capture low-latency schemes where the entanglement is generated in a constant amount of time (up to local corrections). We show that any such scheme which is resilient to arbitrary local stochastic noise of strength below some threshold can only establish constant-fidelity entanglement up to a certain distance R: We show that R

must be upper bounded by a function which is exponential in the number m of qubits at each repeater station.

In Section 6.1, we establish a general converse bound for low-latency entanglement generation, applicable to all protocols realized by constant-depth adaptive circuits. In Section 6.2, we establish a converse bound for any scheme based on the cluster state $W\left|0^{\mathcal{C}}\right\rangle$ which uses the same measurement pattern and syndrome information as Algorithm 2, but possibly different classical decoding strategies. We discuss how these two converse bounds compare to each other and to our achievability results for Algorithm 2 from Section 5.2.

6.1 A general converse bound for low-latency entanglement generation

In the following, we consider constant-depth protocols for entanglement generation across a line of R site with m qubits per site (i.e., per repeater). Constant-depth here means that the entanglement generation protocol can be written as a circuit of depth independent of (m, R) composed of single-qubit gates and measurements, and two-qubit gates between any pair of qubits located at the same or two neighboring repeaters. Throughout, we consider adaptive circuits, i.e., circuits that apply unitaries which are classically controlled by (functions of) measurement results. Classical computations (i.e., function evaluations) to determine the unitaries which are applied are not counted in the definition of circuit depth. Furthermore, for our converse bound, it actually suffices to consider the circuit depth "between repeaters", i.e., gate sequences within a repeater station can be assumed to contribute only a single gate layer to the overall circuit depth Δ.

We assume that the considered protocols generate entanglement between a qubit q_1 at the first repeater ($u_3 = 1$) and a qubit q_2 at the last repeater ($u_3 = R$). The main result of this section is the following converse. It shows that long-range entanglement cannot be generated fault-tolerantly by constant-depth adaptive circuits if the distance is more than exponential in the number m of qubits at each repeater.

Corollary 6.1. *Let π be a depth-Δ circuit for entanglement generation between two qubits q_1 and q_2 at distance R using m qubits per repeater. Suppose there is some constant threshold p_0 such that π produces the state $\rho_{q_1q_2}$ with fidelity $\langle\Phi|\rho_{q_1q_2}|\Phi\rangle$ at least $\frac{1}{2}(1+e^{-1}) \approx 0.684$ for any local stochastic noise of strength $p \leq p_0$. Then*

$$R \leq \left(\frac{3}{4p_0^2}\right)^{\Delta m} . \tag{92}$$

To prove Corollary 6.1, the following general no-go result for entanglement generation with limited-depth quantum circuits is needed:

Theorem 6.2. *Let π be a circuit of depth Δ for distance-R entanglement generation using m qubits per repeater. Let $p \in (0,1)$ be arbitrary. Then there is a local stochastic noise model of strength p acting on the circuit such that π produces a state $\rho_{q_1q_2}$ of the form*

$$\rho_{q_1q_2} = (1-p_{\mathrm{fail}})\rho^{\mathrm{rest}}_{q_1q_2} + p_{\mathrm{fail}}\rho^{\mathrm{sep}}_{q_1q_2} ,$$

where $\rho^{\mathrm{rest}}_{q_1q_2}$ is some state, $\rho^{\mathrm{sep}}_{q_1q_2}$ is a separable state, and $1 - e^{-R(4p^2/3)^{\Delta m}} \leq p_{\mathrm{fail}} \leq 1$.

Proof of Corollary 6.1. Since any two-qubit separable state $\rho^{\mathrm{sep}}_{q_1q_2}$ overlaps with the Bell state Φ at most $\langle\Phi|\rho_{q_1q_2}|\Phi\rangle \leq \frac{1}{2}$, it follows that

$$\begin{aligned}\langle\Phi|\rho_{q_1q_2}|\Phi\rangle &= (1-p_{\mathrm{fail}})\langle\Phi|\rho^{\mathrm{rest}}_{q_1q_2}|\Phi\rangle + p_{\mathrm{fail}}\langle\Phi|\rho^{\mathrm{sep}}_{q_1q_2}|\Phi\rangle \\ &\leq 1 - \frac{p_{\mathrm{fail}}}{2} \\ &\leq \frac{1}{2}\left(1 + e^{-R(4p^2/3)^{\Delta m}}\right) .\end{aligned}$$

Then the claim (92) follows from the assumption $\langle\Phi|\rho_{q_1q_2}|\Phi\rangle \geq \frac{1}{2}(1+e^{-1})$. □

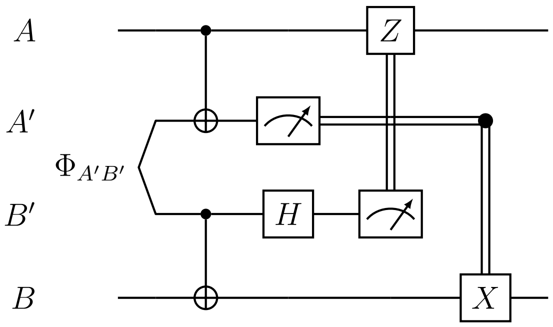

Figure 7: Gate-teleportation [7] based protocol for realizing a two-qubit CNOT_{AB} gate between two sites, see also [4, 10]. The circuit using a Bell state $\Phi_{A'B'}$ as a resource, as well as local operations and measurements.

Reformulated, Theorem 6.2 implies that any protocol π that can withstand arbitrary local stochastic noise of strength p on the initial state and still succeeds (i.e., produces the Bell pair Φ) with probability p_{succ} must satisfy the relationship

$$R \leq (3/(4p^2))^{\Delta m} \log\left(1/p_{\text{succ}}\right) \ .$$

In particular, any protocol that produces constant-fidelity entanglement for any error strength below some threshold can at most reach a distance exponential in m.

The proof of Theorem 6.2 proceeds by an intermediate step, where we consider bipartite-entanglement-based protocols. To describe this in detail, let π be a distance-R-entanglement generation protocol using m qubits per repeater which can be implemented by a circuit of depth Δ.

We assume without loss of generality that the only two-qubit gates that π uses between neighboring sites are CNOT-gates. Note that each two-qubit unitary can be realized by a simple circuit consisting of at most three CNOT gates and (arbitrary) single-qubit gates [13]. Each such CNOT-gate can be realized by a gate-teleportation based circuit as shown in Fig. 7. This circuit consumes a shared resource Bell state Φ. We note that the CNOT-implementing circuit uses only local gates (where locality is with respect to the bipartition, i.e., we allow two-qubit gates at the "source" and "target" sites), local measurements, and classical communication. In particular, this shows that the CNOT gate can be implemented using a Bell state Φ and LOCC-operations only.

Applying this substitution to every CNOT-gate of π which acts between neighboring repeaters, we obtain a bipartite-entanglement-based protocol π' which has the following properties:

(i) The protocol π' uses a resource state $(\Phi^{\otimes t})_{A^tB^t}$ with $t \leq \Delta m$ copies of Φ shared between any neighboring pair (S_j, S_{j+1}) of sites. Here t is the maximal number of two-qubit CNOT gates executed between two neighboring sites.

(ii) The protocol π' is an LOCC-operation between $S_1 : S_2 : \cdots : S_R$.

(iii) Local stochastic noise on π' is equivalent to local stochastic noise on π. This follows from the fact that the substitution rule in Fig. 7 is local by suitably commuting errors as in the proof of [1, Lemma 11]. In fact, we only need the following special version: A local stochastic error $E \sim \mathcal{N}(p)$ of strength p acting on the t qubits $A^t = A_1 \cdots A_t$ (i.e., one half of the Bell states) in the resource state $(\Phi^{\otimes t})_{A^tB^t}$ followed by an error-free execution of π' is equivalent to executing π with local stochastic noise of strength $\sqrt{p}$. This is because in the circuit of Fig. 7, a single-qubit Y error on qubit A' is equivalent to a two-qubit error Z_AX_B after the CNOT, whereas single-qubit X- and Z-errors on A' become single-qubit errors X_B and Z_A, respectively.

The following lemma gives an upper bound on such an entanglement-based protocol π' for long-range entanglement generation.

Lemma 6.3. *Consider R sites $S_1, \ldots, S_R$ of the form*

$$S_j = \begin{cases} A_1C_1 & \text{for } j = 1 \\ A_jB_jC_j & \text{for } 1 < j < R \\ B_RC_R & \text{for } j = R \ , \end{cases}$$

where $A_j \cong B_j \cong (\mathbb{C}^2)^{\otimes t}$ for each j. Let π' be a constant-depth adaptive quantum circuit that acts on $S_1 \cdots S_R$ and whose output state is on C_1C_R. Suppose π' satisfies properties (i) *and* (ii)*.*

Assume that π' is an LOCC-operation with respect to the multipartition

$$S_1 : S_2 : \cdots : S_R \ .$$

Suppose we apply π' to an initial state of the form

$$\rho^{\text{initial}}_{S_1\cdots S_R} := \left(\bigotimes_{j=1}^{R-1} (|\Phi\rangle\langle\Phi|^{\otimes t})_{A_jB_{j+1}} \right) \otimes \bigotimes_{j=1}^{R} \rho_{C_j} \tag{93}$$

where $\Phi \in \mathbb{C}^2 \otimes \mathbb{C}^2$ is the two-qubit Bell state, and $\rho_{C_1}, \ldots, \rho_{C_R}$ are arbitrary. Then, for any $p \in (0, 1)$, there is a local stochastic error $E \sim \mathcal{N}(p)$ acting on the systems $A^R = (A_1, \ldots, A_R)$ of the initial state (93) *such that the final state $\rho^{\text{final}}_{C_1C_R}$ is of the form*

$$\rho^{\text{final}}_{C_1C_R} = (1 - p_{\text{fail}})\rho^{\text{final,rest}}_{C_1C_R} + p_{\text{fail}}\rho^{\text{final,sep}}_{C_1C_R} \tag{94}$$

where $\rho^{\text{final,sep}}_{C_1C_R}$ is a separable state (with respect to the bipartition $C_1 : C_R$), $\rho^{\text{final,rest}}_{C_1C_R}$ is some state, and

$$p_{\text{fail}} \geq 1 - e^{-R(4p/3)^t} \ .$$

Proof. This statement immediately follows from the observation that a protocol π' can only convert preexisting entanglement in the initial state $\rho^{\text{initial}}_{S_1\cdots S_R}$ to end up being on systems $C_1 : C_R$. Furthermore, the initial state contains bipartite entanglement across sites only between the pair of systems A_jB_{j+1}, for $j = 1, \ldots, R-1$. If this shared entanglement is lost due to noise then the state on C_1C_R remains separable throughout the execution of the (ideal) protocol.

For concreteness, assume that the depolarizing noise channel

$$\mathcal{E}_p(\rho) = (1-p)\rho + p \operatorname{tr}(\rho) I/2$$

with $0 < p \leq 1$ is applied to each system A_j before application of the (ideal) protocol π'. That is, we consider a noisy initial state of the form

$$\rho^{\text{initial, noisy}}_{S_1\cdots S_R} := \left(\bigotimes_{j=1}^{R-1} \bigotimes_{k=1}^{t} ((\mathcal{E}_p)_{A_j^{(k)}} \otimes \mathsf{id}_{B_{j+1}^{(k)}})(|\Phi\rangle\langle\Phi|_{A_j^{(k)}B_{j+1}^{(k)}}) \right) \otimes \bigotimes_{j=1}^{R} \rho_{C_j} \ .$$

For $p \in (0, 1)$, we argue that this state can be written as a convex combination

$$\rho^{\text{initial, noisy}}_{S_1\cdots S_R} = p_0\rho_{S_1\cdots S_R} + \sum_{j=1}^{R-1} p_j \rho^{(j)}_{S_1\cdots S_j:S_{j+1}\cdots S_R}$$

of a certain state $\rho_{S_1\ldots S_R}$ and states $\rho^{(j)}_{S_1\cdots S_j:S_{j+1}\cdots S_R}$ that are separable across bipartitions of the form $S_1 \cdots S_j : S_{j+1} \cdots S_R$, for $j \in [R-1]$ where

$$\begin{aligned} p_0 &\leq (1 - p^t)^R \\ &\leq e^{-p^t R} \ , \end{aligned} \tag{95}$$

where we used the inequality $(1-x)^r \leq e^{-xr}$. In particular, since each reduced state $\rho^{(j)}_{S_1 S_R}$ is separable, Eq. (95) and the fact that the (ideal) protocol π' is LOCC imply that the final state is of the form in Eq. (94) with $p_{\text{fail}} \geq 1 - e^{-p^t R}$. The claim follows from this because the depolarizing channel can be written as $\mathcal{E}_p(\rho) = \left(1 - \frac{3}{4}p\right)\rho + \frac{p}{4} X\rho X^\dagger + \frac{p}{4} Y\rho Y^\dagger + \frac{p}{4} Z\rho Z^\dagger$, i.e., is equivalent to applying each Pauli with probability $p/4$. Thus applying $\mathcal{E}_p$ to each system A_j amounts to having a local stochastic error $E \sim \mathcal{N}(3p/4)$. (Substituting p by $4p/3$ gives the claim.)

Note that the depolarizing channel $\mathcal{E}_p$ is entanglement-breaking for any $p \geq 2/3$, see [12]. Eq. (95) is a consequence of the more trivial fact that for any $p \in (0,1)$, the channel $\mathcal{E}_p$ is a convex combination of the identity channel id and the depolarizing channel $\mathcal{E}_1$ which is entanglement-breaking, that is, we have

$$\mathcal{E}_p(\rho) = (1-p)\ \mathsf{id}(\rho) + p\ \mathcal{E}_1(\rho)\ .$$

With $p_0 := 1-p$, $p_1 := p$ and the convention $\mathcal{E}^0 = \mathsf{id}$, this implies that for every $j \in [R-1]$

$$\begin{aligned}\bigotimes_{k=1}^{t}((\mathcal{E}_p)_{A_j^{(k)}} \otimes \mathsf{id}_{B_j^{(k)}})(|\Phi\rangle\langle\Phi|_{A_j^{(k)} B_{j+1}^{(k)}}) &= \sum_{x_1,\ldots,x_t \in \{0,1\}} \bigotimes_{k=1}^{t} p_{x_k} (\mathcal{E}_1^{x_k} \otimes \mathsf{id})(|\Phi\rangle\langle\Phi|_{A_j^{(k)} B_{j+1}^{(k)}}) \\ &= \left(p^t\left((\mathcal{E}_1 \otimes \mathsf{id})(|\Phi\rangle\langle\Phi|)\right)^{\otimes t} + \left(1 - p^t\right)\sigma\right)_{A_j B_{j+1}}\ ,\end{aligned}$$

where we collected all terms with $(x_1, \ldots, x_t) \neq 1^t$ in a state $\sigma_{A_j B_{j+1}}$. Because $\mathcal{E}_1$ is entanglement-breaking, the state $\rho_{A_j : B_{j+1}} := ((\mathcal{E}_1 \otimes \mathsf{id})(|\Phi\rangle\langle\Phi|))^{\otimes t}$ is separable across $A_j : B_{j+1}$. We have shown that

$$\begin{aligned}\rho^{\text{initial, noisy}}_{S_1 \cdots S_R} &= \left(\bigotimes_{j=1}^{R-1}((1-q)\sigma_{A_j B_{j+1}} + q\rho_{A_j : B_{j+1}})\right) \otimes \left(\bigotimes_{j=1}^{R} \rho_{C_j}\right) \\ &= (1-q)^{R-1}\left(\bigotimes_{j=1}^{R-1} \sigma_{A_j B_{j+1}}\right) \otimes \left(\bigotimes_{j=1}^{R} \rho_{C_j}\right) + \Sigma_{\text{SEP}}\end{aligned}$$

where

$$\Sigma_{\text{SEP}} := \sum_{(z_1,\ldots,z_{R-1}) \in \{0,1\}^{R-1} \setminus \{0^{R-1}\}} \bigotimes_{j=1}^{R-1} (1-q)^{1-z_j} q^{z_j} \sigma^{1-z_j}_{A_j B_{j+1}} \rho^{z_j}_{A_j : B_{j+1}} \otimes \left(\bigotimes_{j=1}^{R} \rho_{C_j}\right)$$

and $q := p^t$. Since every summand in Σ_{SEP} is separable with respect to the bipartition $S_1 \cdots S_j : S_{j+1} \cdots S_R$, the claim (95) follows. □

Theorem 6.2 now follows by applying the reduction from a general protocol π to an entanglement-based protocol π'.

Proof of Theorem 6.2. Suppose π is a depth-Δ-circuit which generates distance-R-entanglement between two qubits using m qubits per site. Without loss of generality (see the remark above), assume that all two-qubit gates between sites are CNOT gates. Let t be the maximal number of nearest-neighbor CNOT gates used between two neighboring sites. Then $t \leq \Delta m$.

Let π' be the entanglement-based protocol which uses the resource state $\Phi^{\otimes t}$. Then, according to Lemma 6.3, there is a local stochastic error $E \sim \mathcal{N}(p^2)$ acting on each half of the Bell states in the initial state (93) such that π' generates the final state (94) with

$$p_{\text{fail}} \geq 1 - e^{-R(4p^2/3)^t} \geq 1 - e^{-R(4p^2/3)^{\Delta m}}\ .$$

By property (iii) of the entanglement-based protocol π', the local stochastic error $E \sim \mathcal{N}(p^2)$ on the resource state of π' is equivalent to (a certain) local stochastic noise of strength $\mathcal{N}(p)$ in the execution of π. The claim follows from this. □

6.2 A converse bound on cluster-state based schemes

Corollary 6.1 shows that any fault-tolerant low-latency scheme for entanglement generation requires

$$m \geq \Omega(\log R) \tag{96}$$

qubits per repeater. For our cluster-state based scheme, the number m of qubits per repeater is given by $m = \Theta(d^2)$, and our achievability result requires $d = \Omega(\log R)$. Thus our scheme works with

$$m = \Theta(\log^2 R) \tag{97}$$

qubits per site.

Comparing (96) with (97), we observe a gap between our general no-go result and the achievability statement of Corollary 6.1. In particular, (96) leaves room for an improved achievability result, either by a better analysis of our scheme or by considering different schemes.

In this section, we show that, in fact, the scaling (97) resulting for the cluster-state based scheme is optimal, i.e., our analysis is tight. We also find that a better scaling cannot be obtained by modifying the classical decoding functions only: Any scheme which uses the cluster state $W\left|0^{\mathcal{C}}\right\rangle$ with the lattice $\mathcal{C}[d \times d \times R]$, applies the same measurement pattern and relies on the same syndrome information (associated with observed boundaries on the decoding graph) requires at least $m = \Omega(\log^2 R)$ qubits per repeater (i.e., within any plane with u_3 fixed).

Theorem 6.4 (Converse bound for cluster-state based schemes)**.** *Let $0 < p \leq \frac{1}{4}$. Consider an (infinite) family of pairs (d, R) such that d is even and R satisfies*

$$R \geq \left(\frac{1}{2\sqrt{p}}\right)^d . \tag{98}$$

Suppose we run Algorithm 2 on the input state $EW\left|0^{\mathcal{C}}\right\rangle$ in the cluster lattice $\mathcal{C}[d \times d \times R]$ which is corrupted by i.i.d. single-qubit bit-flip noise with parameter p, i.e., $E = \prod_{c\in\mathcal{C}} X_c^{y_c}$ where $\{y_c\}_{c\in\mathcal{C}}$ are independent and identically distributed Bernoulli-p random variables, i.e., $\Pr[y_c = 1] = p$. Then the probability $\nu_{(0,0)}$ of successful entanglement generation satisfies

$$\nu_{(0,0)} \leq 3/4 \qquad \textit{for all sufficiently large } d .$$

The same statement applies to any scheme obtained from Algorithm on 2 by replacing the function $\mathsf{MinMatch}$ *by an (arbitrary) binary function.*

Proof. We first argue that the conclusions of this Theorem hold for our protocol. Recall the definition (55) of the operator E_{gl} for any Pauli operator E on $\mathcal{C}$. For the bit-flip noise model we consider here, there are no Pauli-Z errors (i.e., $E^Z = 0$), and thus

$$E_{\mathrm{gl}} = E^X|_{\mathsf{E}_{\mathrm{cl,dec}}\cap\mathcal{A}} = \prod_{c\in\mathsf{E}_{\mathrm{cl,dec}}\cap\mathcal{A}} X_c^{y_c} .$$

It follows that the two random variables A_Z and B_Z defined in (83) are given by

$$A_Z = 0 \tag{99}$$
$$B_Z = \left\langle\!\left\langle \mathrm{supp}(E^X|_{\mathsf{E}_{\mathrm{cl,dec}}\cap\mathcal{A}}) \oplus \mathsf{MinMatch}_{\mathsf{T}_{\mathrm{cl,dec}}}(\partial_{\mathsf{T}_{\mathrm{cl,dec}}}\mathrm{supp}(E^X|_{\mathsf{E}_{\mathrm{cl,dec}}\cap\mathcal{A}})), \mathcal{L}_{\mathrm{cl},X} \right\rangle\!\right\rangle . \tag{100}$$

Eq. (84) implies that

$$\begin{aligned} 1 - \nu_{(0,0)} &\geq \Pr\left[(A_Z, B_Z) = (0,1)\right] \\ &= \Pr\left[B_Z = 1\right] , \end{aligned}$$

where we used (99), i.e., the fact that $A_Z = 0$ with probability 1. Our claim thus follows if we establish the lower bound

$$\Pr[B_Z = 1] \geq 1/4 \; . \tag{101}$$

Because of (100) and the definition of our noise model, we may express the probability of interest as

$$\Pr[B_Z = 1] = \Pr_Y \left[Y \in \mathcal{E}^{(1)} \right]$$

where

$$\mathcal{E}^{(b)} := \left\{ y \subseteq \mathsf{E}_{\mathrm{cl,dec}} \mid \left\langle\!\left\langle (y \cap \mathcal{A}) \oplus \mathsf{MinMatch}_{\mathsf{T}_{\mathrm{cl,dec}}}(\partial_{\mathsf{T}_{\mathrm{cl,dec}}}(y \cap \mathcal{A})), \mathcal{L}_{\mathrm{cl},X} \right\rangle\!\right\rangle = b \right\} \qquad \text{for } b = 0, 1 \tag{102}$$

(We will need $\mathcal{E}^{(0)}$ below.) Here $Y = \{Y_e\}_{e \in \mathsf{E}_{\mathrm{cl,dec}}}$ are i.i.d. Bernoulli random variables with parameter p. This is because we have $Y = \mathrm{supp}(E^X|_{\mathsf{E}_{\mathrm{cl,dec}} \cap \mathcal{A}})$ by definition of the noise model.

We will prove (101) by showing that there is a subset $\mathcal{E}' \subset \mathcal{E}$ that has probability at least

$$\Pr_Y [Y \in \mathcal{E}'] \geq 1/4 \tag{103}$$

under this distribution.

To construct $\mathcal{E}'$ let us call a subset $y \subset \mathsf{E}_{\mathrm{cl,dec}}$ appropriate if it contains exactly $d/2$ edges of any length-d, i.e., shortest-length path connecting the left and the right faces. We denote by $\mathcal{E}_{\mathsf{appr}}$ the set of appropriate subsets of $\mathsf{E}_{\mathrm{cl,dec}}$, and define

$$\mathcal{E}' := \mathcal{E}^{(1)} \cap \mathcal{E}_{\mathsf{appr}} \subset \mathcal{E}^{(1)}$$

as the set of all $y \in \mathcal{E}$ that are appropriate. To state this formally, let e_1 be the first canonical basis vector in $\mathbb{R}^3$. For any $0 \leq j \leq d-1$ and $1 \leq k \leq \frac{R-1}{2}$, consider the two external vertices u_j^k and v_j^k of $\mathsf{T}_{\mathrm{cl,dec}}$ defined in (68) and (69). We then consider the path $P_{j,k} \subset \mathsf{E}_{\mathrm{cl,dec}}$ obtained by traversing sequence of vertices

$$u_j^k, u_j^k + 2e_1, u_j^k + 4e_1, \ldots, u_j^k + 2(d-1)e_1, u_j^k + 2de_1 = v_j^k \; .$$

The path $P_{j,k}$ is the shortest path (of length d) connecting two endpoints u_j^k and v_j^k. A subset $y \subset \mathsf{E}_{\mathrm{cl,dec}}$ is then called appropriate (i.e., $y \in \mathcal{E}_{\mathsf{appr}}$) if there are (j,k) with $0 \leq j \leq d-1$ and $1 \leq k \leq \frac{R-1}{2}$ such that

$$|y \cap P_{j,k}| = d/2 \; . \tag{104}$$

It remains to find a lower bound on

$$\Pr_Y \left[Y \in \mathcal{E}^{(1)} \cap \mathcal{E}_{\mathsf{appr}} \right] \; . \tag{105}$$

In order to do so, we use the following observation.

Lemma 6.5. *Consider the sets* $\mathcal{E}^{(0)}, \mathcal{E}^{(1)}$ *(cf.* (102)*) and* $\mathcal{E}_{\mathsf{appr}}$. *Let* $b \in \{0,1\}$ *be arbitrary. Then the following holds.*

(i) For every $y \in \mathcal{E}^{(b)} \cap \mathcal{E}_{\mathsf{appr}}$, *there is an element* $\widehat{y} \in \mathcal{E}^{(b \oplus 1)} \cap \mathcal{E}_{\mathsf{appr}}$ *such that* $|y| = |\widehat{y}|$, *i.e.,* y *and* $\widehat{y}$ *have the same Hamming weight.*

(ii) The map $y \mapsto \widehat{y}$ *is injective.*

Proof. We give the proof for $b = 0$ (the case $b = 1$ is analogous). That is, consider $y \in \mathcal{E}^{(0)} \cap \mathcal{E}_{\mathsf{appr}}$. Because y is appropriate, there is a – not necessarily unique – path $P_{j,k}$ such that (104) holds. In the following, we impose uniqueness by lexicographically ordering the set of pairs (j,k) with $0 \le j \le d-1$ and $1 \le k \le \frac{R-1}{2}$, and using the first (lowest) such pair (j,k) with property (104). We then define

$$\widehat{y} := y \oplus P_{j,k}$$

as the symmetric difference of y and $P_{j,k}$. It is easy to check that the map $y \mapsto \widehat{y}$ is well-defined.

Because $|P_{j,k}| = d$, Eq. (104) and the definition of $\widehat{y}$ imply that $|\widehat{y}| = d/2$. In particular, we have $\widehat{y} \in \mathcal{E}_{\mathsf{appr}}$. With this property and the fact that the paths $\{P_{j,k}\}_{j,k}$ are pairwise disjoint, it is easy to see that the map $y \mapsto \widehat{y}$ has a left-inverse (obtained by applying the same construction again). Hence it is injective.

It remains to show that $\widehat{y} \in \mathcal{E}^{(1)}$ for $y \in \mathcal{E}^{(0)} \cap \mathcal{E}_{\mathsf{appr}}$. To this end, observe first that

$$\partial_{\mathsf{T}_{\mathrm{cl,dec}}}(\widehat{y} \cap \mathcal{A}) = \partial_{\mathsf{T}_{\mathrm{cl,dec}}}(y \cap \mathcal{A}) \ . \tag{106}$$

Eq. (106) follows from the linearity of the boundary operator and the fact that the boundary of path is given by its endpoints. In our case, the endpoints of $P_{j,k}$ are the external vertices $\{u_j^k, v_j^k\}$ of $\mathsf{T}_{\mathrm{cl,dec}}$, but these do not contribute to the boundary $\partial_{\mathsf{T}_{\mathrm{cl,dec}}} P_{j,k}$ by definition of $\partial_{\mathsf{T}_{\mathrm{cl,dec}}}$. This establishes (106).

We also use the following property of the path $P_{j,k}$: We have

$$\langle\!\langle P_{j,k} \cap \mathcal{A}, \mathcal{L}_{\mathrm{cl},X} \rangle\!\rangle = 1 \tag{107}$$

since the subset $\mathcal{L}_{\mathrm{cl},X}$ lies in a plane perpendicular to $P_{j,k}$ with a single intersecting edge. Combining (106) and (107), and using linearity, we have

$$\begin{aligned}
\Big\langle\!\!\Big\langle (\widehat{y} \cap \mathcal{A}) \oplus \mathsf{MinMatch}_{\mathsf{T}_{\mathrm{cl,dec}}}(\partial_{\mathsf{T}_{\mathrm{cl,dec}}}(\widehat{y} \cap \mathcal{A})), \mathcal{L}_{\mathrm{cl},X} \Big\rangle\!\!\Big\rangle &= \Big\langle\!\!\Big\langle (y \cap \mathcal{A}) \oplus \mathsf{MinMatch}_{\mathsf{T}_{\mathrm{cl,dec}}}(\partial_{\mathsf{T}_{\mathrm{cl,dec}}}(y \cap \mathcal{A})), \mathcal{L}_{\mathrm{cl},X} \Big\rangle\!\!\Big\rangle \oplus 1 \\
&= 0 \oplus 1 \\
&= 1
\end{aligned}$$

where we used the assumption that $y \in \mathcal{E}^{(0)}$ in the penultimate step. This shows that $\widehat{y} \in \mathcal{E}^{(1)}$ as claimed. $\square$

Returning to the problem of bounding (105), we observe that

$$\begin{aligned}
\Pr_Y\left[Y \in \mathcal{E}^{(1)} \cap \mathcal{E}_{\mathsf{appr}}\right] &= \sum_{y \in \mathcal{E}^{(1)} \cap \mathcal{E}_{\mathsf{appr}}} P_Y(y) \\
&= \sum_{y \in \mathcal{E}^{(1)} \cap \mathcal{E}_{\mathsf{appr}}} P_Y(\widehat{y}) \\
&\le \sum_{y \in \mathcal{E}^{(0)} \cap \mathcal{E}_{\mathsf{appr}}} P_Y(y) \\
&= \Pr_Y\left[Y \in \mathcal{E}^{(0)} \cap \mathcal{E}_{\mathsf{appr}}\right] \ ,
\end{aligned}$$

where we used that $P_Y(y)$ only depends on the Hamming weight $|y|$ of y, and $|\widehat{y}| = |y|$. We also used that $\widehat{y} \in \mathcal{E}^{(0)} \cap \mathcal{E}_{\mathsf{appr}}$ and the fact that the map $y \mapsto \widehat{y}$ is injective to obtain the inequality. By symmetry, i.e., interchanging 0 and 1, we also have the converse inequality and thus the identity

$$\Pr_Y\left[Y \in \mathcal{E}^{(0)} \cap \mathcal{E}_{\mathsf{appr}}\right] = \Pr_Y\left[Y \in \mathcal{E}^{(1)} \cap \mathcal{E}_{\mathsf{appr}}\right] \ .$$

(In fact, it is possible to construct a Hamming-weight-preserving bijection between the two sets $\mathcal{E}^{(b)} \cap \mathcal{E}_{\mathsf{appr}}$, $b = 0, 1$, but we do not need this here.)

Because the sets $\mathcal{E}^{(0)}, \mathcal{E}^{(1)}$ are disjoint, and the union is the set of all subsets of $\mathsf{E}_{\text{cl,dec}}$, we conclude that

$$\Pr_Y\left[Y \in \mathcal{E}^{(1)} \cap \mathcal{E}_{\mathsf{appr}}\right] = \frac{1}{2}\left(\Pr_Y\left[Y \in \mathcal{E}^{(0)} \cap \mathcal{E}_{\mathsf{appr}}\right] + \Pr_Y\left[Y \in \mathcal{E}^{(1)} \cap \mathcal{E}_{\mathsf{appr}}\right]\right)$$
$$= \frac{1}{2}\Pr_Y\left[Y \in \mathcal{E}_{\mathsf{appr}}\right] \ . \tag{108}$$

We have

$$\Pr_Y\left[Y \in \mathcal{E}_{\mathsf{appr}}\right] = 1 - \Pr_Y\left[Y \notin \mathcal{E}_{\mathsf{appr}}\right]$$
$$= 1 - \Pr_Y\left[|Y \cap P_{j,k}| \neq \frac{d}{2} \text{ for all } (j,k)\right]$$
$$= 1 - \left(1 - \binom{d}{d/2}(2p)^{d/2}(1-2p)^{d/2}\right)^{d \cdot \frac{R-1}{2}} \ . \tag{109}$$

In the last step, we used the fact that Y consists of independent random Bernoulli variables, and the fact that the paths $\{P_{j,k}\}_{j,k}$ are pairwise disjoint.

By Stirling's formula $\binom{2k}{k} = (1+o(1))\frac{2^{2k}}{\sqrt{\pi k}}$ for $k \to \infty$ and the inequality $2p(1-2p) \geq p$ for $p \leq \frac{1}{4}$ we obtain

$$\binom{d}{d/2}(2p)^{d/2}(1-2p)^{d/2} \geq (1+o(1))\frac{2^d}{\sqrt{\pi d/2}}p^{d/2}$$
$$\geq 0.3\frac{(2\sqrt{p})^d}{\sqrt{d}} \qquad \text{for} \qquad d \text{ sufficiently large} \ .$$

We have

$$(1-x)^r \leq e^{-rx} \qquad \text{for all} \qquad x \in (0,1) \qquad \text{and} \qquad r \in \mathbb{N} \ ,$$

hence

$$\left(1 - \binom{d}{d/2}(2p)^{d/2}(1-2p)^{d/2}\right)^{d \cdot \frac{R-1}{2}} \leq \exp\left(-0.15\sqrt{d} \cdot (R-1) \cdot (2\sqrt{p})^d\right) \ .$$

We conclude with (109) and (108) that

$$\Pr_Y\left[Y \in \mathcal{E}^{(1)} \cap \mathcal{E}_{\mathsf{appr}}\right] \geq \frac{1}{2}\left(1 - \exp\left(-0.15\sqrt{d} \cdot (R-1) \cdot (2\sqrt{p})^d\right)\right)$$
$$\geq 1/4$$

for sufficiently large d if

$$\lim_{d \to \infty} \sqrt{d} \cdot (R-1) \cdot (2\sqrt{p})^d = \infty \ . \tag{110}$$

Condition (110) is satisfied when (98) holds. In particular, we obtain the claim (103) for sufficiently large d.

It remains to show that this argument also applies to protocols where the matching function $\mathsf{MinMatch}$ is replaced by a different function in Algorithm 2. This is because – as discussed at the end of Section 4 – Theorem 4.6 does not require $\mathsf{MinMatch}$ to return a matching of a given input. Neither does (84), which is obtained from Theorem 4.6.

□

A Products of stabilizer generators of the graph state

In this appendix, we give proofs of Lemma 4.1 and Lemma 4.2 concerning products of stabilizer generators of the cluster state.

Proof of Lemma 4.1. Let (e_1, e_2, e_3) be the standard basis of $\mathbb{R}^3$.

(i) Consider an element $u = (u_1, u_2, u_3) \in \mathsf{V}^{\mathrm{int}}_{\mathrm{cl,dec}}$ with $u_3 \notin \{1, R\}$, i.e., $u \in \mathsf{V}_{\mathrm{even}}$ with $\mathrm{neigh}(u) \subset \mathsf{E}_{\mathrm{even}}$.

By definition, any $v \in \mathrm{neigh}(u)$ has the form

$$v = u + s_j e_j \qquad \text{with} \qquad j \in \{1, 2, 3\} \qquad \text{and} \qquad s_j \in \{-1, 1\} \ . \tag{111}$$

Moreover, for any such $v = u + s_j e_j \in \mathrm{neigh}(u)$, any $w \in \mathrm{neigh}(v)$ takes the form

$$w = u + s_j e_j + s_k e_k \qquad \text{with} \qquad k \in \{1, 2, 3\} \ , k \neq j \qquad \text{and} \qquad s_k \in \{-1, 1\} \ . \tag{112}$$

This is because $\mathrm{neigh}(v) \subset \mathcal{C}$ does not contain elements of the form (e, e, e), and thus $u \notin \mathrm{neigh}(v)$. It follows from the definition (47) of S^u for $u \in \mathsf{V}^{\mathrm{int}}_{\mathrm{cl,dec}}$ with $u_3 \notin \{1, R\}$ that

$$\begin{aligned} S^u &= \prod_{v \in \mathrm{neigh}(u)} \left(Z_v \prod_{w \in \mathrm{neigh}(v)} X_w \right) & (113) \\ &= \left(\prod_{v \in \mathrm{neigh}(u)} Z_v \right) \Bigg(\prod_{\substack{(v,w): \\ v \in \mathrm{neigh}(u) \\ w \in \mathrm{neigh}(v)}} X_w \Bigg) \ . & (114) \end{aligned}$$

Here we used that for any two distinct $v, v' \in \mathrm{neigh}(u)$, we have $v \notin \mathrm{neigh}(v')$ (as follows immediately from (111)), implying that the product (113) does not contain a factor X_v.

Now suppose that v, w are such that $v \in \mathrm{neigh}(u)$ and $w \in \mathrm{neigh}(v)$. Assume that v and w are of the form (111) and (112), respectively, i.e.,

$$\begin{aligned} v &= u + s_j e_j \\ w &= u + s_j + s_k e_k \ . \end{aligned}$$

Consider

$$v' := u + s_k e_k \ .$$

Then it follows from $u \in \widetilde{\mathcal{C}}$ and $v, w \in \mathcal{C}$ that $v' \in \mathcal{C}$. In particular, we have

$$\begin{aligned} &v' \in \mathrm{neigh}(u) \\ &w \in \mathrm{neigh}(v') \ . \end{aligned}$$

This shows that associated with every pair (v, w) giving rise to a factor X_w in the product (114), there is a pair $(v', w) \neq (v, w)$ also contributing a factor X_w. Thus all such factors cancel and we are left with

$$S^u = \prod_{v \in \mathrm{neigh}(u)} Z_v = \prod_{v \in \mathrm{Inci}_{\mathsf{T}_{\mathrm{cl,dec}}}(u)} Z_v \tag{115}$$

because $\mathrm{neigh}(u) = \mathrm{Inci}_{\mathsf{T}_{\mathrm{cl,dec}}}(u)$ by definition of $\mathsf{T}_{\mathrm{cl,dec}}$. In particular, we have $\mathrm{supp}(S^u) = \mathrm{Inci}_{\mathsf{T}_{\mathrm{cl,dec}}}(u)$ as claimed. Let us argue that for $u \in \mathsf{V}^{\mathrm{int}}_{\mathrm{cl,dec}}$ with $u_3 \notin \{1, R\}$ we have

$$\mathrm{neigh}(u) = \mathrm{Inci}_{\mathsf{T}_{\mathrm{cl,dec}}}(u) \subset \mathcal{A} \tag{116}$$

(and thus in particular $\mathrm{Inci}_{\mathsf{T}_{\mathrm{cl,dec}}}(u) \cap \mathcal{B} = \emptyset$). Observe that combining (115) with (116) implies the claim (48) for $u \in \mathsf{V}^{\mathrm{int}}_{\mathrm{cl,dec}}$ with $u_3 \notin \{1, R\}$. To show (116), consider $v \in \mathrm{neigh}(u)$ of the form $v = u + s_j e_j$. Suppose that $v_3 \in \{1, R\}$. Then this implies that either

$$u_3 = 2, j = 3, s_j = -1 \qquad \text{or} \qquad u_3 = R-1, j = 3, s_j = 1 \ .$$

In both cases, it follows that $v \in \{(e, e, o)\}$ (since $u \in \{(e, e, e)\}$) and thus $v \in \mathcal{A}$. This implies (116) since any $v \in \mathrm{neigh}(u)$ with $v_3 \notin \{1, R\}$ satisfies $v \in \mathcal{A}$.

(ii) Consider an element $u \in \mathsf{V}^{\mathrm{int}}_{\mathrm{cl,dec}}$ with $u_3 \in \{1, R\}$. We claim that

$$\begin{aligned} \{u\} &= \mathrm{Inci}_{\mathsf{T}_{\mathrm{cl,dec}}}(u) \cap \mathcal{A} \\ \mathrm{neigh}(u) &= \mathrm{Inci}_{\mathsf{T}_{\mathrm{cl,dec}}}(u) \cap \mathcal{B} \ . \end{aligned} \tag{117}$$

Note that the claim (48) follows from (117) combined with the definition of G_u. Observe that u is incident to an edge

$$\{u, v\} \qquad \text{where} \qquad v = \begin{cases} (u_1, u_2, 2) & \text{if } u_3 = 1 \\ (u_1, u_2, R-1) & \text{if } u_3 = R \ . \end{cases} \tag{118}$$

To describe the edges in the subgraph $\mathsf{T}_{\mathrm{dec}} \times \{u_3\}$, let us define

$$\begin{aligned} \widetilde{\mathcal{C}}_{u_3} &= \{w \in \widetilde{\mathcal{C}} \mid w_3 = u_3\} \\ \mathcal{C}_{u_3} &= \{w \in \mathcal{C} \mid w_3 = u_3\} \ . \end{aligned}$$

Then $\widetilde{\mathcal{C}}_{u_3}$ and $\mathcal{C}_{u_3}$ are in one-to-one correspondence with the set of two-dimensional sites and the set of locations of qubits introduced in Section 1.1, respectively. For a site $w = (w_1, w_2, u_3) \in \widetilde{\mathcal{C}}_{u_3}$, we denote by $\mathrm{neigh}_{u_3}(w)$ the set of nearest neighbors of w in $\mathcal{C}_{u_3}$. More explicitly,

$$\mathrm{neigh}_{u_3}(w) = \left\{ z \in \mathcal{C}_{u_3} \mid \sum_{j=1}^{2} |w_j - z_j| = 1 \right\} \ .$$

From the definition (46) of $\mathsf{V}^{\mathrm{int}}_{\mathrm{dec}}$, it follows that $(u_1, u_2) \in \mathsf{V}^{\mathrm{int}}_{\mathrm{dec}}$. Then it follows from the definition (3) that

$$\mathrm{Inci}_{\mathsf{T}_{\mathrm{dec}} \times \{u_3\}}(u) = \mathrm{neigh}_{u_3}(u) \ ,$$

where each sites in $\mathrm{neigh}_{u_3}(u)$ is the midpoint of an edge of the lattice $\mathsf{T}_{\mathrm{dec}} \times \{u_3\}$. Furthermore, it is easy to see that

$$\mathrm{neigh}_{u_3}(u) = \mathrm{neigh}(u) \ .$$

By construction of $\mathsf{T}_{\mathrm{cl,dec}}$, the edge $\{u, v\}$ from (118) and the edges in $\mathrm{Inci}_{\mathsf{T}_{\mathrm{dec}} \times \{u_3\}}$ form the complete set of the edges where u is incident in $\mathsf{T}_{\mathrm{cl,dec}}$. That is,

$$\mathrm{Inci}_{\mathsf{T}_{\mathrm{cl,dec}}}(u) = \mathrm{neigh}(u) \cup \{u\}. \tag{119}$$

The claim (117) follows from (119) and the fact that $u \in \mathcal{A}$ and $\mathrm{neigh}(u) \subset \mathcal{B}$.

The cases for the dual decoding graph can be checked in analogous manner. □

Proof of Lemma 4.2. We show the claim (51). Let $u = (u_1, u_2, u_3) \in \mathcal{L}_{\mathrm{cl},X} \cap \mathcal{B}$. Then $u_1 = 1$, and u_2, u_3 are even. The stabilizer generator G_u is

$$G_u = \begin{cases} X_{(1,u_2,u_3-1)} Z_{(1,u_2,u_3)} X_{(1,u_2+1,u_3)} X_{(1,u_2,u_3+1)} & \text{if } u_2 = 0 \\ X_{(1,u_2,u_3-1)} Z_{(1,u_2,u_3)} X_{(1,u_2+1,u_3)} X_{(1,u_2-1,u_3)} X_{(u_1,u_2,u_3+1)} & \text{if } 2 \le u_2 \le 2d-4 \\ X_{(1,u_2,u_3-1)} Z_{(1,u_2,u_3)} X_{(1,u_2-1,u_3)} X_{(1,u_2,u_3+1)} & \text{if } u_2 = 2d-2 \ . \end{cases}$$

Define the operators

$$\mathcal{G}_j := \begin{cases} \prod_{k=2,4,\ldots,R-1} Z_{(1,0,k)}X_{(1,1,k)} & \text{if } j=0 \\ \prod_{k=2,4,\ldots,R-1} Z_{(1,j,k)}X_{(1,j-1,k)}X_{(1,j+1,k)} & \text{if } j=2,4,\ldots,2d-4 \\ \prod_{k=2,4,\ldots,R-1} Z_{(1,r-1,k)}X_{(1,r-2,k)} & \text{if } j=2d-2 \ . \end{cases} \tag{120}$$

Then it is straightforward to check that

$$\prod_{k=2,4,\ldots,R-1} G_{(1,j,k)} = X_{(1,j,1)}\mathcal{G}_j X_{(1,j,R)} \qquad \text{for} \qquad j=0,2,\ldots,2d-2 \ .$$

We observe that for all even j,j' and $u_3 \in \{1,R\}$, the site $(1,j,u_3)$ does not appear in any of the factors in the product $\mathcal{G}_{j'}$ in (120). This implies that $X_{(1,j,u_3)}$ and $\mathcal{G}_{j'}$ commute, and thus

$$\begin{aligned} S^X &= \prod_{u\in\mathcal{L}_{\mathrm{cl},X}\cap\mathcal{X}} G_u \\ &= \prod_{j=0,2,\ldots,2d-2}\left(\prod_{k=2,4,\ldots,R-1} G_{(1,j,k)}\right) \\ &= \prod_{j=0,2,\ldots,2d-2} X_{(1,j,1)}\mathcal{G}_j X_{(1,j,R)} \\ &= \left(\prod_{j=0,2,\ldots,2d-2} X_{(1,j,1)}X_{(1,j,R)}\right)\left(\prod_{j=0,2,\ldots,2d-2}\mathcal{G}_j\right) \\ &= X_{q_1}X_{q_2}X(\mathcal{L}_{\mathrm{cl},X}\cap\mathcal{B})\left(\prod_{j=0,2,\ldots,2d-2}\mathcal{G}_j\right) \ . \end{aligned} \tag{121}$$

It is easy to check that in $\prod_{j=0,2,\ldots,2d-2}\mathcal{G}_j$ all single-qubit Pauli operators commute and that all Pauli-X are cancelled. As a result, we have

$$\prod_{j=0,2,\ldots,2d-2}\mathcal{G}_j = \prod_{\substack{j=0,2,\ldots,2d-2\\ k=2,4,\ldots,R-1}} Z_{(1,j,k)} = Z(\mathcal{L}_{\mathrm{cl},X}\cap\mathcal{A}) \ . \tag{122}$$

The claim (51) follows from (121) and (122). The claim (52) can be checked in analogous manner. □

B An upper bound on the resilience function $\mathsf{res}_{\mathcal{L}_Z}(p)$

Proof of Lemma 3.6 . We label the external vertices of $\mathsf{T}_{\mathrm{dec}^*}$ as

$$\mathsf{V}^{\mathrm{ext}}_{\mathrm{dec}^*} = \{u_1,\ldots,u_d,v_1,\ldots,v_{d-1}\} \qquad \text{with} \qquad u_j=(2j-1,2j-1) \text{ and } v_k=(2k+1,-1) \ ,$$

see Fig. 3b Clearly, a simple path $P\in Z_{\mathrm{ext}}(\mathsf{T}_{\mathrm{dec}^*})$ with external endpoints through internal vertices satisfies $\langle\!\langle P,\mathcal{L}_Z\rangle\!\rangle = 1$ if and only if it starts at a vertex u_j (with $j\in\{1,\ldots,d\}$) and ends at a vertex v_k (with $k\in\{1,\ldots,d-1\}$) without visiting any other external vertex. For $j\in\{1,\ldots,d\}$ and $\ell\in\mathbb{N}$, let $\Delta(u_j,\ell)$ be the set of all such paths $P\in Z_{\mathrm{ext}}(\mathsf{T}_{\mathrm{dec}^*})$ that start at u_j and end at some vertex in the set $\{v_k\}_{k=1}^{d-1}$. By the same argument as used for showing (34), we have

$$\mathsf{res}_{\mathcal{L}_Z}(p) = \sum_{j=1}^{d}\sum_{\ell=L_j^{\min}}^{L_j^{\max}} \binom{\ell}{\lceil\ell/2\rceil}\cdot|\Delta(u_j,\ell)|\cdot p^{\lceil\ell/2\rceil} \ . \tag{123}$$

Here $L_j^{\min}$ and $L_j^{\max}$ are the minimal and maximal lengths of a path $P\in Z_{\mathrm{ext}}(\mathsf{T}_{\mathrm{dec}^*})$ starting at u_j and ending in $\{v_k\}_{k=1}^{d-1}$.

The set $\Delta(u_1,1)$ is empty and the set $\Delta(u_1,2)$ consists of a single path, i.e., $|\Delta(u_1,1)|=0$ and $|\Delta(u_1,2)|=1$. For $j\in\{2,\ldots,d-1\}$, we use that the graph $\mathsf{T}_{\mathrm{dec}^*}$ has vertices of degree at most 4, which implies that

$$|\Delta(u_j,\ell)|\le 3^{\ell-1}\qquad\text{for all}\qquad \ell\in\mathbb{N}$$

as in the proof of Lemma 3.5. Again using

$$L_j^{\min}\ge j\ .$$

and the inequalities $p^{\lceil \ell/2\rceil}\le p^{\ell/2}$ and $\binom{\ell}{\lceil \ell/2\rceil}\le 2^\ell$ for $\ell>1$ in the expression (123), we have that

$$\begin{aligned}\mathsf{res}_{\mathcal{L}_Z^*} &\le \binom{2}{1}1\cdot p+\sum_{\ell=3}^{L_1^{\max}}2^\ell 3^{\ell-1}p^{\ell/2}+\sum_{j=2}^{d}\sum_{\ell=j}^{L_j^{\max}}2^\ell 3^{\ell-1}p^{\ell/2}\\ &\le 2p+\frac{1}{3}\sum_{\ell=3}^{\infty}q^\ell+\frac{1}{3}\sum_{j=2}^{d}\sum_{\ell=j}^{\infty}q^\ell \qquad\text{where}\qquad q:=6\sqrt{p}\\ &=2p+\frac{q^3}{3(1-q)}+\frac{q^2-q^{d-1}}{3(1-q)^2}.\end{aligned}$$

Again, q satisfies $q\le 6\sqrt{\frac{1}{144}}\le\frac{1}{2}$. For such q, the inequalities $\frac{q}{(1-q)}\le 1$ and $\frac{1}{(1-q)^2}\le 4$ hold and we conclude that

$$\begin{aligned}\mathsf{res}_{\mathcal{L}_Z^*}&\le 2p+\frac{1}{3}\cdot(6\sqrt{p})^2+\frac{4}{3}\cdot(6\sqrt{p})^2\\ &=38p\ .\end{aligned}$$

□

C An upper bound on the resilience function $\mathsf{res}_{\mathcal{L}^*_{\mathrm{cl},Z}}(p)$

In this appendix, we derive an upper bound on the resilience function $\mathsf{res}_{\mathcal{L}^*_{\mathrm{cl},Z}}(p)$. That is, we give the

Proof of Lemma 5.2. The proof is analogous to that of Lemma 5.1. As before, it is sufficient to consider the set $Z_{\mathrm{ext}}(\mathsf{T}_{\mathrm{cl,dec}^*})$ in order to compute the resilience $\mathsf{res}_{\mathcal{L}^*_{\mathrm{cl},Z}}(p)$. We partition the set of external vertices

$$\mathsf{V}^{\mathrm{ext}}_{\mathrm{cl,dec}^*}=\mathsf{V}^{\mathrm{ext,bottom}}_{\mathrm{cl,dec}^*}\cup\mathsf{V}^{\mathrm{ext,top}}_{\mathrm{cl,dec}^*}$$

into the set $\mathsf{V}^{\mathrm{ext,bottom}}_{\mathrm{cl,dec}^*}$ of vertices (u_1,u_2,u_3) forming the "bottom" boundary with $u_2=-1$ and the set $\mathsf{V}^{\mathrm{ext,top}}_{\mathrm{cl,dec}^*}$ remaining vertices which form the boundaries on the "top" ($u_2=r$), "front" ($u_1=1$) and "back" ($u_1=R$). We label the external vertices as

$$\begin{aligned}\mathsf{V}^{\mathrm{ext,bottom}}_{\mathrm{cl,dec}^*}&=\left\{v_i^k\mid 1\le i\le d-1,k\in\{1,D\}\right\}\cup\left\{v_i^k\mid 0\le i\le d-1,2\le k\le\frac{R-1}{2}\right\}\\ \mathsf{V}^{\mathrm{ext,top}}_{\mathrm{cl,dec}^*}&=\left\{u_{i,j}^k\mid 1\le i\le j,1\le j\le d-1,k\in\{1,\frac{R-1}{2}\}\right\}\\ &\cup\left\{u_i^k\mid 1\le i\le d,2\le k\le\frac{R-1}{2}\right\}\cup\left\{u_d^1,u_d^{\frac{R-1}{2}}\right\}\ ,\end{aligned}$$

see Fig. 6. Written out, the external vertices are located at the following (dual) sites:

$$\begin{aligned}v_i^k&=(2i+1,-1,2k-1)\\ u_{i,j}^k&=(2i-1,2j-1,2k-1)\\ u_i^k&=(2i-1,2d-1,2k-1)\ .\end{aligned}$$

We introduce some subsets of paths in $Z_{\mathrm{ext}}(\mathsf{T}_{\mathrm{cl,dec}^*})$. Let us denote by $\Delta(v,\ell)$ the set of all simple paths through internal vertices starting at $v \in \mathsf{V}^{\mathrm{ext,bottom}}_{\mathrm{cl,dec}^*}$ and ending at any vertex in $\mathsf{V}^{\mathrm{ext,top}}_{\mathrm{cl,dec}^*}$. Then, by the similar argument as for showing (70), we have

$$\left\{P \in Z_{\mathrm{ext}}(\mathsf{T}_{\mathrm{cl,dec}^*}) \mid |P| = \ell \text{ and } \langle\!\langle P, \mathcal{L}^*_{\mathrm{cl},Z}\rangle\!\rangle = 1\right\} = \bigcup_{v \in \mathsf{V}^{\mathrm{ext,bottom}}_{\mathrm{cl,dec}^*}} \Delta(v,\ell) \qquad \text{for} \qquad \ell \geq 1 \ ,$$

and the subsets of paths $\{\Delta(v,\ell)\}_{v\in \mathsf{V}^{\mathrm{ext,bottom}}_{\mathrm{cl,dec}^*}, \ell\geq 1}$ are pairwise disjoint. Let $L_v^{\min}$ and $L_v^{\max}$ be the minimal and maximal lengths of paths in $\bigcup_{\ell=1}^\infty \Delta(v,\ell)$ for $v \in \mathsf{V}^{\mathrm{ext,bottom}}_{\mathrm{cl,dec}^*}$, and let us define

$$\mathsf{V}^{\mathrm{ext,bottom}}_{\mathrm{cl,dec}^*}(s) = \left\{v \in \mathsf{V}^{\mathrm{ext,bottom}}_{\mathrm{cl,dec}^*} \mid L_v^{\min} = s\right\} \ .$$

It is easy to check from the definition of $\mathsf{T}_{\mathrm{cl,dec}^*}$ that for each vertex $v = (v_1, v_2, v_3) \in \mathsf{V}^{\mathrm{ext,bottom}}_{\mathrm{cl,dec}^*}$ there exists a path of length at most d which starts from v and ends at some $u \in \mathsf{V}^{\mathrm{ext,top}}_{\mathrm{cl,dec}^*}$ with $(v_1, v_3) = (u_1, u_3)$, and this guarantees that the set $\mathsf{V}^{\mathrm{ext,bottom}}_{\mathrm{cl,dec}^*}(s)$ is empty for all $s > d$. Moreover, we observe that $\mathsf{V}^{\mathrm{ext,bottom}}_{\mathrm{cl,dec}^*}(1)$ is empty. With an analogous argument as in the proof of Lemma 5.1, we have

$$\mathsf{res}_{\mathcal{L}^*_{\mathrm{cl},Z}}(p) = \sum_{s=2}^{d} B_s \qquad \text{where} \qquad B_s = \sum_{v \in \mathsf{V}^{\mathrm{ext,bottom}}_{\mathrm{cl,dec}^*}(s)} \sum_{\ell=s}^{L_v^{\max}} \binom{\ell}{\lceil \ell/2 \rceil} \cdot |\Delta(v,\ell)| \cdot p^{\lceil \ell/2\rceil} \ . \tag{124}$$

Here we note that each vertex in the graph $\mathsf{T}_{\mathrm{cl,dec}^*}$ has degree at most 6, and this implies similarly as before that

$$|\Delta(u,\ell)| \leq 5^\ell \qquad \text{for} \qquad u \in \mathsf{V}^{\mathrm{ext,bottom}}_{\mathrm{cl,dec}^*} \ . \tag{125}$$

We first calculate an upper bound on B_d. Due to the bound

$$\left|\mathsf{V}^{\mathrm{ext,bottom}}_{\mathrm{cl,dec}^*}(d)\right| \leq \left|\mathsf{V}^{\mathrm{ext,bottom}}_{\mathrm{cl,dec}^*}\right| \leq d \cdot \frac{R+1}{2}$$

on the number of external vertices on the bottom, together with the bound (125) and the fact that $\binom{\ell}{\lceil \ell/2\rceil} \leq 2^l$ and $p^{\lceil \ell/2\rceil} \leq p^{\ell/2}$, we have

$$\begin{aligned} B_d &\leq d \cdot \frac{R+1}{2} \sum_{\ell=d}^{\infty} 2^\ell \cdot 5^\ell \cdot p^{\ell/2} \\ &\leq d \cdot \frac{R+1}{2} \cdot \frac{q^d}{1-q} \qquad \text{where} \qquad q := 10\sqrt{p} \ . \end{aligned} \tag{126}$$

Here the geometric series converges since $0 \leq q = 10\sqrt{p} \leq \frac{1}{2}$

It remains to calculate an upper bound of $\sum_{s=2}^{d-1} B_s$. It is easy to see that

$$\left|\mathsf{V}^{\mathrm{ext,bottom}}_{\mathrm{cl,dec}^*}(s)\right| \leq 2 \cdot s \qquad \text{for} \qquad 2 \leq s \leq d-1 \ .$$

By analogous reasoning as for showing (126), we have

$$\begin{aligned} \sum_{s=2}^{d-1} B_s &\leq \sum_{s=2}^{d-1} \left(2 \cdot s \sum_{\ell=s}^{\infty} 2^\ell \cdot 5^\ell \cdot p^{\ell/2}\right) \\ &= \sum_{s=2}^{d-1} 2 \cdot s \cdot \frac{q^s}{1-q} \\ &\leq \frac{4q^2}{(1-q)^2} + \frac{2q^3}{(1-q)^3} \ . \end{aligned} \tag{127}$$

Again, the geometric series and the arithmetico-geometric series converge since $0 \leq q \leq \frac{1}{2}$. Inserting $q = 10\sqrt{p}$, (126) and (127) into (124), we obtain

$$\begin{aligned} \mathsf{res}_{\mathcal{L}^*_{\mathrm{cl},Z}}(p) &\leq \frac{4q^2}{(1-q)^2} + \frac{2q^3}{(1-q)^3} + d \cdot \frac{R+1}{2} \cdot \frac{q^d}{1-q} \\ &\leq \left[\frac{400}{(1-q)^2} + \frac{200q}{(1-q)^3} + 50d(R+1) \cdot \frac{(10\sqrt{p})^{d-2}}{1-q} \right] \cdot p. \end{aligned} \tag{128}$$

The bound (81) follows from (128) because $q \leq \frac{1}{2}$ and $\frac{1}{1-q} \leq 2$. □

Acknowledgements

SC and RK gratefully acknowledge support by the European Research Council under grant agreement no. 101001976 (project EQUIPTNT). Figures were produced using VESTA [9]. RK thanks Isaac Kim for discussions on surface codes.

References

[1] Sergey Bravyi, David Gosset, Robert Koenig, and Marco Tomamichel. Quantum advantage with noisy shallow circuits in 3D. Nature Physics, 16(10):1040–1045, October 2020.

[2] Eric Dennis, Alexei Kitaev, Andrew Landahl, and John Preskill. Topological quantum memory. Journal of Mathematical Physics, 43(9):4452–4505, September 2002.

[3] Jack Edmonds. Maximum matching and a polyhedron with 0, 1 vertices. J. of Res. the Nat. Bureau of Standards, 69 B:125–130, 1965.

[4] J. Eisert, K. Jacobs, P. Papadopoulos, and M. B. Plenio. Optimal local implementation of nonlocal quantum gates. Phys. Rev. A, 62:052317, Oct 2000.

[5] Austin G. Fowler. Proof of finite surface code threshold for matching. Physical Review Letters, 109(18):180502, November 2012.

[6] Harold N. Gabow. An Efficient Implementation of Edmonds' Algorithm for Maximum Matching on Graphs. Journal of the ACM, 23(2):221–234, April 1976.

[7] Daniel Gottesman and Isaac Chuang. Demonstrating the viability of universal quantum computation using teleportation and single-qubit operations. Nature, 402:390 – 393, 1999.

[8] E.L. Lawler. Combinatorial Optimization: Networks and Matroids. Dover Books on Mathematics Series. Dover Publications, 2001.

[9] Koichi Momma and Fujio Izumi. Vesta 3 for three-dimensional visualization of crystal, volumetric and morphology data. Journal of Applied Crystallography, 44, 12 2011.

[10] Christophe Piveteau and David Sutter. Circuit knitting with classical communication, April 2022.

[11] Robert Raussendorf, Sergey Bravyi, and Jim Harrington. Long-range quantum entanglement in noisy cluster states. Physical Review A, 71(6):062313, June 2005.

[12] Mary Beth Ruskai. Qubit entanglement breaking channels. Reviews in Mathematical Physics, 15(06):643–662, 2003.

[13] G. Vidal and C. M. Dawson. Universal quantum circuit for two-qubit transformations with three controlled-not gates. Phys. Rev. A, 69:010301, Jan 2004.

[14] D. S. Wang, A. G. Fowler, A. M. Stephens, and L. C. L. Hollenberg. Threshold error rates for the toric and surface codes, May 2009.
